# Results of multicenter double-blind placebo-controlled phase II clinical trial of Panagen preparation to evaluate its leukostimulatory activity and formation of the adaptive immune response in patients with stage II-IV breast cancer

**DOI:** 10.1186/s12885-015-1142-z

**Published:** 2015-03-13

**Authors:** Anastasia S Proskurina, Tatiana S Gvozdeva, Ekaterina A Alyamkina, Evgenia V Dolgova, Konstantin E Orishchenko, Valeriy P Nikolin, Nelly A Popova, Sergey V Sidorov, Elena R Chernykh, Alexandr A Ostanin, Olga Y Leplina, Victoria V Dvornichenko, Dmitriy M Ponomarenko, Galina S Soldatova, Nikolay A Varaksin, Tatiana G Ryabicheva, Stanislav N Zagrebelniy, Vladimir A Rogachev, Sergey S Bogachev, Mikhail A Shurdov

**Affiliations:** 1 Institute of Cytology and Genetics, Siberian Branch of the Russian Academy of Sciences, 10 Lavrentieva ave, Novosibirsk, 630090 Russia; 2 Novosibirsk State Medical University, Novosibirsk, 630091 Russia; 3 Novosibirsk State University, Novosibirsk, 630090 Russia; 4 Oncology Department of Municipal Hospital No 1, Novosibirsk, 630047 Russia; 5 Institute of Clinical Immunology, Siberian Branch of the Russian Academy of Medical Sciences, Novosibirsk, 630099 Russia; 6 Irkutsk State Medical Academy of Postgraduate Education, Irkutsk, 664049 Russia; 7 Regional Oncology Dispensary, Irkutsk, 664035 Russia; 8 Clinic Department of the Central Clinical Hospital, Siberian Branch of the Russian Academy of Sciences, Novosibirsk, 630090 Russia; 9 CJSC “Vector-best”, Koltsovo, Novosibirsk region, 630559 Russia; 10 LLC Panagen, Gorno-Altaisk, 649000 Russia

**Keywords:** Breast cancer, 5-fluorouracil, Doxorubicin, Cyclophosphamide, Leukostimulation, Adaptive immunity, dsDNA

## Abstract

**Background:**

We performed a multicenter, double-blind, placebo-controlled, phase II clinical trial of human dsDNA-based preparation Panagen in a tablet form. In total, 80 female patients with stage II-IV breast cancer were recruited.

**Methods:**

Patients received three consecutive FAC (5-fluorouracil, doxorubicin and cyclophosphamide) or AC (doxorubicin and cyclophosphamide) adjuvant chemotherapies (3 weeks per course) and 6 tablets of 5 mg Panagen or placebo daily (one tablet every 2–3 hours, 30 mg/day) for 18 days during each chemotherapy course. Statistical analysis was performed using Statistica 6.0 software, and non-parametric analyses, namely Wilcoxon-Mann–Whitney and paired Wilcoxon tests. To describe the results, the following parameters were used: number of observations (n), median, interquartile range, and minimum-maximum range.

**Results:**

Panagen displayed pronounced leukostimulatory and leukoprotective effects when combined with chemotherapy. In an ancillary protocol, anticancer effects of a tablet form of Panagen were analyzed. We show that Panagen helps maintain the pre-therapeutic activity level of innate antitumor immunity and induces formation of a peripheral pool of cytotoxic CD8+ perforin + T-cells. Our 3-year follow-up analysis demonstrates that 24% of patients who received Panagen relapsed or died after the therapy, as compared to 45% in the placebo cohort.

**Conclusions:**

The data collected in this trial set Panagen as a multi-faceted “all-in-one” medicine that is capable of simultaneously sustaining hematopoiesis, sparing the innate immune cells from adverse effects of three consecutive rounds of chemotherapy and boosting individual adaptive immunity. Its unique feature is that it is delivered via gastrointestinal tract and acts through the lymphoid system of intestinal mucosa. Taken together, maintenance of the initial levels of innate immunity, development of adaptive cytotoxic immune response and significantly reduced incidence of relapses 3 years after the therapy argue for the anticancer activity of Panagen.

**Trial registration:**

ClinicalTrials.gov NCT02115984 from 04/07/2014.

**Electronic supplementary material:**

The online version of this article (doi:10.1186/s12885-015-1142-z) contains supplementary material, which is available to authorized users.

## Background

Programmed chemotherapies involve tightly scheduled and dosed administration of highly toxic substances, whose therapeutic efficacy is invariably accompanied with systemic damage to the body. Liver and hematopoietic cells are the first to suffer from such therapies. Hence, when cancer patients are treated with cytostatic drugs, they routinely receive adjuvant medications alleviating the deleterious effects of cytostatics. Leukostimulatory drugs are among such protective agents [1].

Several classes of drugs are currently used to stimulate leukopoiesis. The first group includes the drugs boosting cellular metabolism – dicarbamin, methyluracil, pentoxyl, leukogen, etc. The second group comprises colony-stimulating growth factor analogs, such as filgrastim (neupogen), sagramostim, lenograstim, molgramostim (leucomax), etc. Chemical leukostimulatory drugs (dicarbamin and alike) are used in patients receiving myelosuppressive chemotherapy. In particular, dicarbamin stimulates maturation of neutrophilic granolocytes thereby reducing the occurrence of leukopenia and neutropenia. To treat severe leukopenia, analogs of human G-CSF, such as filgrastim and alike, are also widely used. These medications act by inducing mobilization of hematopoietic stem cells and by modulating production and release of neutrophils into peripheral blood. This panel of G-CSF-derived drugs is therefore used to treat various forms of neutropenia in cancer patients receiving myelosuppressive chemotherapy [1-3].

Recently, one more class of drugs which is based on nucleic acids has been introduced into oncology practice (ridostin, derinat, polydan, desoxynatum, etc.), as these drugs were reported to display, among others, leukostimulatory activity. Finally, one must consider a group of drugs that are based on CpG-modified DNA oligonucleotides, − these agents are used to induce adaptive antiviral and anticancer immune response. When tested in mice, these drugs resulted in 50-60% suppression of tumor growth [4-6].

By applying these research observations to the therapeutic activity of nucleic acids, we proceeded to develop Panagen medication, which is based on the fragmented human dsDNA, and is intended for use as an adjuvant leukostimulatory agent in cancer patients receiving multiple lines of chemotherapy. We put forward and test a novel concept of treating stage II-IV breast cancer by combining standard chemotherapy course with Panagen. This strategy allows protecting and activating the proliferation of hematopoietic stem cells along with expansion of the population of CD8 + perforin + cytotoxic T-cells, i.e. it aids in developing adaptive immune response in these patients.

Thus, Panagen is a multi-faceted drug with pronounced leukostimulatory activity and which functions to stimulate adaptive immune response across multiple courses of chemotherapy. In contrast to the above-mentioned classes of drugs (in particular, those G-CSF- and CpG-ODN- based), Panagen is manufactured in a form of tablets with gastro-resistant coating. This drug form is perfectly compatible with long-term therapy including three or more consecutive courses of chemotherapy (up to one year) without running the risks of adverse inflammatory and autoimmune reactions caused by the constant presence of dsDNA in the bloodstream. The drug mode of action is notably distinct from the mobilizing effect of colony-stimulating factors that induce abortive release of hematopoietic progenitors into the bloodstream. It is rather based on the activation of mucosal mononuclear cells. This is accompanied with secretion of stimulatory cytokines which induce proliferation of hematopoietic stem cells [7-10]. Of particular importance is that Panagen uniquely combines several therapeutic features, thereby paving the way to novel clinical applications.

## Methods

### DNA quantification in blood plasma of patients receiving tablet form of Panagen medication

Levels of DNA in blood plasma were determined according to the method described by Spirin [11]. This method relies on the optical density measurements of Panagen hydrolysis products, which translates into quantification of phosphorus content in nucleic acids. One milliliter of the medication (0.015 – 35 mkg of nucleic acids) is mixed with 5 ml of 1 N NaOH solution, and boiled on a waterbath for 5 minutes with stirring. The mixture is brought back to room temperature, transferred on ice and neutralized with concentrated perchloric acid. HClO_4_ is added to a final concentration of 1-2%, the reaction is vigorously mixed and centrifuged at +4°C 5000 g for 15 minutes. Supernatant containing ribomononucleotides is discarded and the pellet is re-dissolved in 5 ml 0.5 M HClO_4_. This is followed by heating on a boiling waterbath for 20 minutes. After chilling, the probes are centrifuged for 20 minutes at 5000 g. Supernatants are transferred into 10 mm pathlength cuvettes and optical density at 270 and 290 nm is measured using a spectrophotometer. Sample measurements are blanked against 0.5 M solution of perchloric acid.

Nucleic acid contentration (X, expressed as mkg/ml) is calculated using a formula: X = (D270 – D290) / 0.19 × 10.3, where D270 and D290 are optical density values, 0.19 is extinction coefficient, and 10.3 is a phosphorus-to-nucleic acid conversion factor. Notably, this method performs well when D260 and D270 values fall within 15% difference range of each other.

### Brief outline of phase II clinical trial protocol

Phase II clinical trial of preparation Panagen was approved by the Ministry of Health and Social Development of the Russian Federation (No. 47 of 03/12/2010) as well as by local ethics committees at the Irkutsk Regional Oncology Dispensary and the Novosibirsk Municipal Hospital No 1, where clinical trials were subsequently performed. The studies were carried out in compliance with the World Medical Association Declaration of Helsinki. Written informed consent to participate in the study was obtained from each of the patients, which specified open publication of the results presented as reports or otherwise. All patients were also insured.

The study recruited 80 female high-risk category patients with stage II breast cancer or patients with stage III-IV breast cancer who were advised to undergo chemotherapy. All patients were sequentially randomized, i.e. the patient was assigned to one of the two groups irrespective of the time she joined the trial. The first group comprising 57 patients received Panagen, of which 6 patients were later excluded from the study for various reasons, the second group received placebo (with 23 patients recruited, of which 6 were excluded during the trial). For ethical reasons, the second group was maximally down-sized so as to reliably meet the statistical significance threshold.

The patients received standard FAC chemotherapy (fluorouracil 500 mg/m^2^, doxorubicin 50 mg/m^2^, cyclophosphamide 500 mg/m^2^ – all i.v. for 1 day) or AC chemotherapy (doxorubicin 50 mg/m^2^ and cyclophosphamide 500 mg/m^2^ i.v. for 1 day). The patients completed three courses of chemotherapies (3 weeks per course), and each course began on the day of chemotherapy (day 1).

Patients received 5 mg Panagen or placebo tablets daily (6 tablets every 2–3 hours, 30 mg/day). The tablets were given to the patients 48 hours post-chemotherapy (day 3) and the course continued for 17 more days until day 20 post-chemotherapy. If the next round of chemotherapy was delayed, the patients stayed on Panagen or placebo. The patients stopped taking tablets one day before the next course of chemotherapy. Delay of next course of chemotherapy of up to one week was considered acceptable. All patients from the placebo cohort received standard-of-care therapy as required by the Ministry of Health and Social Development of the Russian Federation.

The clinical trial was conducted in two medical centers, Irkutsk Regional Oncology Dispensary (29 patients on FAC regimen), referred hereafter as “base I” and Novosibirsk Municipal Hospital No 1 (20 patients on FAC and 31 patients on AC regimens), which is referred to as “base II”.

Primary endpoints of the study were the degrees of leukopenia and neutropenia (grade 1, 2, 3, 4) which have emerged during the study.

Secondary endpoints of the study were:

- Occurrence of grade 1, 2, 3, 4 leukopenia during the cycles 1, 2, 3 of chemotherapy.
- Occurrence of grade 1, 2, 3, 4 neutropenia and febrile neutropenia during the cycles 1, 2, 3 of chemotherapy.
- Duration of grade 1, 2, 3, 4 leukopenia throughout chemotherapy cycles 1, 2, 3.
- Duration of grade 1, 2, 3, 4 neutropenia and febrile neutropenia throughout chemotherapy cycles 1, 2, 3.
- The lowest white blood cell count value observed across cycles 1, 2, 3.
- The lowest neutrophil count value observed across cycles 1, 2, 3.
- Time to restore leukocyte and neutrophil counts in cycles 1, 2, 3.
- Time from the start of chemotherapy to the point with lowest neutrophil and white blood cell counts in chemotherapy cycles 1, 2, 3.

If the patient was started on G-CSF medications, she was considered as having discontinued the study.

Statistical analysis was performed using Statistica 6.0 software, and non-parametric analyses, namely Wilcoxon-Mann–Whitney and paired Wilcoxon tests. To describe the results, the following parameters were used: number of observations (n), median, interquartile range, and minimum-maximum range.

For more details see Additional file 1.

### МТТ assay using human peripheral blood mononuclear cells

Cytotoxicity of human peripheral blood mononuclear cells (PBMCs) obtained by fractionation of patient peripheral blood on the density gradient of ficoll-urografin (d = 1.077 g/ml) was assayed against the MCF-7 tumor cell line. Tumor cells were placed in 96-well plates (5 × 10^4^ cells/well). Next, 5 × 10^4^, 10 × 10^4^ or 25 × 10^4^ PBMCs per well (1:1, 1:2 or 1:5 ratios) were added. Cell mixtures were incubated in RPMI-1640 supplemented with gentamicin sulphate (100 mkg/ml) in 5%о СО_2_ at 37°С for 21 h. Co-incubation was terminated by adding MTT solution to 0.5 mg/ml and reactions were allowed to stay for 3 more hours. Cells were centrifuged at 4000 rpm for 10 min (Eppendorf Centrifuge 5810 R). Supernatant was decanted and blue-colored formazan crystals were dissolved in 100 mkl DMSO. Optical density was read using «Multiskan RC» set at 570 nm with background subtraction measured at 620 nm. Results of the MTT assay were processed using Microsoft Excel 2002. Cytotoxicity index (CI) was calculated as follows:

$$ \mathrm{C}\mathrm{I}\ \left(\%\right) = \left[1-\left({\mathrm{OD}}_{\mathrm{e}+\mathrm{t}}-{\mathrm{OD}}_{\mathrm{e}}\right)/{\mathrm{OD}}_{\mathrm{t}}\right]\times 100 $$

where:

$$ \begin{array}{c}{\mathrm{OD}}_{\mathrm{e}+\mathrm{t}}\hbox{--}\ \mathrm{o}\mathrm{ptical}\ \mathrm{density}\ \mathrm{value}\ \mathrm{in}\ \mathrm{experimental}\ \mathrm{wells}\ \left(\mathrm{c}\mathrm{o}-\mathrm{incubated}\ \mathrm{effector}\ \mathrm{and}\ \mathrm{target}\ \mathrm{cells}\right);\\ {}{\mathrm{OD}}_{\mathrm{e}}\hbox{--}\ \mathrm{o}\mathrm{ptical}\ \mathrm{density}\ \mathrm{value}\ \mathrm{in}\ \mathrm{wells}\ \mathrm{with}\ \mathrm{effector}\ \mathrm{cells};\\ {}{\mathrm{OD}}_{\mathrm{t}}\hbox{--}\ \mathrm{o}\mathrm{ptical}\ \mathrm{density}\ \mathrm{value}\ \mathrm{in}\ \mathrm{wells}\ \mathrm{with}\ \mathrm{target}\ \mathrm{cells}.\end{array} $$

### Cytokine production by PBMCs isolated from recruited patients

To gain insight into the dynamics of cytokine production upon dsDNA administration, we used samples of peripheral blood from patients recruited into our trial. Blood samples were taken at three control timepoints, namely, at initial point – 1–3 days prior the first round of chemotherapy; at intermediate point – 1 day before the second course of chemotherapy; and at the final point – upon completion of the therapy (i.e. after completion of the third three-week course of Panagen).

To assay spontaneous cytokine production, peripheral venous blood was collected in heparin-containing vacutainers, and fresh 1 ml blood samples were reserved for assaying spontaneous cytokine production. In parallel, we also measured mitogen-induced cytokine production. For this purpose, we used a commercial kit “Cytokine-Stimul-Best” containing a mix of mitogens (PHA, Con A, LPS, − at 4, 4 and 2 mkg/ml each). The samples were incubated at 37°С for 24 hours. Cells were centrifuged at 10000 rpm for 3 min (Eppendorf Centrifuge 5810 R), the supernatants were transferred into new tubes, snap-frozen and stored at −70°С until further processed for quantification of cytokine production. Concentrations of IFN-γ, IFN-α, TNF-α, IL-1β, IL-6, IL-8, IL-10, IL-2, IL-17, VEGF, MCP, IL-18, IL-4, GM-CSF, G-CSF and IL-1 receptor antagonist (IL-1RА) in the samples were measured using solid-phase sandwich ELISA kits manufactured by the JSC «Vector-Best» (Novosibirsk, Russia).

SigmaStat statistical software package (Systat Software Inc., San Jose, CA, USA) was used for statistical data analysis. Two-way ANOVA followed by the Holman-Sidak test was used to analyze data in 2 groups x 3 intervals matrix to find dependencies between independent factors. A value of p < 0.05 was considered as statistically significant. Results are presented as Mean ± SE in their absolute units of measure (ng/μl). Absolute units of concentration were log10-converted prior to calculations of the stimulation index.

## Results and discussion

Below we describe the experimental data from both published reports and our own studies, which allowed us to design the strategy of cancer therapy with human dsDNA preparation Panagen as a leukostimulatory and leukoprotective agent, and as an activator of adaptive immune response.

### Choice of the drug’s active substance

Our choice of human dsDNA as an active substance in Panagen was dictated by both our experimental data and general knowledge of the interplay between dsDNA fragments and the genome of a human cell.

When dsDNA preparations from various sources were compared, human dsDNA consistently displayed superior leukostimulatory and anti-tumor activity [7-9,12-17].

Use of the DNA preparations and molecular interactions between DNA fragments and the cell genome are studied insufficiently, − mostly in yeast or in vitro. Recombination of extracellular DNA fragments has been reported as a likely event taking place in the nuclei of immune cells and various stem cells. Notably, the termini of these molecules induce activation of double-stranded DNA repair and recombination response in the cells [7,10,18-21]. Due to the presence of short stretches of homology on DNA ends, recombination may result in integration of exogenous DNA into the genome [22-32]. This translates to the conclusion that any xenogeneic DNA, even at low dosage, poses a threat to the integrity of the genome, as compared to the allogeneic DNA fragments, that are more likely to be considered as a suitable substrate for homologous recombination machinery, which in turn should lower the risk of introducing unwanted mutations.

The choice of specific size of DNA fragments to be used in the medication was based on the well-established fact that extracellular dsDNA is normally present in the human blood plasma and interstitial fluid at a concentration of 14–100 ng/ml, ranging 1–20 nucleosomal repeats in size, which equals to approximately 200 – 6000 bp [33-38].

Human placental DNA was selected as a source of active substance. Our protocol for collecting and isolating human DNA assures it is free of steroid hormones, various types of polysaccharides, infectious agents (parasites, protists, bacteria, RNA- and DNA-viruses), which is rigorously quality controlled for each batch of the drug (Registration certificate Medical Drugs of Russia No. 004429/08 of 09.06.2008). Furthermore, we make sure the drug is protein-free, as protein contaminations (for instance HMG proteins) are known to activate various types of immune and stem cells.

### Major features of the Panagen active substance in light of its possible therapeutic applications

1. *The fragments of exogenous extracellular dsDNA may interact and be internalized by various cell types without any transfection procedures.* It was established that double-stranded fragmented DNA molecules (including those used in Panagen) can be delivered into cell compartments without transfection – in both unconnected/loosely connected cells and in the tissue context (such as Peyer’s patches and solitary lymphatic nodules) [39-48]. Specifically, this property has been demonstrated for bone marrow cells, including mouse and human CD34+ hematopoietic progenitors tested in vivo, ex vivo cultured mouse and human bone marrow cells, and ascites forms of mouse hepatoma and lung carcinoma. DsDNA fragments were also shown to be incorporated by human pluripotent ES cells ex vivo, and by human breast adenocarcinoma cell line MCF-7, and may interact with human dendritic cells obtained *ex vivo* [7,10,18-21,49,50].
2. *Leukostimulatory effect of a tablet form of the drug (targeting CD34+ hematopoietic stem cells and their earliest lineage-commitment progeny)*. DsDNA fragments have been reported many times to target hematopoietic progenitors and so to boost their proliferation [14-17]. Leukostimulatory effect of the tablet form of human dsDNA preparation was consistently demonstrated on dogs [51] and in phase I clinical trial on healthy volunteers (unpublished data). This stimulatory effect on proliferation is apparently due to the incorporation of dsDNA by immunocompetent cells of the gut-associated lymphoid tissue, which stimulates their migration to the periphery [41-48] and concomitantly activates them to produce cytokines via the system of cytosolic DNA sensors [10,52,53]. Activated intestinal lymphocytes leave the gut and migrate to distant body regions, including bone marrow, where they are believed to induce proliferation of hematopoietic stem cells or their more committed progeny via direct cell-cell contacts or through secretion of specific cytokines.
3. *Activation of antigen-presenting dendritic cells and expansion of a population of cytotoxic perforin + CD8+ T cells contribute to the anticancer activity of Panagen.* These features are based on the interaction of Panagen dsDNA fragments with dendritic cells, which in turn activates their antigen-presenting properties [7-9,12,13].
4. *So-called “delayed death” phenomenon results from the selective targeting of CD34+ hematopoietic stem cells as they recover from the genotoxic stress caused by a cross-linking agent cyclophosphamide.* Fragments of exogenous dsDNA reach the nuclear interior of bone marrow cells, including CD34+ hematopoietic stem cells (HSCs). Importantly, if this happens during a very specific “death window” interval, the introduced DNA fragments overwhelm and interfere with the ongoing dsDNA repair. Thus, the dsDNA breaks waiting for delicate resolution via homology-dependent recombination pathway become instantly and randomly end-joined by an error-prone SOS-repair system. This leads to the failure of CD34+ HSC to differentiate into lymphoid lineage. Within several days, functional depletion of the organism immune system occurs, and animals succumb to opportunistic infections and progressive inflammatory response [18,20].
5. *Synergistic action of Panagen and cytostatic drugs cyclophosphamide and doxorubicin.* DNA-based immunomodulators have been shown to display synergistic effects with standard cytostatic drugs used in the clinics [4,54,55]. Consistently, we also reported that human dsDNA-based medication has a pronounced anti-cancer effect when combined with doxorubicin and cyclophosphamide [8,12,13].

### Choice of the tablet form of the drug and the strategy of drug administration

The full potential of Panagen activities which include leukostimulatory activity, activation of dendritic cells and stimulation of adaptive antitumor immunity, can only be exploited upon its long-term and continuous administration, so that it can efficiently act upon immune cells, particularly antigen-presenting cells. It has been reported in the literature and established in our own experiments that long-term presence of large amounts of dsDNA in the bloodstream of humans and experimental animals results in multiple inflammation foci in various organs and in activation of autoimmunity [18,56-61]. This has rendered the systemic route of administration – which is typically used in drugs with similar features (leukostimulation, leukoprotection and activation of protective immunity activation) quite problematic.

Yet, it was also known that dsDNA fragments administered *per os* can reach the immune cells residing in mucosal lymphatic system, where such cells become activated to produce a variety of cytokines and migrate elsewhere in the body [41-48]. So, we hypothesized that dsDNA fragments administered as tablets with gastro-resistant coating (Panagen) should activate immune cells in the gut, and this route of delivery could be exploited to ultimately target HSCs and antigen-presenting cells.

Our preclinical study performed in dogs [51] and phase I clinical trial of a tablet form of Panagen on 20 healthy volunteers indicated that this drug form stimulated leukopoiesis to the same extent as did intraperitoneal injections (unpublished data). Based on these data, we proceeded to phase II clinical trial on stage II-IV breast cancer patients.

Earlier studies of human dsDNA preparation Panagen have established it as a leukostimulatory agent. Taking into account its described synergistic activity with cytostatic drugs cyclophosphamide and doxorubicin to potently inhibit tumor growth in experimental animals, we developed a new therapeutic scheme of cytostatic treatment of human malignancies.

As was confirmed in multiple studies, leukostimulatory activity of dsDNA preparation is caused by the stimulation of bone marrow cell proliferation, in particular HSCs. This stimulatory effect may result from either internalization of dsDNA fragments by bone marrow progenitors or production of pro-proliferative cytokines by mononuclear cells activated by dsDNA fragments [10,18,41-48].

In terms of inducing adaptive (anticancer) immune response, the major steps of dsDNA therapeutic activity in combination with cross-linking and anthracycline cytostatics are as follows:

- Human dsDNA potently activates dendritic cells [7-9,12,13].
- Upon oral administration, dsDNA reaches the immune cells of intestinal mucosa and stimulates their professional properties [41-48].
- DsDNA fragments turn on the system of cytosolic sensors, thereby leading to production of specific cytokines by immune cells [10,53,54].
- Cyclophosphamide metabolite, phosphoramide mustard, induces formation of interstrand crosslinks in cancer cells, which leads to their death and production of tumor cell debris.
- Cyclophosphamide stimulates bone marrow-resident mononuclear cells to secrete interferon type I, dendritic cells undergo maturation and migrate to the periphery. Cyclophosphamide also drives cancer cells into apoptosis, which results in formation of immunogenic cell debris [62].
- Cyclophosphamide interferes with the functions of T-regulatory lymphocytes, resulting in their temporary depletion and functional suppression. In contrast, dendritic cells and cytotoxic T-cells are less sensitive to cyclophosphamide. Tumor cells then lose their cellular and humoral protection, whereas immunocompetent cells stop receiving inhibitory signaling from T-regulatory lymphocytes. This combination of factors makes it possible for the immune system to target the tumor [63-74].
- Anthracycline cytostatics, such as doxorubicin, induce membrane translocation of calreticulin in apoptotic cancer cells. This is interpreted as an “eat me” signal by the antigen-presenting cells, dendritic cells in particular. Similarly to cyclophosphamide, this results in formation of immunogenic tumor cell debris [75-78].

Taking into account the above-listed properties, we put forward a scientific basis for the following novel therapeutic strategy. Use of cyclophosphamide leads to the physical disintegration of tumor cells and immunogenic debris formation. Simultaneously, cyclophosphamide boosts maturation and peripheral migration of antigen-presenting dendritic cells. Doxorubicin also causes tumor cells to undergo apoptosis and induces membrane translocation of calreticulin in dying cells, which serves as an «eat me» signal for dendritic cells. These treatments converge to form immunogenic tumor cell debris. Additionally, cyclophosphamide selectively targets T-regulatory lymphocytes, and potently inhibits their functions or directly kills them. This leaves tumor cells unprotected from the immune system surveillance. Our studies indicate that following cyclophosphamide treatment and tumor cell lysis, administration of exogenous DNA independently of the cyclophosphamide activity will additionally stimulate activation of antigen-presenting properties of dendritic cells. This will be accompanied by suppression of T-regulatory lymphocytes that will no longer restrain the immune system from attacking the tumor. Such combined action of cyclophosphamide and doxorubicin will potentiate antigen uptake by dendritic cells activated by cyclophosphamide and dsDNA which in turn will launch specific anti-cancer immune response.

There are three key points to this strategy, as applied to the clinical practice:

1. Tablet form of the drug is administered 48 hours post cyclophosphamide treatment. This assures safety of the drug by avoiding the cell-destructive period, known as the “death window”.
2. The drug is administered continuously throughout the courses of chemotherapy. It is prescribed as a leukostimulatory medication used intermittently, continuously and massively, which mediates sustained activation of mucosal immune cells, and so results in increased proliferation of HSCs and their immediate committed progeny. Temporary drug withdrawal throughout the chemotherapy courses may only be required to avoid the “death window”.
3. Uninterrupted administration of the drug across multiple lines of chemotherapy allows combining its leukostimulatory potential with activation of antigen-presenting dendritic cells resident in the human mucosa. Cytostatic background further contributes to the maturation and release of CD4 + CD8 + perforin + cytotoxic T-cells into peripheral blood, which is generally accepted as developing adaptive immune response.

The proposed mode of action of the tablet form of Panagen relies on targeting the gut mucosa-resident lymphoid cells by dsDNA fragments. The active substance is encapsulated and delivered to the small intestine. The coating then disintegrates, and the substance is dissolved in the intestinal lumen. Dissolved dsDNA fragments reach mononuclear cells found in Peyer’s patches, in lymphoid follicles of vermiform appendix and in solitary follicles, where they activate the cells via a cascade of dsDNA sensors. Upon activation, various types of immune cells normally resident in gut-associated lymphoid tissue migrate into the bloodstream and reach immunocompetent organs. Immune cells then activate proliferation and mobilization of HSCs and their immediate committed progeny, via cell-cell contacts or secreted cytokines.

Gut-associated dendritic cells also migrate into the bloodstream upon activation. When they eventually reach and become anchored in the lymphoid organs (such as mesenterium), they are faced with cancer antigens in the form of immunogenic tumor cell debris. All these events culminate in the induction of anticancer adaptive immune response.

Here, we report on the results of phase II clinical trials of a human dsDNA-based preparation Panagen.

### DNA content in the blood plasma samples of patients receiving tablet form of Panagen

**Figure 1:**
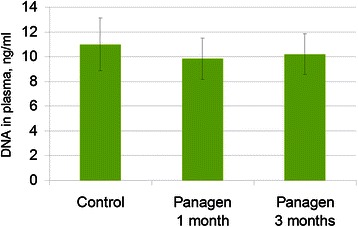
**DNA concentration in blood plasma of healthy volunteers not receiving Panagen (control, n = 15) and following daily oral administration of 30 mg Panagen for 1 and 3 months (n = 9).**

Panagen medication tested in the clinical trial is manufactured in the form of gastro-resistant tablets. This gastro-resistant coating dissolves at neutral pH in intestines, and so the active substance – fragmented human dsDNA – is liberated into intestinal lumen, where it reaches the mononuclear cells of Peyers patches [48]. Analyses of blood plasma samples from healthy donors daily receiving Panagen tablets for three months showed no increase in extracellular DNA concentration. Fasting blood samples were collected in the morning, 8 hours post-Panagen tablet. Daily dose was 30 mg, in six tablets (5 mg each) taken throughout the day, approximately 1 tablet every 2–3 hours (Figure 1). Furthermore, we detected no changes in DNA concentration in blood plasma 2 hours after swallowing 2–3 Panagen tablets (data not shown).

### Effects of Panagen on hematopoietic progenitors

Leukostimulatory effects of Panagen drug based on fragmented human dsDNA were analyzed throughout three consecutive courses of FAC or AC chemotherapies in patients with stage II-IV breast cancer (Additional files 2, 3 and 4).

Our primary goal at this step was to understand how the drug modulates different blood lineages under the increasing detrimental pressure of repeated chemotherapies. To do so, we measured specific blood lineage cell counts in peripheral blood at control points after 1, 2 and 3 rounds of chemotherapy in patients on Panagen vs placebo, and determined whether these values were significantly different. We assumed that positive effect of Panagen would be demonstrated if significant differences in blood cell counts are observed in at least one control point. This seemingly liberal definition of a positive effect was dictated by several factors. First, we found no published data describing and substantiating the specific time-points to assay the dynamics of hematopoiesis in response to gastrointestinal tract delivery of a drug – hence we were free to choose the control points. Second, neutrophils are known to quickly migrate from the periphery to their destination points, which makes it rather challenging to reliably measure their stimulated proliferation by analyzing peripheral blood samples. We also monitored the frequencies of stage I-IV neutropenia-related events throughout the chemotherapy courses, as well as the dynamics of CD34+/45+ HSCs, which was essentially a blind search in the absence of the documented time-course data.

**Figure 2:**
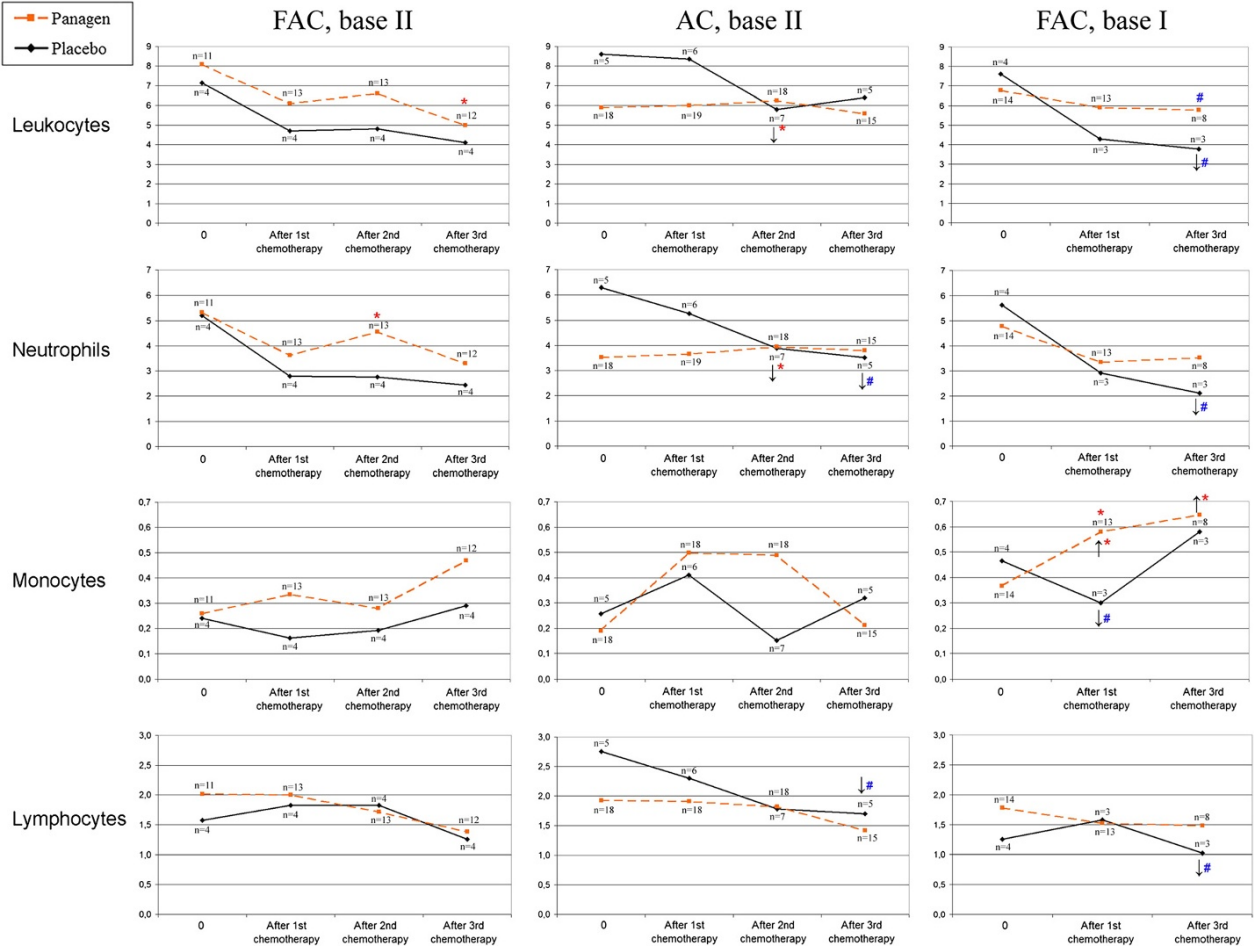
**Dynamic changes in blood cell counts (×10** ^**9**^ **cells/L) measured in the clinical trial at the initial pre-therapy timepoint (0) and on day 21 after each chemotherapy course.** Median values in each group are shown. The number of patients per group is indicated for each time point. Significantly higher values are observed for Panagen (dashed orange line) vs placebo (black solid line) groups of patients (Wilcoxon-Mann–Whitney test), as well as within each group relatively to the initial level before the therapy (Wilcoxon paired test). For patients who received Panagen, increased value is marked with up-facing arrow, for patients from placebo group, decreased value is highlighted by downward-facing arrow. Red asterisk (*) denotes significant values with р < 0.05, blue hash symbol (#) marks statistically significant difference with р < 0.11.

**Figure 3:**
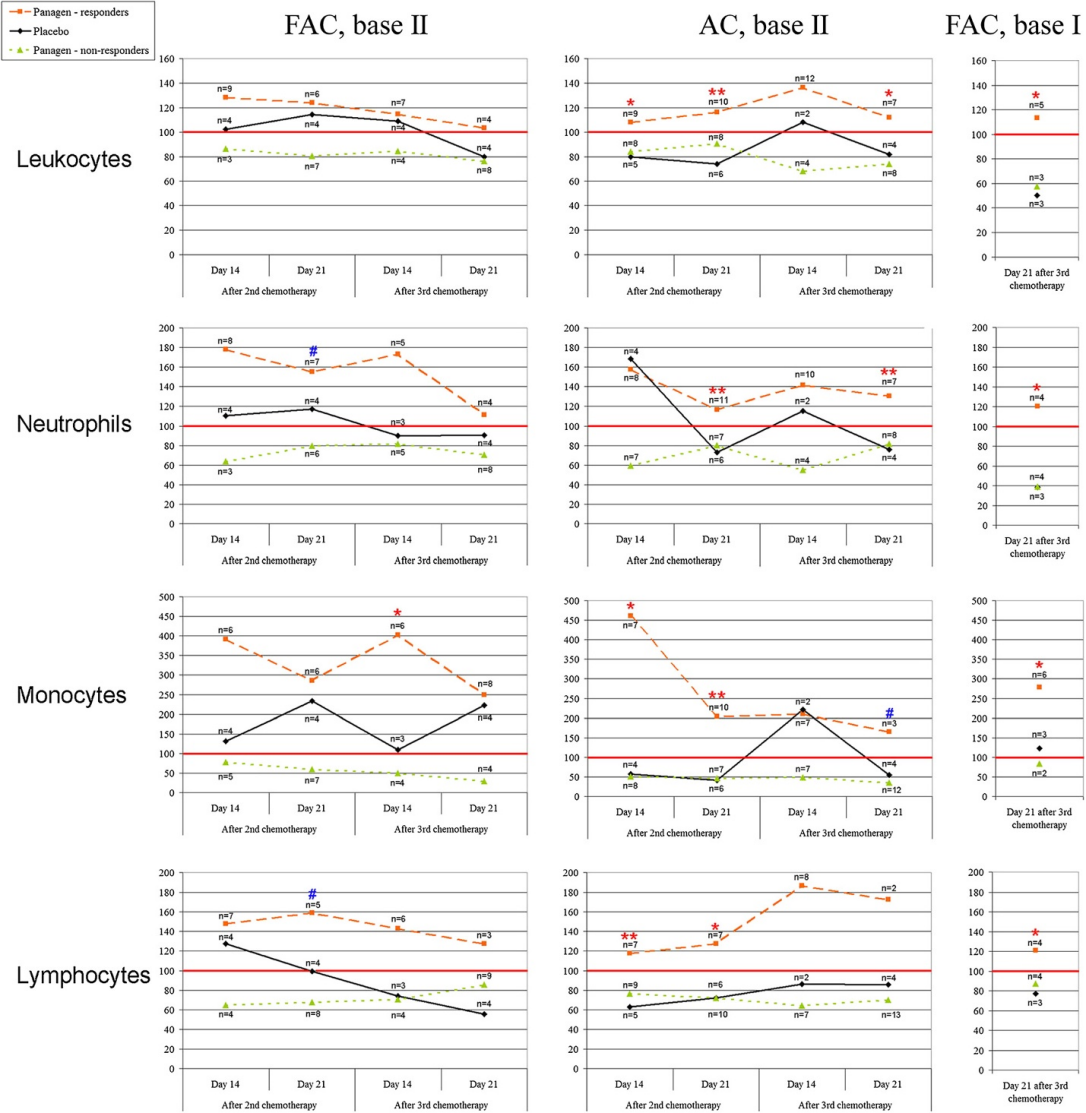
**Changes in stimulation indices (%) for blood cell types throughout three chemotherapy courses.** Median values per group are shown. The number of patients per group is indicated for each time point. Stimulation index is expressed as a ratio of values measured in second and third control time points (days 14 and 21) to the appropriate value observed in the control point of the first chemotherapy course (set as 100%). Patients were subgrouped into Panagen-responders, Placebo and Panagen-non-responders. Red line denotes 100% level, i.e. the values reported in control time points (days 14 and 21) after the first chemotherapy. Values that show statistically significant difference between Panagen-responders and Placebo patient groups (Wilcoxon-Mann–Whitney test) with р < 0.01 (**), р < 0.05 (*) and р < 0.09 (#) are highlighted.

The analysis performed thus far summarizes the following therapeutic features of Panagen in the context of three courses of FAC/AC chemotherapies. We demonstrate that absolute cell counts for lymphocytes, neutrophils and monocytes in control points on day 21 after 1, 2 and 3 chemotherapy courses are significantly different between Panagen and placebo-treated patient cohorts (Figure 2). In order to mitigate the confounding effects from individual patients on statistical analysis of Panagen leukostimulatory activity (as assayed by cell counts in peripheral blood, by timing and magnitude of cell proliferation), the patients were grouped into Panagen-responders and non-responders (Figure 3, see appropriate parts of Additional files 2, 3 and 4). Patients whose cell counts were higher at a given time point than on day 14 or day 21 after the first course of chemotherapy (set as 100%) were classified as responders. Most of the blood parameters in the group of Panagen-responders were significantly higher than in the placebo cohort. Notably, 52% of patients positively responded on Panagen therapy throughout the 3 courses of chemotherapy as measured in control points. This approach allowed us to accurately delineate the leukostimulatory effect of Panagen with minimal contribution of individual patient-specific effects.

Notably, blood test parameters in the placebo group display statistically significant differences when compared to the first control point (Figure 2). If one compares cell count curves for placebo and Panagen patient groups, most of the data points (for leukocytes, neutrophils and lymphocytes) display pronounced decline by the end of the third round of chemotherapy in placebo-, but not in Panagen-treated patients where they remain at initial levels (Figure 2). These data are consistent with protective effect of Panagen on leukocyte progenitors.

**Figure 4:**
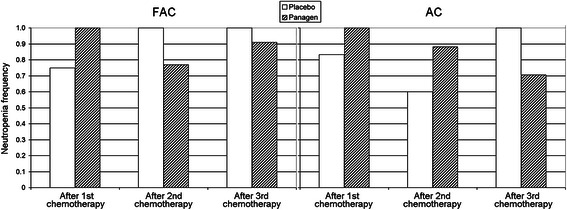
**Frequency of grade I-IV neutropenia-related events in patients at base II on day 14 of three courses of FAC and AC chemotherapies.**

In both FAC and AC chemotherapies, we observed progressively fewer neutropenias in Panagen patients facing the increasingly negative effects of chemotherapies, as compared to the placebo cohort, where the frequency of neutropenias increased (Figure 4) (Additional file 2, p. 13–15; Additional file 3, p. 15–16).

Panagen alters the timing of cyclophosphamide-induced abortive release of CD34+/45+ HSCs into peripheral blood. Notably, Panagen also significantly increases the number of HSCs mobilized into the bloodstream (Additional file 5).

As it follows from our analysis, Panagen potently stimulates erythropoietic lineage in patients on FAC protocol. Hemoglobin levels generally dropped in patients on chemotherapy, yet this was only observed in 63% patients receiving Panagen vs 100% placebo-treated patients. Further, in 23% patients receiving Panagen, we saw an increase in platelet counts by day 21 (Additional file 2, p. 27–29).

Panagen shows hepatoprotective activity counteracting the activities of chemotherapeutic drugs cyclophosphamide, doxorubicin and fluorouracil (Additional file 2, p. 32–38; Additional file 3, p. 38–50; Additional file 4, p. 16–24). Panagen suppresses the effects of drug-induced immunodeficiency in patients on the AC protocol of breast cancer chemotherapy (Additional file 3, p. 51–52). Panagen activity positively correlates with regeneration of surface epithelium, which is likely due to increased proliferation of basal cells in the skin (Additional file 2, p. 39–42).

### Activation of immune response. Effects of Panagen on patient cytokine profiles

When combined with cytostatic drugs, Panagen increases the number of CD8 + perforin + cytotoxic T cells in peripheral blood, which serves as a major marker of mounting adaptive immune response (Additional file 6).

In our earlier studies, we established that fragmented genomic DNA is actively targeting dendritic cells and potently induces their allostimulatory activity and maturation both *ex vivo* and *in vivo* [7,9]. We also showed that when combined with cyclophosphamide, fragmented dsDNA preparation displays pronounced anti-cancer activity *in vivo* in mice with tumor grafts [12]. When fragmented dsDNA was injected in tumor-engrafted mice following cyclophosphamide or cyclophosphamide and doxorubicin administration, significant antitumor activity was observed [13]. We speculate that the most likely scenario describing suppression of tumor growth in these *in vivo* experiments involves activation of key immune system components, namely that of adaptive immunity, which is primarily characterized by production of CD8 + perforin + cytotoxic T cells [8]. We can not formally exclude yet another option, i.e. two-pronged targeting of cancer cells by immune system and via direct cytotoxic activity of dsDNA preparation [79].

In order to firmly establish whether the anticancer immune response does unfold upon combined use of fragmented exogenous dsDNA and cytostatics, we surveyed basic cell types involved in adaptive immunity. Namely, we monitored the dynamics of plasmacytoid and myeloid dendritic cells, T-regulatory lymphocytes and CD8+ perforin + cytotoxic T-cells. Our analysis failed to uncover pronounced trends when measuring dendritic cell and T-regulatory lymphocyte counts.

**Figure 5:**
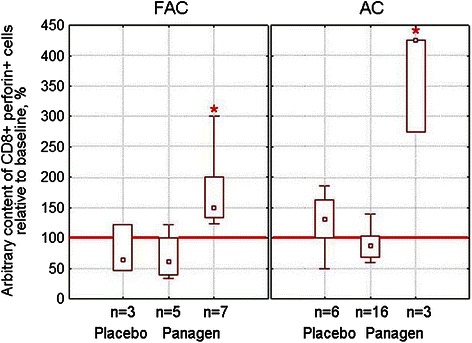
**Arbitrary content (%) of CD8+ perforin + cytotoxic T-cells in peripheral blood of patients at base II undergoing FAC or AC chemotherapy on day 21 after the first course, relatively to the initial baseline level (100%, red line).** Panagen group is split to demonstrate that two distinct patient subgroups are present – “responders” (those having cell counts significantly different from the placebo group) and “non-responders”. Median values, quartile range 25-75% (box) and minimum-maximum range are given for each group; n – the number of patients per group. Significant differences from the Placebo group with p < 0.05 (Wilcoxon-Mann–Whitney) are marked with red asterisk.

We observed significant increase in CD8+ perforin + cytotoxic T-cells in the peripheral blood of patients receiving Panagen vs placebo on day 21 following 1 course of chemotherapy, notably 58% patients on FAC protocol (7 out of 12) and 16% patients on AC protocol (3 out of 19) responded (Figure 5). This supports the activating role of Panagen on development of adaptive immune response when it is combined with standard FAC and AC chemotherapeutics.

It was not unexpected that our analysis of peripheral blood cell counts would fail to uncover significant expansion of dendritic cell population, whose maturation and activation was driven by Panagen. In fact, dendritic cells are known not to stay freely circulating in the peripheral blood for too long, as they quickly anchor in the lymphoid organs (first and foremost in the mesenterium). Furthermore, we could not tell in advance the exact time when matured dendritic cells would likely peak in the peripheral blood. These two points contributed to our failure to detect the changes in peripheral dendritic cell counts. Nonetheless, the observed increase in cytotoxic T cells argued for the activation of a substantial proportion of dendritic cell population capable of efficient antigen presentation so that specific adaptive immune response could be unfolded. We speculate that during the cytostatic therapy the largest source of cancer antigens comes from immunogenic debris of cancer cells killed by cyclophosphamide and doxorubicin treatments. This may suggest that adaptive immune response formed is a highly personalized adaptive anticancer immune response.

**Figure 6:**
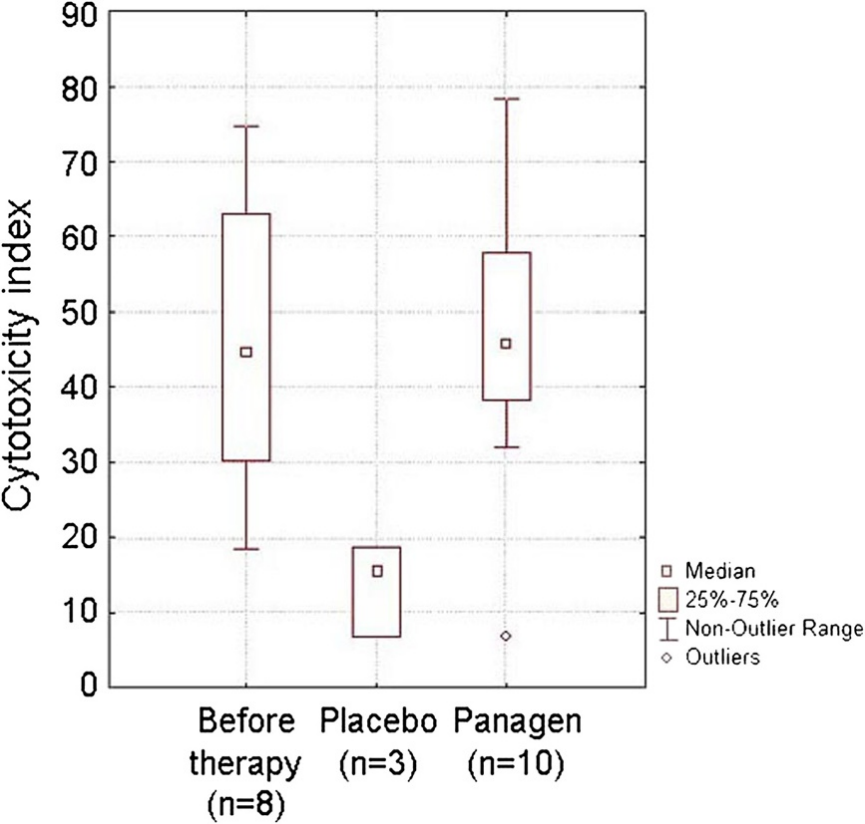
**Comparative analysis of cytotoxicity indices in PBMCs of patients (FAC regimen, base II) on day 21 following the third round of chemotherapy.** Largely responsible for innate anticancer immunity, PBMCs retain their specific functions at the levels observed before the therapy in patients receiving Panagen throughout three courses of chemotherapy (р < 0.05). Unlike in Panagen cohort, PBMCs from placebo group patients display three-fold decrease in cytotoxicity indices by the end of the third chemotherapy course relatively to the initial level.

Clearly, Panagen potently protects PBMCs known to mediate innate anticancer immunity and counterbalances the negative effects from three courses of aggressive chemotherapy. We further analyzed Panagen activity to maintain and enhance proliferation of PBMCs in the context of innate anticancer immunity. We chose to analyze non-specific cytotoxic activity of patient-derived PBMCs against human adenocarcinoma cell line MCF-7. Our results indicate that Panagen has protective and stimulatory activity towards PBMCs. Cytotoxic indices of PBMCs in patients receiving Panagen were significantly higher than those observed in the placebo group (Figure 6).

**Figure 7:**
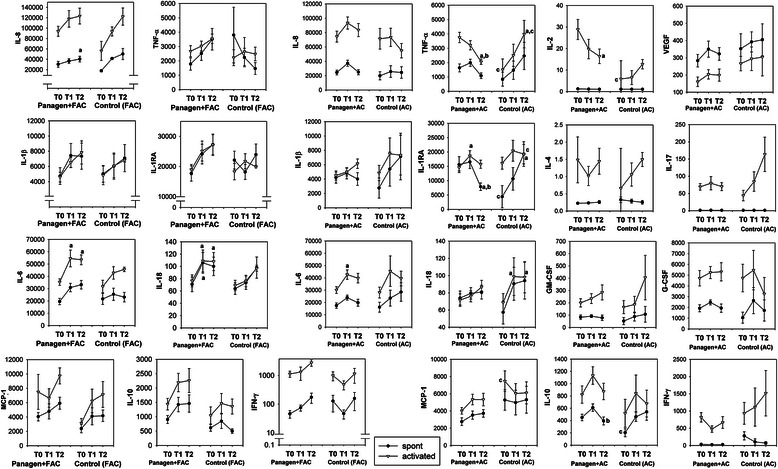
**Effects of Panagen on patient cytokine profiles.** Comparison of spontaneous and mitogen-stimulated cytokine secretion in Control (n = 4) and Panagen-treated (n = 12) patients under FAC chemotherapy regimen and in Control (n = 6) and Panagen-treated (n = 19) patients under AC chemotherapy regimen. T0 – before the therapy, Т1 – day 21 after the first chemotherapy, Т2 – day 21 after the third chemotherapy. Results were analyzed by One Way Repeated Measures and Two Way ANOVA with post hoc Holm-Sidak and Tukey tests in order to evaluate significance of interval-dependent changes, as well as difference between groups. Absolute units of measure (pg/ml) were converted to natural logarithms in order to normalize data prior to analysis. Data presented as Mean ± SEM; a,b – statistically significant difference (p < 0.05) vs T0 or T1 interval respectively; c – statistically significant difference (p < 0.05) between Panagen and Control groups.

Opposing trends in the production of TNF-α, IL-2, IL-1, IL-1RA, IL-18, IL-10 and IFN-γ between Panagen-treated and placebo groups have been observed. The production of IL-1, IL-1RA, IL-18, IL-10 and IFN-γ in the Panagen group of patients decreased, while the production of the same cytokines in the placebo group increased. In contrast, production of TNF-α and IL-2 in Panagen-treated patients progressively declined. Moreover, those changes were statistically significant (Figure 7). As per respect to physiological significance, it is essential to remember that an increase in systemic levels of these two cytokines is often associated with initial stages of a cytokine storm. In general, decreased ability to secrete cytokines is associated with immune suppression. However, increase in systemic cytokine production is not necessarily a good sign. Uncontrolled systemic secretion of cytokines is one of the major pathogenic outcomes of septic shock and systemic inflammatory response syndrome. It is usually associated with the rapid and severe increase in circulating levels of IL-6, IL-8, MCP-1, MIP-1β, IFN-γ, GM-CSF (also known as “cytokine storm”) due to polyclonal activation of immunocompetent cells [80-84].

Additionally, our quantitative analysis demonstrates unusually high cytokine secretion by PBMCs as compared with samples from healthy donors [85], which may argue for non-specific activation of leukocytes by tumor-derived factors or by the therapy performed, which should similarly converge in a systemic response presenting as a cytokine storm. Lower cytokine secretion upon Panagen co-administration may be indicative of efficient suppression of inflammatory reaction. Collectively, these results argue for cytoprotective properties of Panagen.

Previously it was established that in mice cyclophosphamide monotherapy stimulates maturation and peripheral migration of dendritic cells, as well as induces bone marrow cells to secrete type I interferon [62]. Concomitantly, cyclophosphamide treatment results in cancer cell apoptosis, thereby producing immunogenic cancer cell debris. Thus, anticancer activity of cyclophosphamide relies on a combination of direct cancer cell destruction and activation of adaptive anticancer immunity.

Our studies [7-10] show that dsDNA works in parallel to cyclophosphamide and independently boosts adaptive immunity. So, the combination of dsDNA and cyclophosphamide results in maximum immune response, which is manifested as significant increase in cytotoxic T cells in peripheral blood samples of the recruited patients Taken together, published experimental data and results of the present clinical trial argue for a highly complex nature of anticancer immune response formation upon synergistic activity of standard chemotherapy (FAC, AC) with Panagen dsDNA preparation.

### Long-term follow-up analysis

**Figure 8:**
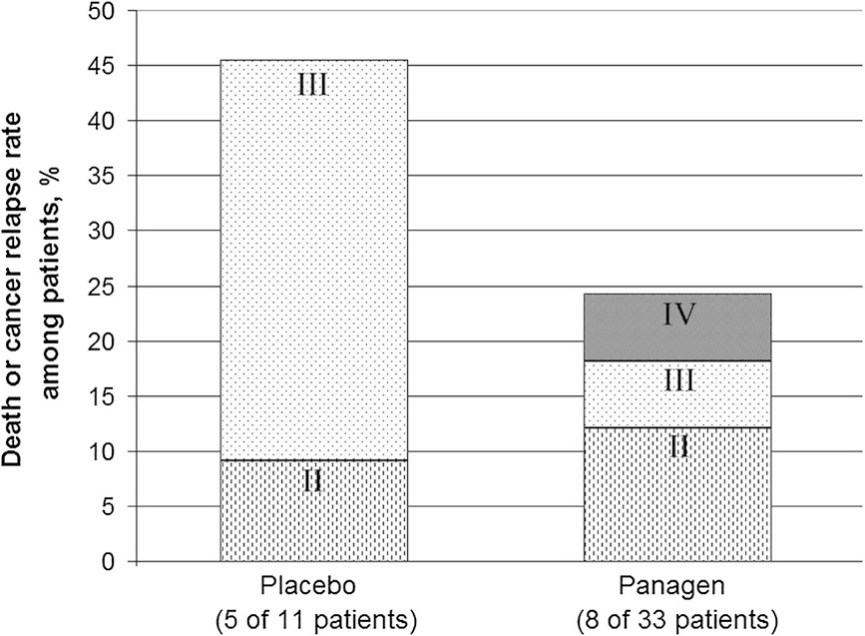
**3-year follow-up analysis of Panagen clinical trial.** Percentage of relapse events and deaths of patients relatively to the total number of patients. Data for patients from the base II (FAC and AC regimen) are presented. Each group of bars is labeled to show the percentage of patients having stage II, III or IV cancers.

We compared the frequency of relapses in patients of both study groups 3 years following the therapy at base II (18 FAC patients and 26 AC patients). In Panagen vs placebo cohorts, 24% and 45% of patients, respectively, relapsed or died (Figure 8, Table 1). Notably, in the Panagen cohort, 2 out of 8 study participants with cancer relapse had initially stage IV cancer with metastases, and 2 more showed evidence of cancer progression during the first or second rounds of chemotherapy. In other words, these patients had disseminated cancer very early in the therapy, and so formally Panagen therapy was used to treat the patients whose cancer stage was beyond the protocol coverage.

**Table 1:** **Long-term follow-up analysis**

Individual patient number	Cancer stage prior to treatment		1 year	1.5 year	2 years	3 years
	TNM	AJCC				
**Panagen group**						
**02-01**	T4NxM0	IIIB	ND^1^	Alive	Alive	**Progression. Metastases in lungs**
02-02	T2N0M0	IIA	Remission	Remission	Remission	ND
**02-03**	T1N1M0	IIA	Remission	Remission	Remission	**Progression. Metastases in Th11**
**02-04**	T3N2M1	IV	**Deceased. Cause: breast cancer**	-	-	-
02-05	T4N1M0	IIIB	Remission	Remission	Remission	ND
**02-06**	T4NxM1	IV	**Deceased. Cause: breast cancer**	-	-	-
02-08	T4N1M0	IIIB	Remission	Remission	Remission	ND
**02-09**	T1N1M0	IIA	**Progression. Bone metastases (detected after 1st chemotherapy)**	Alive	Alive	ND
02-10	T1N1M0	IIA	Remission	Remission	Remission	Remission
02-11*	T2N1M0	IIB	Remission	Remission	Remission	Remission
02-14	T2N2M0dex T4N1M0sin	IIIB	Remission	Remission	Remission	Remission
02-15	T3N2M0	IIIA	Remission	Remission	Remission	Remission
02-16*	T4NxM0	IIIB	Remission	**Cancer relapse. Skin metastases around scar tissue**	Partial regression	ND
02-18*	T4NxM0	IIIB	ND	Alive	Alive	ND
02-20	T2N1M0	IIB	Remission	Remission	Remission	
02-21	T2N0M0	IIA	Remission	Remission	Remission	
02-22*	T2N1M0	IIB	Remission	Remission	Remission	
02-24	T2N1M0	IIB	Remission	Remission	Remission	
02-25*	T2N2M0	IIIA	Remission	Remission	Remission	
02-26	T1N1M0	IIA	Remission	Remission	Remission	
02-27	T1N2M0	IIIA	Remission	Remission	ND	
02-28*	T2N1M0	IIB	Remission	Remission	ND	
02-29	T2N0M0	IIA	Remission	Remission	ND	
02-30	T2NxM0	II	Remission	Remission	Remission	
**02-31**	T2N1M0	IIB	**Progression. Metastases in lungs**	Alive	Alive	
02-33*	T2N3M0	IIIC	Remission	ND	ND	
02-36	T4NxM0dex T2NxM0sin	IIIB	Treatment is on-going due to chemotherapy scheduling issues	Treatment in progress	ND	
02-39	T2N3M0	IIIC	Remission	Remission	Remission	
02-40*	T2N1M0	IIB	Remission	ND	ND	
02-42*	T1N3M0	IIIC	Remission	Remission	Remission	
02-43	T3N0M0	IIB	Remission	Remission	Remission	
02-44*	T1N1M0	IIA	**Progression. Bone metastases (detected after 2nd chemotherapy)**	ND	ND	
02-45*	T4N1M0	IIIB	Remission	Remission	Remission	
*Final outcome*			*Progression or death in* ***8 out of*** 33 *patients (24%)*			
**Placebo group**						
**02-07**	T4N2M0	IIIB	**Deceased. Cause: breast cancer**	-	-	-
02-12*	T2N2M0	IIIA	Remission	Remission	Remission	ND
**02-13**	T4N3M0	IIIC	Treatment in progress	**Progression. Metastases in lungs**	ND	ND
02-17	T2N1M0	IIB	Remission	Remission	Remission	Remission
02-23*	T2N1M0	IIB	**Progression. Bone metastases**	Alive	ND	
02-32	T4NxM0	IIIB	ND	ND	ND	
02-34	T4N2M0	IIIB	ND	ND	ND	
02-35*	T4NxM0	IIIB	Remission	Remission	**Progression. Metastases in brain**	
02-37*	T4N0M0	IIIB	**Progression. Metastases in brain, lungs, liver and bones**	ND	ND	
02-38	T2N2M0	IIIA	ND	ND	ND	
02-41	T1N1M0	IIA	Remission	Remission	Remission	
*Final outcome*			*Progression or death in* ***5 out of 11*** *patients (45%)*			

Note: Cancer progression (metastatic disease or patient death) is highlighted by boldface.

^1^– ND – no data available.

*– estrogen-dependent tumors.

## Conclusions

1. Human dsDNA-based drug Panagen shows leukoprotective and leukostimulatory activity when assessed throughout three consecutive FAC and AC chemotherapies.
2. Panagen efficiently protects the cells involved in innate anticancer immunity from detrimental effects of FAC and AC chemotherapies
3. Panagen induces adaptive immune response, as assayed by production of CD8 + perforin + cytotoxic T cell population.
4. Panagen therapy reduces the 3-year relapse frequency from 45% down to 24%.

The data collected in this trial set Panagen as a multi-faceted “all-in-one” medicine that is capable of simultaneously sustaining hematopoiesis, protecting the innate immune cells from toxic effects of three consecutive rounds of chemotherapy and boosting individual adaptive immunity. Its unique feature is that it is delivered via gastrointestinal tract and acts through the lymphoid system of intestinal mucosa. Taken together, maintenance of the initial level of innate immunity, development of adaptive cytotoxic immune response and significantly reduced incidence of relapses 3 years after the therapy argue for the anticancer activity of Panagen.

## Additional files

Additional file 1:
**Progress of phase II clinical trials of Panagen.**

Additional file 2:
**Results of the completed phase II double-blind multicenter placebo-controlled clinical trial to evaluate the safety and leukostimulatory activity of Panagen in breast cancer patients.** The study was performed at the Oncology Department of Novosibirsk Municipal Hospital No 1 and included 18 patients receiving FAC therapy (14 patients additionally received Panagen, 4 patients received placebo).
Additional file 3:
**Results of the phase II double-blind multicenter placebo-controlled clinical trial to evaluate the safety and leukostimulatory activity of Panagen in breast cancer patients.**The study was performed at the Oncology Department of Novosibirsk Municipal Hospital No 1 and included 26 patients receiving AC therapy (19 patients additionally received Panagen, 7 patients received placebo).
Additional file 4:
**Results of the phase II double-blind multicenter placebo-controlled clinical trial to evaluate the safety and leukostimulatory activity of Panagen in breast cancer patients.** The study was performed at the Irkutsk Regional Oncology Dispensary and included 23 patients receiving FAC therapy (18 patients additionally received Panagen, 5 patients received placebo).
Additional file 5:
**Analysis of CD34+/45+ progenitor cell dynamics at control time points throughout the three cycles of chemotherapy.**

Additional file 6:
**Summary of experiments characterizing development of adaptive immune response.**

## Abbreviations

AC chemotherapy: Chemotherapy including doxorubicin and cyclophosphamide
dsDNA: Double-stranded DNA
FAC chemotherapy: Chemotherapy including 5-fluorouracil, doxorubicin and cyclophosphamide
HSCs: Hematopoietic stem cells
PBMCs: Peripheral blood mononuclear cells
