# Erratum to: Results of multicenter double-blind placebo-controlled phase II clinical trial of Panagen preparation to evaluate its leukostimulatory activity and formation of the adaptive immune response in patients with stage II-IV breast cancer

**DOI:** 10.1186/s12885-016-2163-y

**Published:** 2016-02-17

**Authors:** Anastasia S. Proskurina, Tatiana S. Gvozdeva, Ekaterina A. Alyamkina, Evgenia V. Dolgova, Konstantin E. Orishchenko, Valeriy P. Nikolin, Nelly A. Popova, Sergey V. Sidorov, Elena R. Chernykh, Alexandr A. Ostanin, Olga Y. Leplina, Victoria V. Dvornichenko, Dmitriy M. Ponomarenko, Galina S. Soldatova, Nikolay A. Varaksin, Tatiana G. Ryabicheva, Peter N. Uchakin, Stanislav N. Zagrebelniy, Vladimir A. Rogachev, Sergey S. Bogachev, Mikhail A. Shurdov

**Affiliations:** Institute of Cytology and Genetics, Siberian Branch of the Russian Academy of Sciences, Novosibirsk, 630090 Russia; Novosibirsk State Medical University, Novosibirsk, 630091 Russia; Novosibirsk State University, Novosibirsk, 630090 Russia; Oncology Department of Municipal Hospital No 1, Novosibirsk, 630047 Russia; Institute of Clinical Immunology, Siberian Branch of the Russian Academy of Medical Sciences, Novosibirsk, 630099 Russia; Irkutsk State Medical Academy of Postgraduate Education, Irkutsk, 664049 Russia; Regional Oncology Dispensary, Irkutsk, 664035 Russia; Clinic Department of the Central Clinical Hospital, Siberian Branch of the Russian Academy of Sciences, Novosibirsk, 630090 Russia; CJSC «Vector-best», Koltsovo, Novosibirsk region 630559 Russia; Mercer University School of Medicine, Macon, Georgia 31207 USA; LLC 1 Panagen, Gorno-Altaisk, 649000 Russia

## Erratum

After publication of this work [1], we noted that we inadvertently failed to include the complete list of all co-authors. In this erratum we rectify these mistakes. The full list of authors and the updated Authors Contributions section are reported below. We apologize for any inconvenience this oversight may have caused.

Corrected Authors’ List:

Anastasia S. Proskurina, Tatiana S. Gvozdeva, Ekaterina A. Alyamkina, Evgenia V. Dolgova, Konstantin E. Orishchenko, Valeriy P. Nikolin, Nelly A. Popova, Sergey V. Sidorov, Elena R. Chernykh, Alexandr A. Ostanin, Olga Y. Leplina, Victoria V. Dvornichenko, Dmitriy M. Ponomarenko, Galina S. Soldatova, Nikolay A. Varaksin, Tatiana G. Ryabicheva, Peter N. Uchakin, Stanislav N. Zagrebelniy, Vladimir A. Rogachev, Sergey S. Bogachev, and Mikhail A. Shurdov.
