# Supplementary material for: Results of multicenter double-blind placebo-controlled phase II clinical trial of Panagen preparation to evaluate its leukostimulatory activity and formation of the adaptive immune response in patients with stage II-IV breast cancer
Source: BMC Cancer. 2015 Mar 13;15:122. doi: 10.1186/s12885-015-1142-z (PMC4365563; doi:10.1186/s12885-015-1142-z)
Supplement: Additional file 1: — Progress of phase II clinical trials of Panagen. [file 12885_2015_1142_MOESM1_ESM.pdf]

Additional file 1. Progress of phase II clinical trials of Panagen

Documentation

Phase II clinical trial of preparation Panagen was performed in compliance with the following documentation:

- Approval of the Ethics Committee of the Federal Service on Surveillance in Healthcare and Social Development of Russian Federation (Protocol No. 7 of 04/08/2008) to carry out clinical trial of preparation Panagen.
- Approval of the Ministry of Health and Social Development of the Russian Federation (MH&SD) No. 209 of 05/12/2008 to perform phase I clinical trial of preparation Panagen.
- Board Decision of the Federal State Agency «Scientific Center for Evaluation of Medical Products» affiliated with the Federal Service on Surveillance in Healthcare and Social Development of Russian Federation (Protocol No. 15 of 07/24/2009) to approve the results of phase I clinical trial of preparation Panagen.
- Board Decision of the Federal State Agency «Scientific Center for Evaluation of Medical Products» affiliated with the Federal Service on Surveillance in Healthcare and Social Development of Russian Federation (Protocol No. 15 of 07/24/2009) to perform phase II clinical trial of preparation Panagen.
- Approval of the MH&SD No. 47 of 03/12/2010 to perform phase II clinical trial of preparation Panagen.
- Protocol adaptation following the MH&SD decision, letter No. 276822-31-1 of 09/08/2011.
- Registration certificate “Medical Drugs of Russia” for the active pharmaceutical ingredient of the preparation Panagen No. 004429/08 of 06/09/2008.
- Product monograph for the active pharmaceutical ingredient of Panagen No. 42-8621-07.

Paradigm of controlled neutropenia

One of the drawbacks associated with cytostatic therapy is uncontrolled grade IV neutropenia which progresses into febrile neutropenia and is frequently associated with poor prognosis in patients with cancer.

In course of our pilot studies performed over the past several years, we established that Panagen helps maintain viability of neutrophil progenitors and stimulates their proliferation even upon severe drop in neutrophil counts in peripheral blood. For this reason, we suggested that use of Panagen may help restore this cell population when neutrophil counts are reduced down to the levels that require obligate leukostimulatory therapeutic intervention (<500 neurophils/ml blood).

We put forward a therapeutic paradigm of controlled grade IV neutropenia. This paradigm posits that when Panagen is used in a tablet form and according to the specified regimen, no additional leukostimulation is necessary even when neutrophil cell counts in peripheral blood drop down to 500 cells/ml, and that neutrophil levels will shortly be restored back to the levels compatible with the scheduled chemotherapies.

Our studies demonstrated that this paradigm applies to treating cancer patients receiving a combination of cytostatic drugs cyclophosphamide, doxorubicin and fluorouracile (FAC regimen) and Panagen. In this regard, the following patient management scheme was adopted. If a patient displayed grade IV neutropenia on day 14 after the injection of cytostatics (second control point, when a maximum decrease in blood cellularity was observed), she was monitored daily and hourly and neutrophil cell counts in peripheral blood were measured. Such cases were unblinded and:

- if such patient received Panagen, neutrophil levels were monitored closely for the next 3-5 days, yet no leukostimulatory G-CSF-series medications were used. Should the patient develop any signs of oncoming “neutropenic fever” (increase in body temperature, mouth sores), she would immediately be given colony stimulating factors as well as appropriate antibiotics. However, we observed that in all cases of neutropenic patients who received Panagen, neutrophil count increased back to the normal level by the day 21 after the injection of cytostatics, and so the next course of chemotherapy would begin as scheduled.

- if such patient received placebo, she would immediately be started on G-CSF spectrum leukostimulatory medications (depending on the patients condition). In this case, the patient was no longer eligible to participate in the trial.

General protocol for blood sample collection

Blood samples for counting absolute numbers of leukocytes, neutrophils, monocytes and lymphocytes were collected 1-3 days before the start of the trial and on days 7 (± 1), 14 (± 2) and 21 (± 1) following each chemotherapy course. To monitor CD34+ cell counts, blood samples were drawn 1-3 days before the start of the trial, on day 7 (± 1) after the first course of chemotherapy and on day 21 (± 1) after each course of chemotherapy. Comprehensive metabolic panel was done 1-3 days before the trial and on day 21 (± 2) after the chemotherapy. If any of the chemotherapy courses was delayed, additional blood samples were taken 1-3 days before the next course of chemotherapy.

Safety monitoring (physical examination, vital signs) was conducted 1-3 days before each cycle of chemotherapy and on day 14 (± 2) following the chemotherapy. Patients were monitored for any adverse effects and were prescribed concomitant medications throughout the trial.

Chemotherapy was scheduled to repeat every 3 weeks. For the next chemotherapy round to begin, on day 1 of the next cycle (day 22 of the previous cycle) the patient should have $\geq 3.0\times10^9$ leucocytes/L, $\geq 1,5\times10^9$ neutrophils/L, and $\geq 100\times10^9$ platelets/L. Delay of up to one week in chemotherapy schedule was considered acceptable.

The number of patients enrolled in this clinical trial

		Novosibirsk Municipal Hospital No 1, FAC regimen	Novosibirsk Municipal Hospital No 1, AC regimen	Irkutsk Regional Oncology Dispensary, FAC regimen	Total
**Total number of patients enrolled in this clinical trial**		20	31	29	80
Panagen	On trial	14	19	18	51
	Excluded during the trial	1	1	4	6
Placebo	On trial	4	7	5	16
	Excluded during the trial	1	4	2	7

Basic principles of studying dynamics of hemopoietic parameters

Initially we attempted to directly compare absolute values for hemopoietic parameters. However, such comparison never highlighted significant differences between the groups because individual patients had strikingly scattered absolute values of the parameters measured. Nonetheless, some of the participating patients displayed positive dynamics of increasing parameter values throughout the CT courses. For these reasons, we also included comparative

analysis using the following approaches.

We compared relative values for parameters measured at different control timepoints relatively to the starting level. Such approach allowed estimates to be made of how well the medication can compensate the negative effects of cytostatics and to describe its supportive and restorative activity towards the hematopoietic system throughout the chemotherapy courses. We also contrasted the results from second and third courses of chemotherapy with those of the first round of chemotherapy, when hemopoietic parameters dropped the most. This analysis provided a relative estimate of protective and restorative activities of the medication on hemopoiesis regardless of the starting state of the patient's hematopoietic system.

Additionally, for every measured parameter we subgrouped the Panagen-group patients into responders and non-responders. Patients whose cell counts were higher at a given time point relative to the values observed at the starting time point or to the values measured after the first course of chemotherapy (set as 100%) were classified as responders. The rest of the patients were therefore non-responders, and displayed relative parameter values below 100%.

Extension protocol. Development of adaptive immune response

The following additional studies were performed as part of the phase II clinical trial:

1. Analysis of whether adaptive antitumor immunity can be induced in patients receiving combination therapy of cyclophosphamide, doxorubicin and Panagen. 17 patients receiving FAC CT (4 in placebo group, 13 in Panagen group) and 26 patients receiving AC therapy (7 in placebo group, 19 in Panagen group) participated in the study.

2. Analysis of whether pre-therapy levels of antitumor innate immunity cells can be maintained in patients receiving Panagen (17 FAC patients: 4 placebo and 13 Panagen-group).

3. Cytokine profiles were measured in patients enrolled into the study.

As part of and in parallel with the phase II clinical study presented here, we analyzed the development of adaptive immune response (presumably, anti-tumor immune response) in patients recruited to the study. The importance of studying this aspect was supported by vast experimental data from the past 12 years that strongly argued for the appearance of anticancer activity in experimental animals when various strategies involving exogenous human dsDNA were adopted. As it followed from these data, dendritic cells were the main target of such exogenous DNA in the context of anti-cancer activity. Dendritic cells stimulated Th-1 polarization, wherein a pool of specific CD8+ cytotoxic T-cells was formed.

Concomitant with the activation of anticancer adaptive immunity, current strategies for treating cancer patients with high-dose cytostatic therapies involve changes in cell numbers and functional states of T-regulatory cells (T-regs). Notably, this cell type is believed to suppress anti-

cancer immune response.

In light of these data, when analyzing the peripheral blood of patients in the study, three main goals were pursued – aiming at either clearly informing us on how Panagen affects the progression of adaptive immune response (presumably, anti-cancer), or showing which experiments are further needed.

We analyzed the following parameters describing activation of adaptive immunity (presumably, anti-cancer immunity) in grade II-IV breast cancer patients throughout the three courses of chemotherapy.

- relative and absolute cell counts of myeloid (CD11c+CD123–) and plasmacytoid (CD11c–CD123+) DCs in the peripheral blood of patients in course of cytoreductive therapy. This analysis provided details on the dynamics of DCs and their subpopulations throughout the therapy.
- relative and absolute numbers of T-regs (CD4+CD25+CD127–) showing suppresive activity.
- dynamics of the cytotoxic (CD8+ perforin+) T cells in peripheral blood, this cell type serving as a marker of mounting adaptive immune response (presumably, anti-cancer response).
- additional analysis of non-specific anticancer activity of PBMCs from patients enrolled in the study.

The extension protocol included stage II-IV breast cancer patients participating in the primary clinical trial evaluating the leukostimulatory activity of Panagen. The protocol for blood collection was identical to that of the primary protocol, and complied with the regulations from the Ministry of Healthcare and Social Development of the Russian Federation. To analyze the dynamics of adaptive immune response, 15 ml of blood was drawn in parallel to the blood collection for the primary protocol, and had no impact on patient health. PBMCs were isolated from blood samples, and this cell fraction was analyzed for specific markers and was subjected to specific tests. The following control time points were used throughout: 1-3 days before the first course of CT, 1 day before the second course of CT, and, upon completion of the therapy, on day 21 of the third course of CT. All therapeutic procedures were performed in the same way as was described for the primary protocol.

We additionally performed cytokine profiling of study participants and used the same blood collection schedule as for the primary protocol.

Assessment of patients' quality of life

Patients' quality of life was assessed and compared between the Panagen and placebo groups using the EORTC QLQ-C30 and QLQ-BR23 questionnaires, which were developed for cancer patients and specifically for breast cancer patients, respectively. These questionnaires incorporate two scales, symptom-related and functional. QLQ-C30 questionnaire also provides assessment of a patient's global health. Each scale ranges in score from 0 to 100. Higher values in functional and global health scales suggest improved quality of life, whereas the symptomatic scale works the other way around. Patients responded to these questionnaires before the study and after each CT course, i.e. on day 21 of CT courses.
