# Supplementary material for: Results of multicenter double-blind placebo-controlled phase II clinical trial of Panagen preparation to evaluate its leukostimulatory activity and formation of the adaptive immune response in patients with stage II-IV breast cancer
Source: BMC Cancer. 2015 Mar 13;15:122. doi: 10.1186/s12885-015-1142-z (PMC4365563; doi:10.1186/s12885-015-1142-z)
Supplement: Additional file 2: — Results of the completed phase II double-blind multicenter placebo-controlled clinical trial to evaluate the safety and leukostimulatory activity of Panagen in breast cancer patients. The study was performed at the Oncology Department of Novosibirsk Municipal Hospital No 1 and included 18 patients receiving FAC therapy (14 patients additionally received Panagen, 4 patients received placebo). [file 12885_2015_1142_MOESM2_ESM.pdf]

Additional file 2. Results of the completed phase II double-blind multicenter placebo-controlled clinical trial to evaluate the safety and leukostimulatory activity of Panagen in breast cancer patients. The study was performed at the Oncology Department of Novosibirsk Municipal Hospital No 1 and included 18 patients receiving FAC therapy (14 patients additionally received Panagen, 4 patients received placebo).

Twenty patients were enrolled in the study: 15 patients formed Panagen cohort, and 5 were in a placebo group. One patient from each group (Panagen and placebo) discontinued the therapy.

Analysis of leukostimulatory activity of Panagen. Dynamics of various types of WBCs in the peripheral blood of patients enrolled in the study.

Measurements of WBC counts in the control time points throughout the three cycles of chemotherapy

Enhanced leukocyte proliferation in response to Panagen was assayed by measuring and comparing the peripheral-blood WBC numbers in control time points in Panagen- vs placebo-group patients. Both absolute numbers and relative values were compared. This analysis was geared towards delineating the contribution of Panagen to stimulation of leukopoiesis during the three cycles of cytostatic chemotherapy (CT). It was important to determine how the proliferative potential of lymphoid progenitors is affected by increasingly negative influence of cytostatic therapy.

We explored the dynamics of WBC counts in the peripheral blood of patients in the control time points (**Table 1, Figure 1**). As it follows from our analysis, the most severe leucopenia is observed on day 14 after the injection of cytostatics across CT cycles **(Figure 1)**. In the control point on day 21 after the 3rd CT Panagen-group patients display significantly higher WBC count than placebo-group patients ($p<0.05$, Wilcoxon-Mann-Whitney test).

Table 1. WBC counts (×10^{9} cells/L) in patients from Panagen and placebo groups, as measured in the control time points throughout three cycles of CT.

	0	Days after the 1st CT			Days after the 2nd CT			Days after the 3rd CT		
		Day 7	Day 14	Day 21	Day 7	Day 14	Day 21	Day 7	Day 14	Day 21
**Panagen**										
02-01	7.2		3.6	3.2	9.7	4.4	7.0	6.5	2.7	4.9
02-02	6.5	3.1	2.1	5.0	3.6	5.1	11.1	3.4	2.2	5.0
02-03	8.2	4.0	2.0	6.1	3.8	2.7	7.6	4.7	3.5	6.3
02-04	7.7	5.1		10.3		1.5	6.0	0.9		
02-05	9.6	4.2	3.0	6.7	4.2	2.9	4.9	15.1	2.8	4.3
02-06	8.6	2.3	2.7	10.9	5.2	2.8	8.8	3.9	3.1	9.0
02-08	9.0	5.3	2.1	7.1	5.2	2.7	7.0	5.6	3.4	5.0
02-09	8.8	3.0	2.0	5.6	2.4	2.4	5.1	3.0	2.3	4.8
02-10	3.7	2.5	2.3	3.4	2.7	2.7	4.2	3.1		3.3
02-11	8.1	5.4	2.9	7.4	5.4	2.5	5.9		3.3	5.1
02-14	6.5	4.3	2.2	6.0		1.9	7.2	4.3	3.0	5.8
02-15		4.8	2.3	5.0		4.0	6.0	4.0	1.9	5.2
02-16		3.6	1.5	6.9	3.6	3.4	6.6	3.4	1.3	4.4
n	11	12	12	13	10	13	13	12	11	12
Median	**8.1**	**4.1**	**2.3**	**6.1**	**4.0**	**2.7**	**6.6**	**4.0**	**2.8**	**5.0**
Minimum	3.7	2.3	1.5	3.2	2.4	1.5	4.2	0.9	1.3	3.3

Maximum	9.6	5.4	3.6	10.9	9.7	5.1	11.1	15.1	3.5	9.0
Lower Quartile	6.5	3.1	2.1	5.0	3.6	2.5	5.9	3.3	2.2	4.6
Upper Quartile	8.8	5.0	2.8	7.1	5.2	3.4	7.2	5.2	3.3	5.5
**Placebo**										
02-07	7.6	4.6	2.2	8.1	3.5	2.1	5.0	4.1	2.4	4.3
02-12	6.7	5.0	3.6	4.4	4.3	3.2	4.6		1.9	3.6
02-13	6.2	5.2	2.2	5.0		2.4	6.2	5.1	2.4	3.9
02-17	8.2	3.2	1.2	2.4	3.4	2.9	3.6	4.0	1.8	4.4
n	4	4	4	4	3	4	4	3	4	4
Median	**7.2**	**4.8**	**2.2**	**4.7**	**3.5**	**2.7**	**4.8**	**4.1**	**2.2**	**4.1**
Minimum	6.2	3.2	1.2	2.4	3.4	2.1	3.6	4.0	1.8	3.6
Maximum	8.2	5.2	3.6	8.1	4.3	3.2	6.2	5.1	2.4	4.4
Lower Quartile	6.5	3.9	1.7	3.4	3.4	2.3	4.1	4.0	1.9	3.8
Upper Quartile	7.9	5.1	2.9	6.6	4.3	3.1	5.6	5.1	2.4	4.4

Footnote: Points where difference from the placebo group values is statistically significant are shown in red ($p<0.05$, Wilcoxon-Mann-Whitney test)

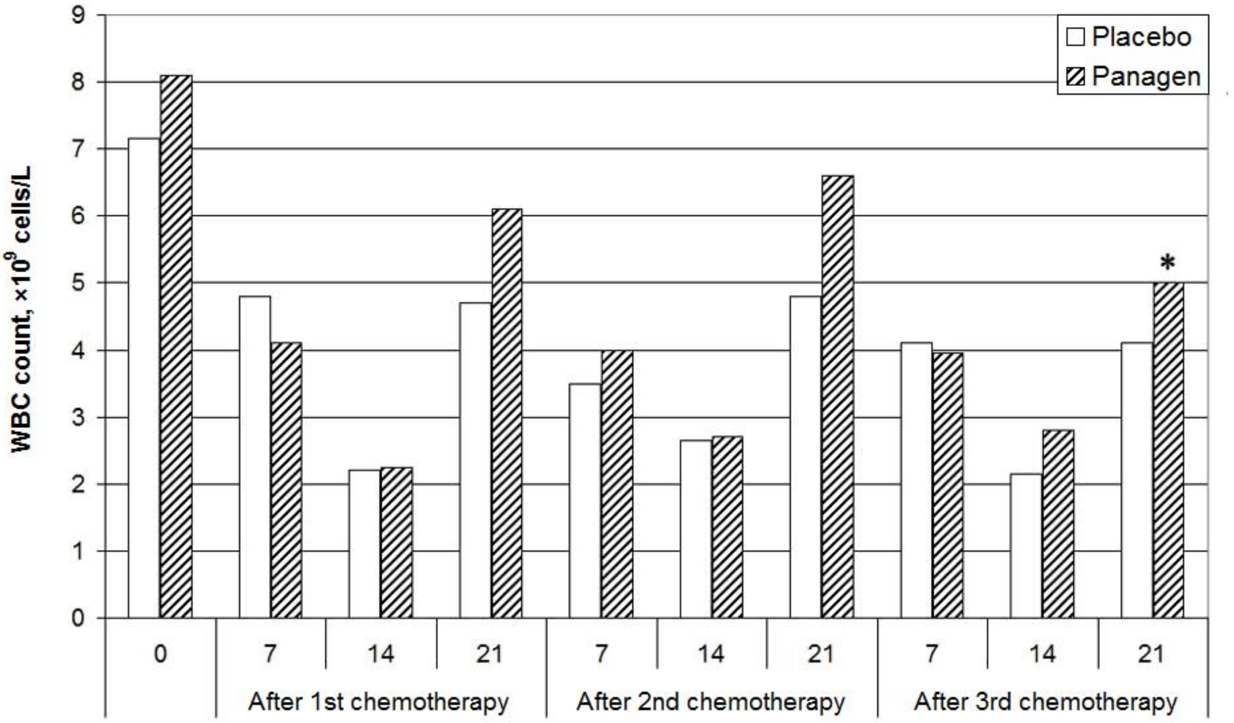

Figure 1. Absolute numbers of WBCs in the starting time point on day 0 and in the control points on days 7, 14 and 21 after three cycles of CT. Bars denote median values in groups. Asterisk (*) marks significant differences relatively to placebo group values ($p<0.05$, Wilcoxon-Mann-Whitney test).

Next, we analyzed WBC counts normalized to the values observed after the first CT (**Table 2, Figure 2**). The following analysis was performed for leukocyte counts (and subsequently for the neutrophils, monocytes and lymphocytes). Panagen-group patients, whose blood cell counts increased at a specific control point relatively to the level observed after the first CT (i.e. those with a relative content above 100%) were considered as responders. The rest of the patients were thus classified as non-responders (i.e. those with relative blood cell count below 100%)

Table 2. Relative leukocyte levels (expressed in %) normalized to the levels after the 1st CT in Panagen- and placebo cohorts on days 14 and 21 after the 2nd and 3rd CT.

	Day 14		Day 21	
	After the 2nd CT	After the 3rd CT	After the 2nd CT	After the 3rd CT
**Panagen**				
02-01	122.2	75.0	218.8	153.1
02-02	242.9	104.8	222.0	100.0

02-03	135.0	175.0	124.6	103.3
02-04			58.3	
02-05	96.7	93.3	73.1	64.2
02-06	103.7	114.8	80.7	82.6
02-08	128.6	161.9	98.6	70.4
02-09	120.0	115.0	91.1	85.7
02-10	117.4		123.5	97.1
02-11	86.2	113.8	79.7	68.9
02-14	86.4	136.4	120.0	96.7
02-15	173.9	82.6	120.0	104.0
02-16	226.7	86.7	95.7	63.8
Median	**121.1**	**113.8**	**98.6**	**91.2**
% Responders	75	64	46	33
% Non-responders	25	36	54	67
Median for responders	**128.6**	**115.0**	**124.1**	**103.6**
Median for non-responders	86.4	84.6	80.7	76.5
Total percentage of responding patients	**92**		**46**	
**Placebo**				
02-07	95.5	109.1	61.7	53.1
02-12	88.9	52.8	104.5	81.8
02-13	109.1	109.1	124.0	78.0
02-17	241.7	150.0	150.0	183.3
Median	**102.3**	**109.1**	**114.3**	**79.9**

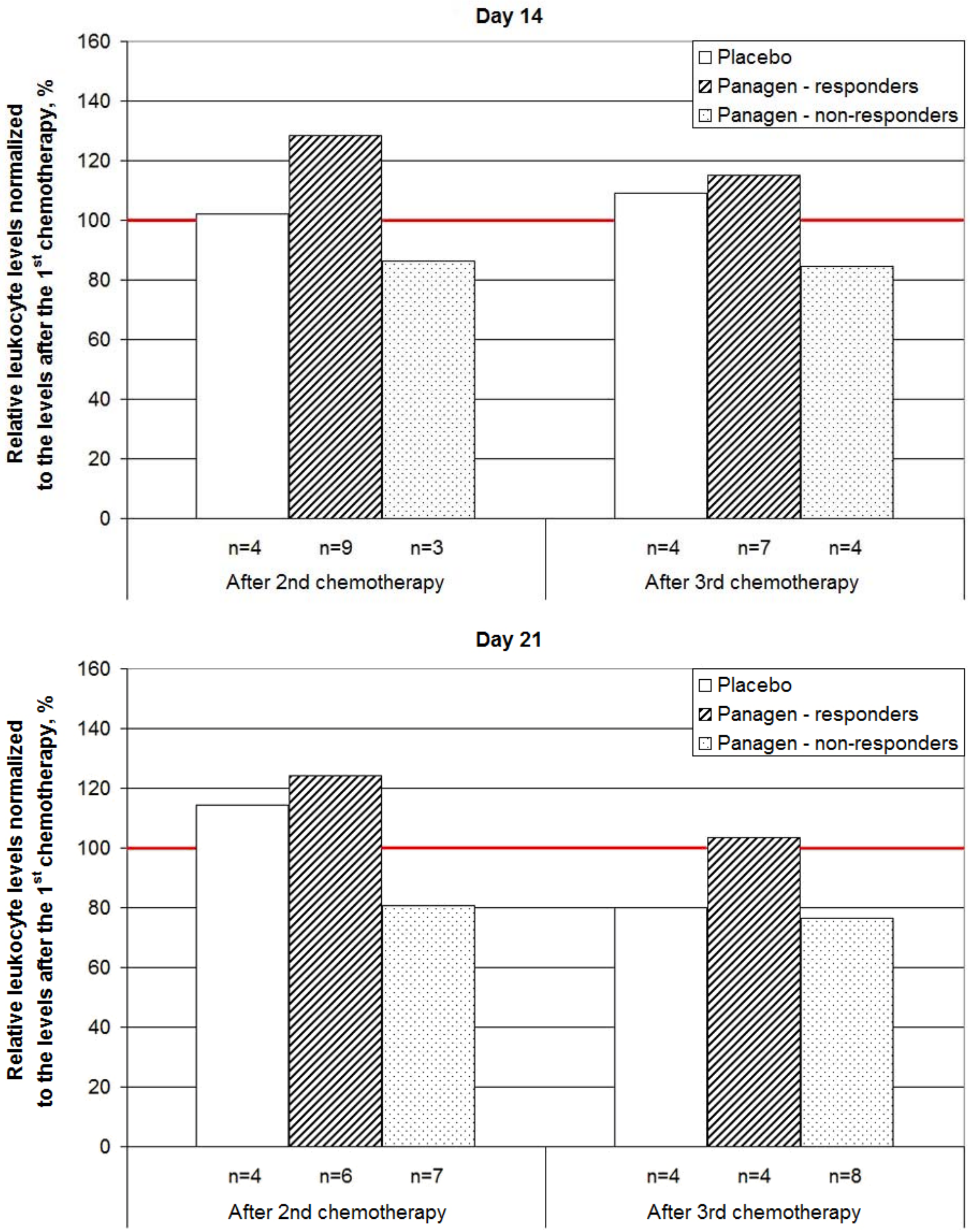

Figure 2. Relative levels of peripheral-blood leukocytes in patients on days 14 and 21 after the 2nd and 3rd CT, normalized to the level observed after the 1st CT (set to 100%, red line).

As we were analyzing the results, we ran into an interesting observation. Several patients classified as Panagen responders and non-responders in two consecutive control points (days 14 and 21 after CT) switch places and move to an opposite group. Specifically, those patients who responded by increasing peripheral-blood leukocyte counts on day 14 after injection of cytostatics, displayed leukocyte counts close to or even below the starting values (set to 100%)

by day 21. Conversely, the non-responding patients (as of the day 14), showed positive Panagen response by day 21. We speculate that proliferative capacity of committed unipotent lymphoid progenitors is enhanced by Panagen at temporally distinct “windows of receptivity”, which results in shifting the onset of proliferation and is mirrored by the dynamics of leukocytes in the patients peripheral blood samples.

Measuring neutrophil counts in the control time points across the three cycles of CT

We measured neutrophil count in patient peripheral blood samples across all control time points throughout CT **(Table 3, Figure 3).** Our analysis demonstrates, that in the control time point on day 21 after the second CT, the neutrophil counts in Panagen-group patients are significantly different from those of placebo-group patients.

Table 3. Neutrophil count (×10^9 cells/L) in patients from Panagen and placebo cohorts, measured in control time points after three cycles of CT.

	0	Days after the 1st CT			Days after the 2nd CT			Days after the 3rd CT		
		Day 7	Day 14	Day 21	Day 7	Day 14	Day 21	Day 7	Day 14	Day 21
**Panagen**										
02-01	5.40		1.51	2.24	8.05	2.68	5.18	5.46	1.78	0.64
02-02	4.23	2.05	1.51	3.65	2.56	2.81	7.88	2.45	1.28	3.30
02-03	5.33	2.96	1.48	3.97	2.58	0.59	4.79	3.24	1.47	4.35
02-04	6.08	4.13		5.97		0.27	4.62	0.40		
02-05	6.43	2.73	0.99	3.62	2.48	1.07	2.70	11.02	1.06	1.16
02-06	6.36	1.73	1.59	7.63	3.22	1.40	6.42	2.46	1.30	5.58
02-08	6.84	4.03	0.86	4.76	3.33	1.54	3.43	3.92	1.77	1.50
02-09	5.28	1.71	0.40	3.30	1.37	1.13	2.70	1.71	1.43	3.07
02-10	2.33	1.50	1.27	1.87	1.70	1.43	2.98	1.77		2.11
02-11	4.94	3.46	1.22	5.25	4.10	1.70	4.48		2.11	3.57
02-14	4.42	2.54	1.25	3.30		0.80	4.82	3.48	0.75	3.60
02-15		3.36	1.31	2.90		2.56	4.50		0.49	3.48
02-16		2.59		3.93	2.66	1.84	4.82	2.31	0.34	2.90
n	11	12	11	13	10	13	13	11	11	12
Median	**5.33**	**2.66**	**1.27**	**3.65**	**2.62**	**1.43**	**4.62**	**2.46**	**1.30**	**3.19**
Minimum	2.33	1.50	0.40	1.87	1.37	0.27	2.70	0.40	0.34	0.64
Maximum	6.84	4.13	1.59	7.63	8.05	2.81	7.88	11.02	2.11	5.58

Lower Quartile	4.42	1.89	0.99	3.30	2.48	1.07	3.43	1.77	0.75	1.81
Upper Quartile	6.36	3.41	1.51	4.76	3.33	1.84	4.82	3.92	1.77	3.58
**Placebo**										
02-07	5.78	3.45	0.90	5.18	2.07	1.18	2.75	2.50	1.30	2.45
02-12	4.62	3.30	2.20	2.33	2.71	1.98	2.76		1.06	2.20
02-13	3.78	3.43	1.50	3.25		1.34	3.78	3.52	1.34	2.81
02-17	6.31	2.37	0.43	1.03	2.65	1.28	1.69	3.36		2.42
n	4	4	4	4	3	4	4	3	3	4
Median	**5.20**	**3.37**	**1.20**	**2.79**	**2.65**	**1.31**	**2.76**	**3.36**	**1.30**	**2.44**
Minimum	3.78	2.37	0.43	1.03	2.07	1.18	1.69	2.50	1.06	2.20
Maximum	6.31	3.45	2.20	5.18	2.71	1.98	3.78	3.52	1.34	2.81
Lower Quartile	4.20	2.83	0.67	1.68	2.07	1.23	2.22	2.50	1.06	2.31
Upper Quartile	6.05	3.44	1.85	4.22	2.71	1.66	3.27	3.52	1.34	2.63

Footnote: Points where difference from the placebo group values is statistically significant are shown in red ($p<0.05$, Wilcoxon-Mann-Whitney test)

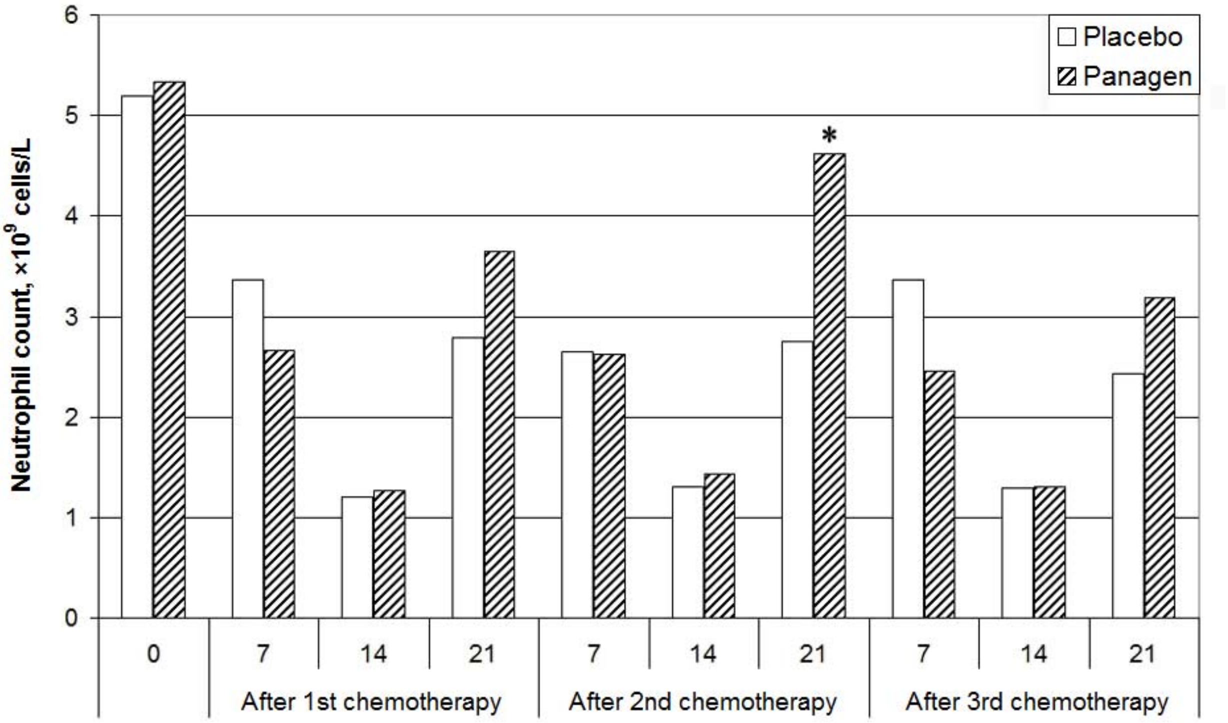

Figure 3. Neutrophil count in the starting time point on day 0 and in the control points on days 7, 14 and 21 after three cycles of CT. Bars denote median values for each group. Asterisk (*) marks significant differences relatively to placebo group values ($p<0.05$, Wilcoxon-Mann-Whitney test).

Next, we proceeded to analyze the relative levels of neutrophils as compared to the values obtained after the first CT **(Table 4, Figure 4)**. This analysis may help uncover the stimulatory contribution of Panagen on production of neutrophils that is known to be attenuated as the patients progress through the cytostatic CT cycles. We set out to explore how the potential of neutrophil progenitors is affected throughout the increasingly detrimental cytostatic therapy.

Table 4. Relative percentage of neutrophils (%) normalized to the level observed after the 1st CT in Panagen and placebo cohorts on days 14 and 21 after the 2nd and 3rd CT. Significant differences of Panagen-responders subgroup vs placebo patients are marked by blue and an asterisk (*) ($p<0.1$, Wilcoxon-Mann-Whitney test).

	Day 14		Day 21	
	After the 2nd CT	After the 3rd CT	After the 2nd CT	After the 3rd CT
**Panagen**				
02-01	177.5	117.9	231.3	28.4
02-02	185.5	84.4	215.9	90.4
02-03	40.1	99.3	120.8	109.6
02-04			77.3	

02-05	108.4	107.5	74.5	32.1
02-06	87.9	81.7	84.2	73.1
02-08	178.7	205.3	72.1	31.5
02-09	282.0	356.5	81.8	93.0
02-10	113.1		159.5	112.9
02-11	139.6	173.4	85.3	67.9
02-14	63.6	59.8	146.2	109.0
02-15	195.3	37.7	155.2	120.1
02-16			122.5	73.8
Median	**139.6**	**103.4**	**120.8**	**82.1**
Percent responders, %	73	50	54	33
Percent non-responders, %	27	50	46	67
Median value for responders	**178.1**	**173.4**	**155.2***	**111.3**
Median value for non-responders	63.6	81.7	79.6	70.5
Combined percentage of responders, %	**73**		**54**	
**Placebo**				
02-07	130.4	143.7	53.0	47.3
02-12	90.3	48.5	118.4	94.2
02-13	89.8	89.8	116.4	86.4
02-17	295.4		164.0	234.5
Median	**110.4**	**89.8**	**117.4**	**90.3**

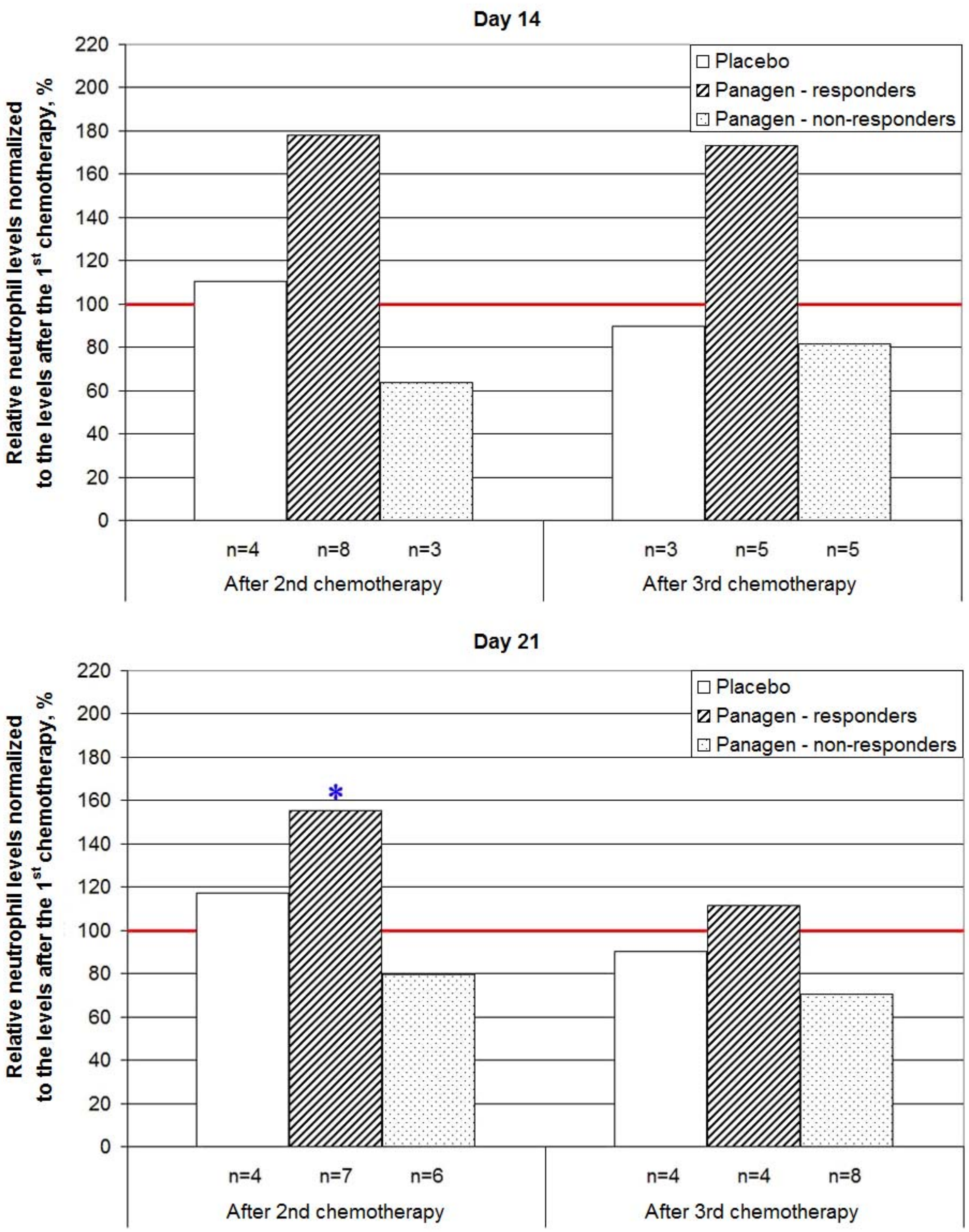

Figure 4. Relative neutrophil levels in peripheral blood of patients on days 14 and 21 after the 2nd and 3rd rounds of CT normalized to the level after the 1st CT (set to 100%, shown as red line). Blue asterisk denotes significant difference from the placebo group values ($p<0.1$, Wilcoxon-Mann-Whitney test).

This analysis demonstrated that Panagen enhances neutrophil production, and this enhancement reaches statistical significance in the control time point on day 21 after the second CT.

As we were analyzing the results, we were puzzled by the following fact. Several patients classified as Panagen responders and non-responders in two consecutive control points (for instance, on days 14 and 21 after CT) switch places and move to an opposite group. Specifically, those patients who responded with higher neutrophil count on day 14 after injection of cytostatics, displayed neutrophil values close to or even below the starting values (set to 100%) by day 21. Conversely, the non-responding patients (as of the day 14), showed positive Panagen response by day 21. We speculate that proliferative capacity of committed unipotent neutrophil progenitors is enhanced by Panagen at temporally distinct "windows of receptivity", which results in shifting the onset of neutrophil production and is mirrored by the dynamics of neutrophils in the patient peripheral blood samples.

We established that the occurrence of neutropenia peaked on day 14 after the injections of cytostatics **(Table 5, Figure 5),** reaching the value of 1 in the placebo cohort, and 0.7 in the Panagen cohort**.**

Table 5. Occurrence of neutropenia in Panagen and placebo cohorts, as assayed in the control time points throughout the three cycles of CT.

	Neutro-penia grade	0	Day 7			Day 14			Day 21		
			After the 1st CT	After the 2nd CT	After the 3rd CT	After the 1st CT	After the 2nd CT	After the 3rd CT	After the 1st CT	After the 2nd CT	After the 3rd CT
Panagen	I	0	0.25	0.10	0.18	0.27	0.23	0.18	0.08	0	0.08
	II	0	0	0.10	0	0.45	0.31	0.45	0	0	0.08
	III	0	0	0	0	0.18	0.15	0.09	0	0	0.08
	IV	0	0	0	0.09	0.09	0.08	0.18	0	0	0
Placebo	I	0	0	0	0	0	0.25	0	0	0.25	0
	II	0	0	0	0	0.25	0.75	1.00	0.25	0	0
	III	0	0	0	0	0.25	0	0	0	0	0
	IV	0	0	0	0	0.25	0	0	0	0	0

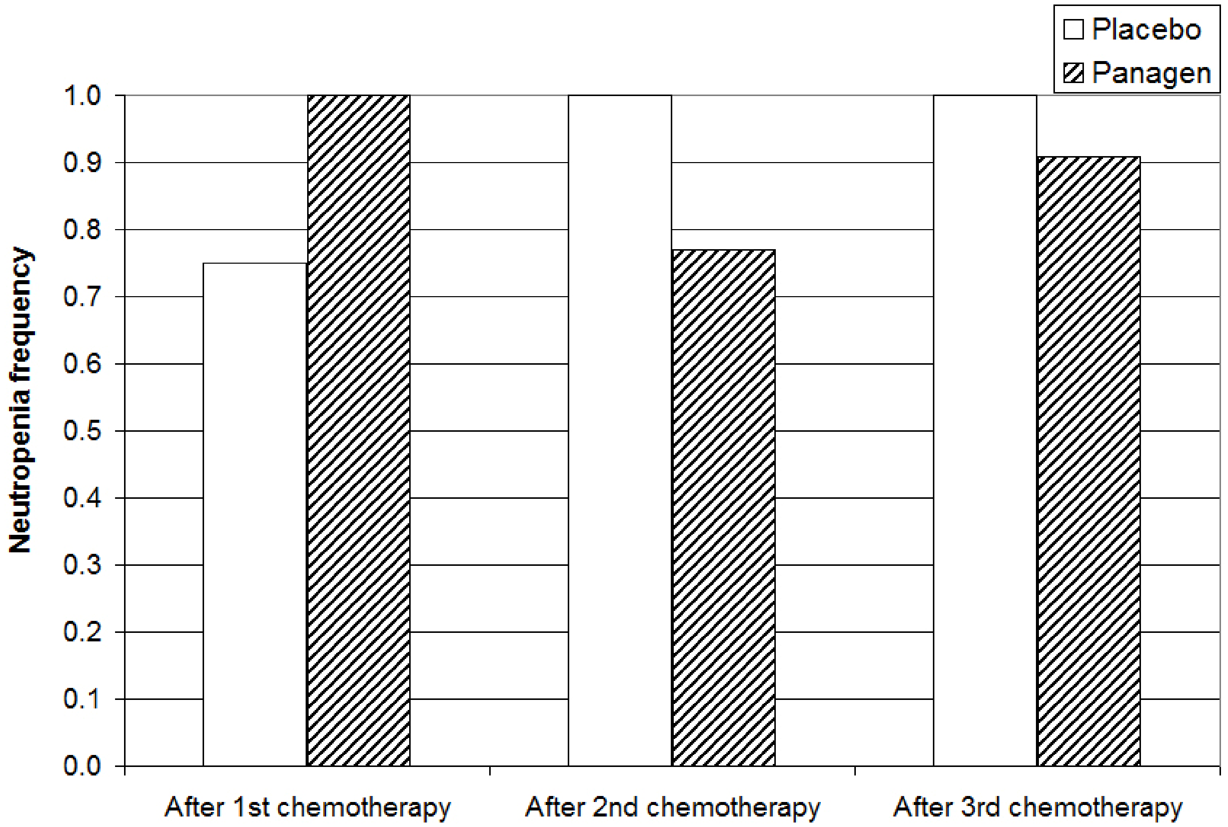

Figure 5. Occurrence of grade I-IV neutropenia in patients on day 14 after injection of cytostatics across three CT cycles.

Moreover, we found out that neutrophil counts restored quicker after reaching their minimum levels in Panagen patients as compared to placebo patients, which is very important, because this dynamics allows for neutrophil counts to go back to the levels compatible with the next CT cycle. This observation lies at the core of the paradigm of controlled grade IV neutropenia, whereby no G-CSF leukostimulatory medications are needed to restore peripheral-blood neutrophil counts. Instead, Panagen induces proliferation in the neutrophil lineage so that the neutrophil counts become normal by the 21 day after the CT **(Figure 6).**

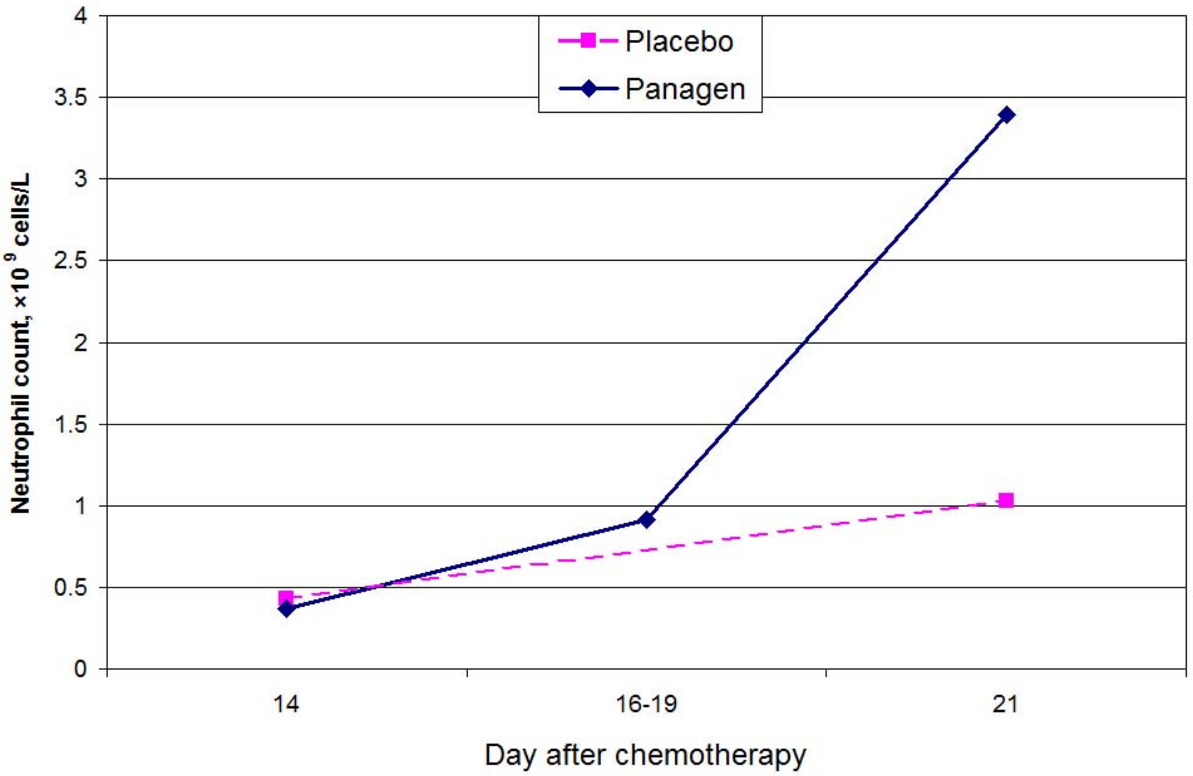

Figure 6. In the absence of additional leukostimulation, neutrophil population in peripheral blood is restored from its minimum level (febrile neutropenia level) faster in Panagen patients vs placebo patients.

Analysis of monocyte counts in the control time points throughout three CT cycles

Our study of how Panagen affects the dynamics of monocyte lineage was essentially similar to the analysis of leukocyte and neutrophil dynamics. We compared placebo and Panagen groups for absolute monocyte counts in the peripheral blood samples of study participants (**Table 6, Figure 7**).

Table 6. Monocyte counts (×10^9 cells/L) in patients from Panagen and placebo cohorts, measured in control time points throughout three CT cycles.

	0	Days after the 1st CT			Days after the 2nd CT			Days after the 3rd CT		
		Day 7	Day 14	Day 21	Day 7	Day 14	Day 21	Day 7	Day 14	Day 21
**Panagen**										
02-01	0.360		0.504	0.064	0.097	0.220	0.280	0.130	0.054	0.539
02-02	0.260	0.124	0.021	0.200	0.072	0.102	0.666	0.068	0.176	0.400
02-03	0.164	0.160	0.060	0.122	0.114	0.054	0.456	0.094	0.385	0.567
02-04	0.154	0.102		0.412		0.105	0.360			
02-05	0.288	0.084	0.060	0.335	0.252	0.290	0.441	0.755	0.308	0.559
02-06	0.430	0.046	0.135	0.327	0.156	0.112	0.264	0.156	0.310	0.900
02-08	0.180	0.159	0.105	0.426	0.312	0.189	0.630	0.224	0.306	0.950
02-09	0.264	0.060	0.040	0.504	0.144	0.120	0.459	0.240	0.023	0.096
02-10	0.111	0.075	0.207	0.374	0.189	0.162	0.084	0.093		0.033
02-11	0.567	0.594	0.145	0.074	0.054	0.050	0.177		0.099	0.102
02-14	0.065	0.017	0.022	0.600		0.190	0.216	0.043	0.030	0.232
02-15		0.048	0.046	0.100		0.080	0.060		0.019	0.624
02-16		0.036		0.414	0.036	0.136	0.132	0.068	0.156	0.264
n	11	12	11	13	10	13	13	10	11	12
Median	**0.260**	**0.080**	**0.060**	**0.335**	**0.129**	**0.120**	**0.280**	**0.112**	**0.156**	**0.470**
Minimum	0.065	0.017	0.021	0.064	0.036	0.050	0.060	0.043	0.019	0.033

Maximum	0.567	0.594	0.504	0.600	0.312	0.290	0.666	0.755	0.385	0.950
Lower Quartile	0.154	0.047	0.040	0.122	0.072	0.102	0.177	0.068	0.030	0.167
Upper Quartile	0.360	0.142	0.145	0.414	0.189	0.189	0.456	0.224	0.308	0.596
**Placebo**										
02-07	0.152	0.092	0.110	0.405	0.245	0.168	0.200	0.246	0.240	0.473
02-12	0.536	0.400	0.288	0.044	0.043	0.096	0.184		0.076	0.144
02-13	0.062	0.052	0.022	0.250		0.024	0.062	0.051	0.024	0.273
02-17	0.328	0.032	0.024	0.072	0.068	0.261	0.324	0.040		0.308
n	4	4	4	4	3	4	4	3	3	4
Median	**0.240**	**0.072**	**0.067**	**0.161**	**0.068**	**0.132**	**0.192**	**0.051**	**0.076**	**0.291**
Minimum	0.062	0.032	0.022	0.044	0.043	0.024	0.062	0.040	0.024	0.144
Maximum	0.536	0.400	0.288	0.405	0.245	0.261	0.324	0.246	0.240	0.473
Lower Quartile	0.107	0.042	0.023	0.058	0.043	0.060	0.123	0.040	0.024	0.209
Upper Quartile	0.432	0.246	0.199	0.328	0.245	0.215	0.262	0.246	0.240	0.391

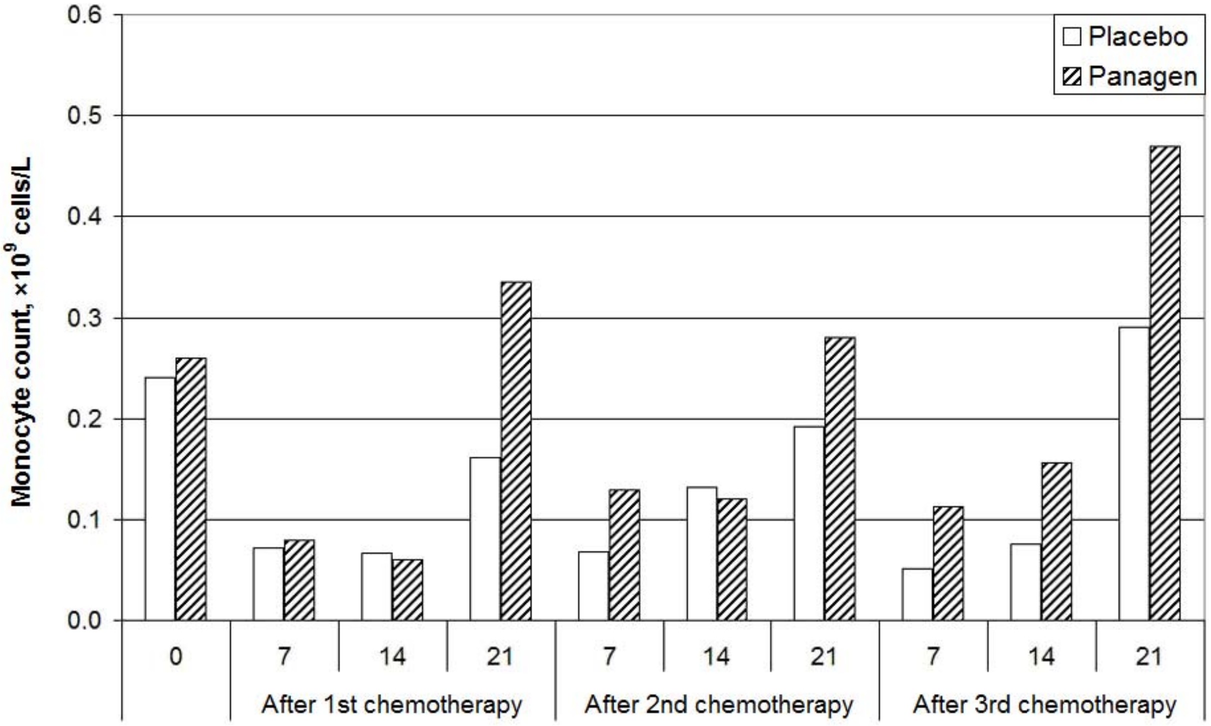

Figure 7. Absolute monocyte counts measured in the starting time point (day 0) and in the control points on days 7, 14 and 21 after three CT cycles. Median values for Panagen and placebo groups are shown.

This analysis failed to uncover statistically significant differences between Panagen and placebo cohorts.

Similarly to the previous analyses, we also estimated relative monocyte abundance in each patient group in control time points 14 and 21 days after injection of cytostatics during the second and third CT cycles compared to the levels after the first CT. In every control time point, the patients were grouped into Panagen-responders and non-responders (**Table 7, Figure 8**). Our analysis demonstrates that Panagen has a pronounced impact on monocyte levels in peripheral blood and allows Panagen-responders to maintain high monocyte counts on day 14 after the CT cycles.

Table 7. Relative monocyte levels (%) in peripheral blood of patients on days 14 and 21 after the 2nd and 3rd CT normalized to the level after the 1st CT (set to 100%, shown as red line). Red asterisk denotes significant difference from the placebo group values (p<0.1, Wilcoxon-Mann-Whitney test).

	Day 14		Day 21	
	After the 2nd CT	After the 3rd CT	After the 2nd CT	After the 3rd CT
**Panagen**				

02-01	43.7	10.7	437.5	842.2
02-02	485.7	838.1	333.0	200.0
02-03	90.0	641.7	373.8	464.8
02-04			87.4	
02-05	483.3	513.3	131.6	166.9
02-06	83.0	229.6	80.7	275.2
02-08	180.0	291.4	147.9	223.0
02-09	300.0	57.5	91.1	19.0
02-10	78.3		22.5	8.8
02-11	34.5	68.3	239.2	137.8
02-14	863.6	136.4	36.0	38.7
02-15	173.9	41.3	60.0	624.0
02-16			31.9	63.8
Median	**173.9**	**183.0**	**91.1**	**183.4**
Percent responders, %	55	60	46	67
Percent non-responders, %	45	40	54	33
Median value for responders	**391.7**	**402.4***	**286.1**	**249.1**
Median value for non-responders	78.3	49.4	60.0	28.9
All responding patients combined, %	**73**		**62**	
**Placebo**				
02-07	152.7	218.2	49.4	116.8
02-12	33.3	26.4	418.2	327.3
02-13	109.1	109.1	24.8	109.2
02-17	1087.5		450.0	427.8
Median	**130.9**	**109.1**	**233.8**	**222.0**

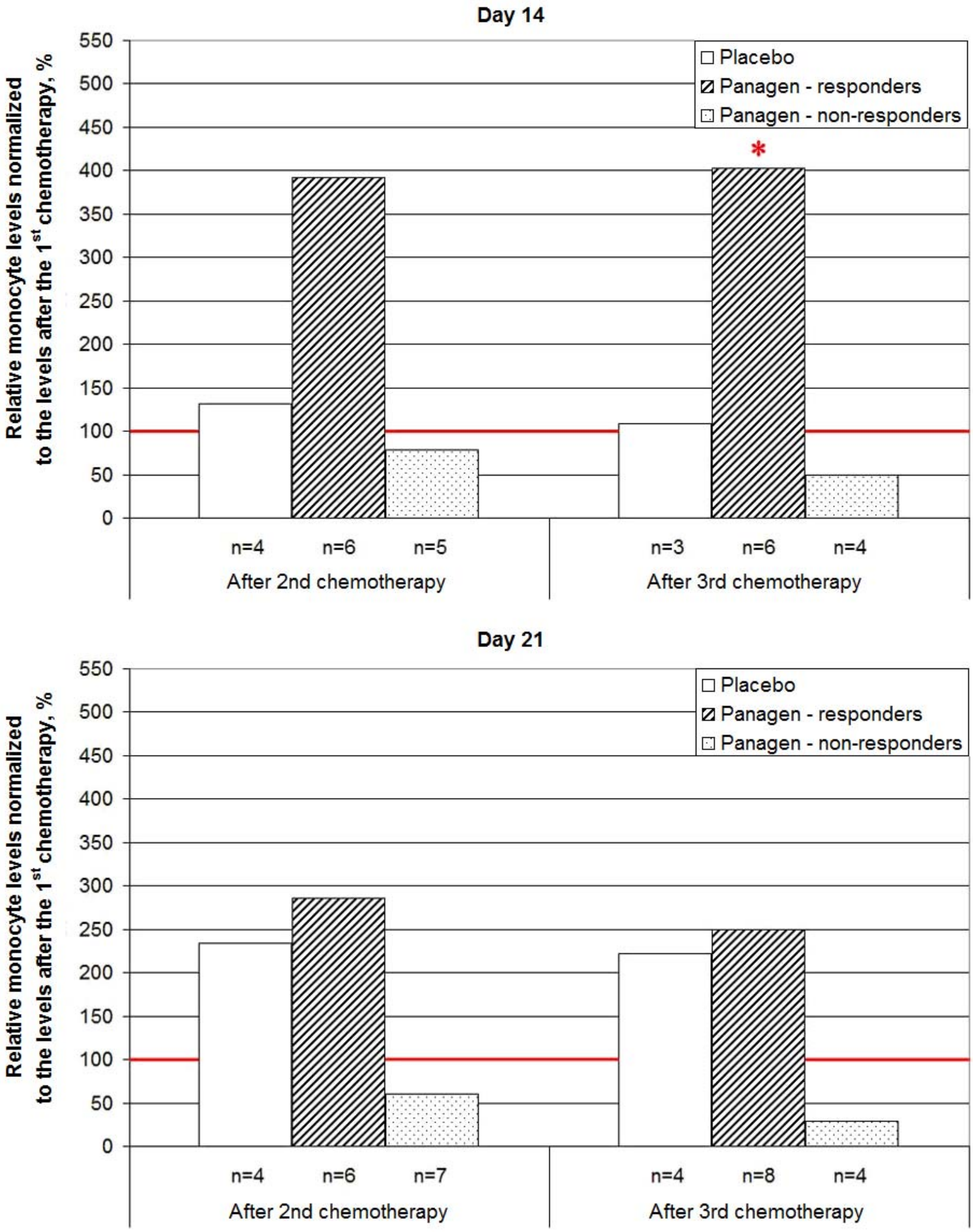

Figure 8. Relative monocyte levels in peripheral blood of patients on day 14 and 21 after the 2nd and 3rd CT normalized to monocyte counts after the 1st CT (set to 100%, red line). Red asterisk denotes statistically significant difference from a placebo group ($p<0.05$, Wilcoxon-Mann-Whitney test).

Dynamic changes in peripheral lymphocyte pool in study participants

Much like peripheral mononuclear fraction, lymphocyte fraction serves as an important marker of immune competency of a cancer patient. Mononuclear cell fraction gives rise to

antigen-presenting cells, macrophages and DCs. Lymphocyte fraction includes the major cellular effectors of immune system, T- and B-cells.

In our study, we evaluated the total lymphocyte pool throughout the CT cycles (**Table 8, Figure 9**).

Table 8. Lymphocyte counts ($\times 10^9$ cells/L) in Panagen- and placebo-group patients measured in different control time points throughout 3 cycles of CT

	0	Days after the 1st CT			Days after the 2nd CT			Days after the 3rd CT		
		Day 7	Day 14	Day 21	Day 7	Day 14	Day 21	Day 7	Day 14	Day 21
**Panagen**										
02-01	1.44		0.90	0.90	1.55	1.45	1.47	0.85	0.81	1.27
02-02	2.02	0.93	0.55	1.10	0.97	2.19	2.33	0.85	0.73	1.25
02-03	2.54	0.88	0.46	1.95	1.03	1.49	2.28	1.32	1.61	1.39
02-04	2.23	0.87		3.91		1.10	0.90	0.43		
02-05	2.78	1.34	1.92	2.55	1.47	1.54	1.72	3.17	1.43	2.49
02-06	1.12	0.53	0.97	2.83	1.77	1.26	1.94	1.29	1.49	2.43
02-08	1.80	1.01	1.11	1.85	1.56	0.92	2.94	1.29	1.29	2.35
02-09	3.17	1.20	0.78	1.79	0.84	1.15	1.94	1.05	0.81	1.58
02-10	1.22	0.85	0.78	1.16	0.81	1.11	0.92	1.02		1.02
02-11	2.51	1.30	1.54	2.07	1.24	0.75	1.24		1.02	1.38
02-14	1.95	1.72	0.92	2.10		0.46	2.09	0.60	2.04	1.86
02-15		1.39	0.94	2.00		1.32	1.56		0.44	1.09
02-16		0.76		2.48	0.90	1.43	1.65	1.02	0.78	1.23
n	11	12	11	13	10	13	13	11	11	12
Median	**2.02**	**0.97**	**0.92**	**2.00**	**1.13**	**1.26**	**1.72**	**1.02**	**1.02**	**1.38**
Minimum	1.12	0.53	0.46	0.90	0.81	0.46	0.90	0.43	0.44	1.02

Maximum	3.17	1.72	1.92	3.91	1.77	2.19	2.94	3.17	2.04	2.49
Lower Quartile	1.44	0.86	0.78	1.79	0.90	1.10	1.47	0.85	0.78	1.24
Upper Quartile	2.54	1.32	1.11	2.48	1.55	1.45	2.09	1.29	1.49	2.10
**Placebo**										
02-07	1.60	1.01	1.17	2.43	1.19	0.76	2.00	1.35	0.86	1.29
02-12	1.54	1.30	1.08	2.16	1.55	1.12	1.66		0.76	1.22
02-13	2.23	1.72	0.68	1.50		1.03	2.23	1.53	0.98	0.82
02-17	1.56	0.77	0.74	1.30	0.65	1.28	1.51	0.60		1.67
n	4	4	4	4	3	4	4	3	3	4
Median	**1.58**	**1.16**	**0.91**	**1.83**	**1.19**	**1.08**	**1.83**	**1.35**	**0.86**	**1.26**
Minimum	1.54	0.77	0.68	1.30	0.65	0.76	1.51	0.60	0.76	0.82
Maximum	2.23	1.72	1.17	2.43	1.55	1.28	2.23	1.53	0.98	1.67
Lower Quartile	1.55	0.89	0.71	1.40	0.65	0.89	1.58	0.60	0.76	1.02
Upper Quartile	1.91	1.51	1.12	2.29	1.55	1.20	2.12	1.53	0.98	1.48

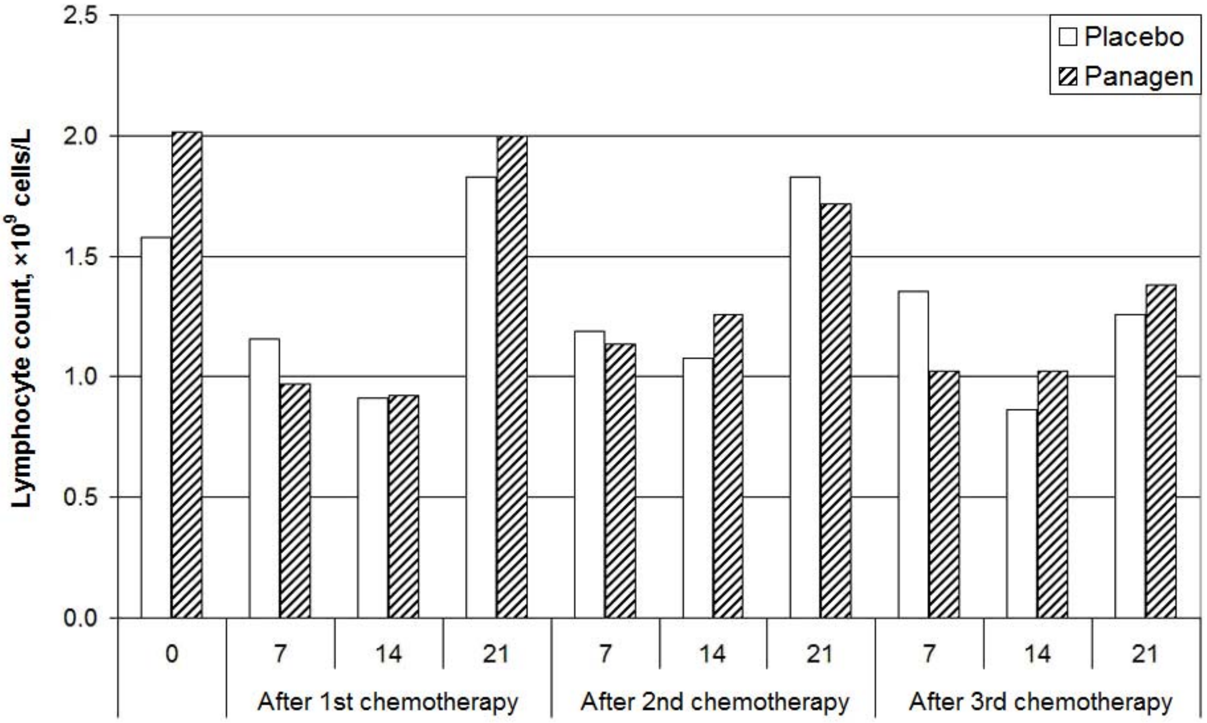

Figure 9. Absolute lymphocyte counts measured in starting point on day 0 and in control time points on days 7, 14 and 21 after 1, 2, and 3 rounds of CT. Median values in each group are shown.

Direct comparison of absolute lymphocyte counts failed to uncover significant differences between Panagen and placebo groups. Estimates of relative lymphocyte levels were performed as described above (**Table 9, Figure 10)**.

Our results show that if one considers the entire sampling, the effects of Panagen on lymphoid lineage are close to negligible, even though lymphocyte counts in Panagen patients on days 14 and 21 after the second and third CTs are reproducibly higher than those measured in the placebo group. Nonetheless, comparison of Panagen-responders vs placebo shows clear differences (**Table 9, Figure 10).** Panagen helps maintain the functionality of lymphopoietic lineage at significantly higher levels (130-160%) than what has been observed after the first CT cycle.

Table 9. Relative abundance of lymphocytes (%) normalized to the levels after the 1st CT in Panagen- and placebo-group patients on days 14 and 21 after the 2nd and 3rd CT. Statistically significant differences in Panagen-responders vs placebo groups of patients are shown in blue and are marked with an asterisk (*)(p<0.1, Wilcoxon-Mann-Whitney test).

	Day 14		Day 21	
	After the 2nd CT	After the 3rd CT	After the 2nd CT	After the 3rd CT

**Panagen**				
02-01	161.3	90.0	164.1	142.2
02-02	401.6	133.0	211.9	113.6
02-03	322.8	350.0	116.8	71.0
02-04			23.0	
02-05	80.1	74.4	67.4	98.0
02-06	129.6	153.1	68.3	85.7
02-08	82.5	116.1	159.3	127.3
02-09	147.7	103.2	108.1	88.4
02-10	141.6		79.9	88.5
02-11	48.8	66.6	59.8	66.5
02-14	49.4	220.8	99.4	88.4
02-15	140.0	46.3	78.0	54.6
02-16			66.4	49.6
Median	**140.0**	**109.6**	**79.9**	**88.4**
Percentage of responding patients	64	60	38	25
Percentage of non-responding patients	36	40	62	75
Median value for responders	**147.7**	**143.0**	**159.3***	**127.3**
Median value for non-responders	64.7	70.5	67.8	85.7
Combined percentage of responding patients, %	**82**		**38**	
**Placebo**				
02-07	64.8	74.1	82.3	53.1
02-12	103.7	70.4	76.8	56.8
02-13	151.3	144.3	148.8	54.6
02-17	171.5		116.7	129.0
Median	**127.5**	**74.1**	**99.5**	**55.7**

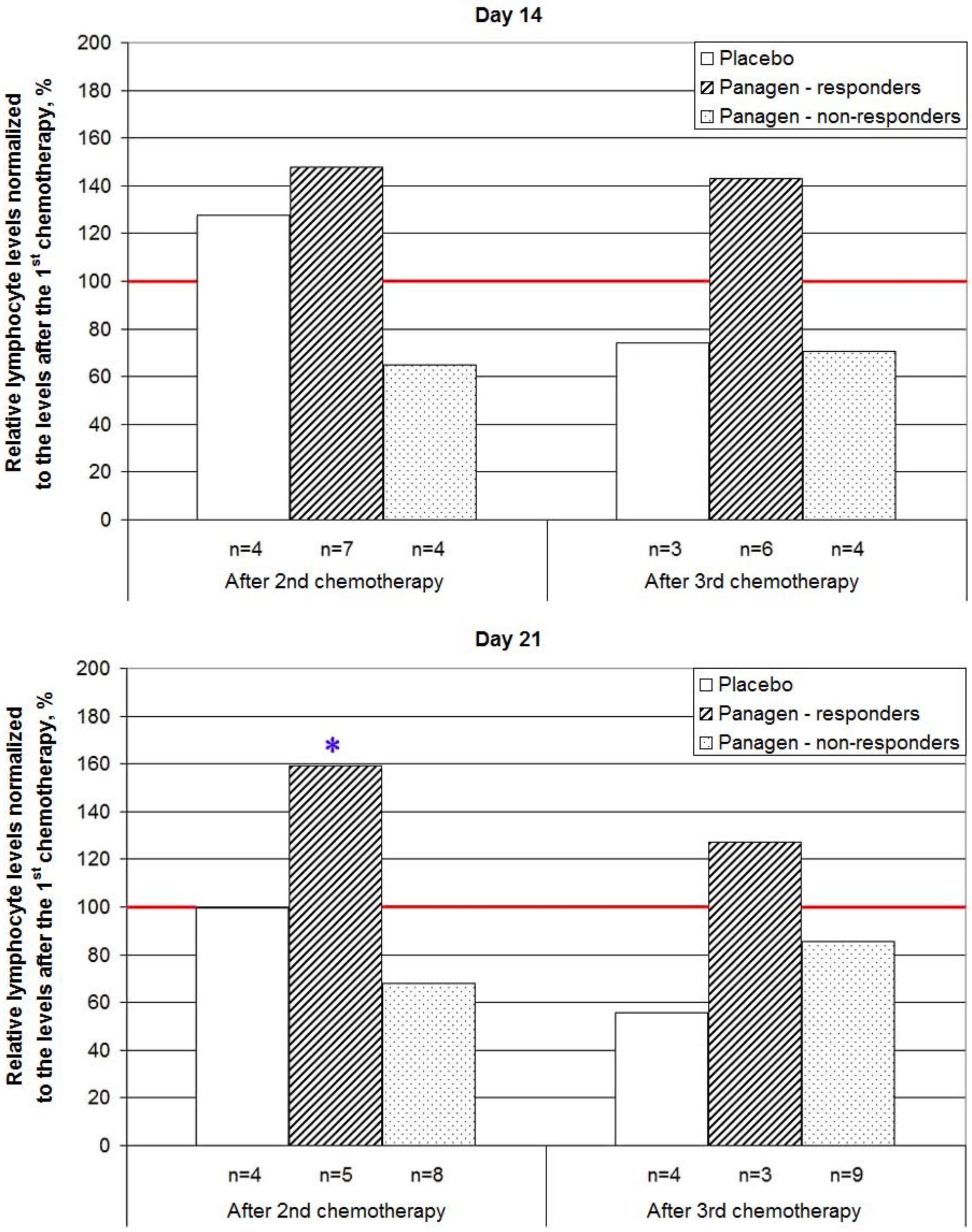

Figure 10. Relative lymphocyte counts in patients on days 14 and 21 after the 2nd and 3rd CT normalized to the levels found after the 1st CT (set to 100%, marked with red line). Blue asterisk denotes the values that are significantly different from the placebo group ($p<0.1$, Wilcoxon-Mann-Whitney test).

Panagen activity toward erythropoietic lineage. Erythropoiesis, effects on megakaryocyte lineage.

Panagen was evaluated for influencing erythropoietic lineage throughout CT cycles (**Tables 10-15**). As it follows from our analysis, Panagen is moderately active toward erythropoietic lineage.

On day 14 following the CTs, much like it was observed for other blood cell types, RBC counts went down (**Table 10**).

Lower haemoglobin levels were reported for the patients in the trial, totaling 63% patients in Panagen group and 100% in placebo group (**Table 11**). An increase in platelets was observed by the day 21 after CT rounds in 23% Panagen patients (**Table 12**). Majority of the patients also displayed higher ESR (**Table 13**).

No changes in mean cell haemoglobin concentration (MCHC) were observed – only 15% of Panagen-group patients had lower MCHC, which was likely due to individual differences (**Table 14**).

All patients from both Panagen and placebo groups had low hematocrit (Hct) values in most control time points (**Table 15**).

Table 10. RBC counts in blood samples of patients from Panagen and placebo groups in different control time points after the CT. Normal range is 3.8 - 5.1 $\times 10^{12}$ cells/L.

	0	Days after the 1st CT			Days after the 2nd CT			Days after the 3rd CT		
		7	14	21	7	14	21	7	14	21
**Panagen**										
02-01	4.72		4.24	4.21	4.15	4.41	4.68	4.23	4.11	4.02
02-02	3.18	3.21	3.68	3.42	3.68	3.66	3.86	3.65	3.38	3.83
02-03	3.85	3.67	3.70	3.88	4.30	3.98	4.22	4.23	3.89	4.10
02-04	4.53	4.73		4.40		3.34	3.78	2.71	2.68	
02-05	4.06	3.81	3.75	3.94	3.37	3.35	3.88	4.17	3.68	3.87
02-06	3.88	3.91	3.69	4.14	4.12	3.58	3.57	3.23	3.66	3.74
02-08	5.83	4.29	4.02	4.18	3.79	3.70	4.08	3.93	3.78	3.58
02-09	4.00	3.51	3.20	3.25	3.16	3.08	3.10	3.22	3.13	2.83
02-10	3.98	3.83	3.85	4.51	4.20	4.04	3.81	4.01		3.77
02-11	4.57	4.13	4.36	4.56	3.96	3.66	4.26		4.05	4.09
02-14	4.51	5.07	4.43	4.83		4.25	4.57	4.18	3.97	
02-15		4.36	3.98	4.11		4.16	3.94		3.89	3.99

02-16		4.58	4.33	4.68	4.33	3.99	4.42	4.23	3.89	3.91
Median	**4.06**	**4.02**	**3.92**	**4.18**	**4.04**	**3.70**	**3.94**	**4.01**	**3.84**	**3.87**
**Placebo**										
02-07	4.46	4.18	3.18	4.32	3.27	3.46	3.92	3.65	3.51	3.31
02-12	4.33	4.05	4.29	4.38	4.03	3.74	4.27		3.7	4.08
02-13	3.89	3.89	3.44	4.12		3.91	4.09	4.15	3.83	3.88
Median	**4.33**	**4.05**	**3.44**	**4.32**	**3.65**	**3.74**	**4.09**	**3.9**	**3.7**	**3.88**

Table 11. Haemoglobin levels in blood samples of patients who received Panagen or placebo, as measured in different control time points throughout the CT. Normal range is 120 -155 g/L.

	0	Days after the 1st CT			Days after the 2nd CT			Days after the 3rd CT		
		7	14	21	7	14	21	7	14	21
**Панаген**										
02-01	136		127	127	134	131	139	130	131	124
02-02	93	97	111	107	115	109	112	112	108	111
02-03	106	102	102	107	116	108	117	119	112	113
02-04	142	151		117		111	123	88	87	
02-05	130	119	123	128	122	112	127	121	121	127
02-06	118	114	109	123	122	106	109	107	112	114
02-08	165	125	118	128	124	111	124	118	116	108
02-09	120	107	98	103	95	93	92	98	97	87
02-10	114	119	111	126	118	114	107	111		111
02-11	124	106	117	125	103	95	116		114	106
02-14	101	119	106	124		116	128	120	117	
02-15		128	120	124		126	110	110	116	118
02-16		137	130	139	129	119	132	123	114	116
Median	**120**	**119**	**114**	**124**	**120**	**111**	**117**	**115**	**114**	**113**
**Placebo**										
02-07	175	110	108	125	104	100	112	102	101	107
02-12	118	111	119	127	111	108	127		114	121
02-13	118	116	105	129		125	127	128	118	120
Median	**118**	**111**	**108**	**127**	**108**	**108**	**127**	**115**	**114**	**120**

Table 12. Platelet counts in the blood of Panagen- or placebo-group patients measured in different control time points throughout the CT. Normal range is 150-400 $\times 10^9$ cells/L.

	0	Days after the 1st CT			Days after the 2nd CT			Days after the 3rd CT		
		7	14	21	7	14	21	7	14	21
**Panagen**										
02-01	381		258	327	215	298	359	246	194	350
02-02	530	330	299	212	288	117	324	270	181	350
02-03	254	216	255	175	191	142	221	181	161	202
02-04	257	168		255		265	331	92	89	
02-05	252	238	192	265	163	161	364	188	173	362
02-06	331	211	391	403	187	212	303	146	306	427
02-08	178	135	170	252	137	188	337	198	163	280
02-09	232	178	229	461	184	150	436	229	174	293
02-10	155	165	114	244	159	136	176	210		233
02-11	281	240	347	541	274	190	488		219	344
02-14	227	228	183	294		170	231	150	318	
02-15		186	172	288		265	255		152	261
02-16		111	184	263	143	220	282	154	171	265
Median	**254**	**199**	**211**	**265**	**186**	**188**	**324**	**188**	**174**	**293**
**Placebo**										
02-07	324	200	145	251	210	124	359	263	155	266
02-12	264	208	196	370	246	199	274		194	243
02-13	225	228	150	286		180	254	194	161	323
Median	**264**	**208**	**150**	**286**	**228**	**180**	**274**	**229**	**161**	**266**

Table 13. ESR in blood of Panagen- or placebo-group patients at different control time points of the CT. Normal value is below 20 mm/h

	0	Days after the 1st CT			Days after the 2nd CT			Days after the 3rd CT		
		7	14	21	7	14	21	7	14	21
**Panagen**										
02-01	28		14	24	28	23	11	11	13	11
02-02	20	34	28	34	21	31	40	26	7	22
02-03	23	14	10	20	23	35	30	22	12	18
02-04	16			36						
02-05	9	16	14	4	23	20	20	10	8	7

02-06	35	31	28	38		36	38	24	28	38
02-08	11	15	20	24	18	7	17	5	20	10
02-09	57	5	49	38	42	34	32	42	40	50
02-10	7	4	10	3	6	7	15	7		6
02-11	10	20	10	24	20	20	25		10	15
02-14	22	12	35	25		35	40	5	54	
02-15		12	29	36		42	35	30	39	15
02-16		25	5	24	18	44	39	27	33	23
Median	**20**	**15**	**17**	**24**	**21**	**33**	**31**	**22**	**20**	**15**
**Placebo**										
02-07	35	44			23	23	20	13	48	37
02-12	12	20	12	15	15	15	5		20	10
02-13	29	28	24	30		20	20	42	10	54
Median	**29**	**28**	**18**	**23**	**19**	**20**	**20**	**28**	**20**	**37**

Table 14. MCHC in the blood of Panagen- or placebo-group patients in different control time points throughout the CT. Normal MCHC range is 27-34 pg.

	0	Days after the 1st CT			Days after the 2nd CT			Days after the 3rd CT		
		7	14	21	7	14	21	7	14	21
**Panagen**										
02-01			29.9	30.1		29.7	29.8	30.9	32.0	
02-02			30.0		29.6	29.8	29.1	30.6	32.0	29.1
02-03					27.0	27.2	27.8	28.1	28.7	27.6
02-04						33.3	32.5	32.4	32.6	
02-05		31.2	32.8	32.6	36.2	33.5	32.7	29.0	32.9	32.8
02-06	30.4			29.7	29.6	29.6	30.6	33.3	30.6	30.5
02-08		29.1	32.5	30.6	32.7	30.0	30.4	30.0	30.7	
02-09		30.4	30.5	31.6	30.0	30.2	29.7	30.4	31.0	30.7
02-10	28.8	30.9	28.0	27.9	28.1	28.2	28.1	27.8		29.2
02-11	27.0	25.7	26.8	27.4	25.9	26.1	27.2		28.1	26.0
02-14	22.4	23.5	23.9	25.7		27.3	28.0	28.7	29.5	
02-15		29.4	30.1	30.2		30.3	27.8		29.8	29.6
02-16		29.9	30.0	29.7	29.8	29.8	29.9	29.1	29.3	29.7
Median	**27.9**	**29.7**	**30.0**	**29.9**	**29.6**	**29.8**	**29.7**	**30.0**	**30.7**	**29.6**

**Placebo**										
02-07		27.7	28.3	29.0	31.8	29.1	28.6	28.0	28.8	32.3
02-12	27.2	27.4	27.7	29.0	27.5	28.8	29.7		30.8	29.7
02-13	30.3	29.8	30.4	31.3		32.0	31.1	30.8	30.8	30.9
Median	**28.8**	**27.7**	**28.3**	**29.0**	**29.7**	**29.1**	**29.7**	**29.4**	**30.8**	**30.9**

Table 15. Htc levels in the blood of patients from Panagen or placebo groups, measured in different control time points. Normally Htc levels range from 35 to 45%.

	0	Days after the 1st CT			Days after the 2nd CT			Days after the 3rd CT		
		7	14	21	7	14	21	7	14	21
**Panagen**										
02-01	41.9		34.7	34.5	34.7	38.8	39.3	35.2	34.5	37.5
02-02	26.5	26.4	30.5	28.3	34.3	30.5	31.6	29.3	26.8	30.6
02-03		28.2	28.5	30.0	34.7	30.8	32.7	32.7	29.7	31.5
02-04	39.2	41.2				29.5	35.3	23.4	22.6	
02-05	37.2	33.3	32.7	34.5	28.7	29.0	35.9	33.6	33.8	36.0
02-06	32.0	32.3	30.0	36.6	34.2	29.6	29.7	26.2	32.9	33.4
02-08	47.7	35.0	32.5	33.7	29.9	29.8	34.9	32.7	31.8	30.0
02-09		28.6	26.2	26.8	25.7	26.9	26.4	27.8	27.5	24.1
02-10	31.1	29.5	35.6	36.5	34.2	32.9	30.2	31.2		30.9
02-11	36.7	32.0	34.4	36.1	30.1	27.9	33.7		32.5	29.8
02-14	31.7	37.2	32.3	34.1		33.3	37.3	34.8	33.8	
02-15		36.0	32.9	34.6		35.4	30.3		33.2	34.6
02-16		40.2	38.1	40.8	37.4	34.4	39.4	36.6	33.6	34.6
Median	**36.7**	**32.8**	**32.6**	**34.5**	**34.2**	**30.5**	**33.7**	**32.7**	**32.7**	**31.5**
**Placebo**										
02-07	37.1	32.0	30.4	34.3	25.6	31.3	33.0	29.8	28.9	28.1
02-12	35.3	32.5	35.1	36.7	32.7	30.7	36.2		31.6	35.1
02-13	34.1	33.4	29.1	36.1		34.9	36.5	36.6	33.5	34.9
Median	**35.3**	**32.5**	**30.4**	**36.1**	**29.2**	**31.3**	**36.2**	**33.2**	**31.6**	**34.9**

Blood biochemistry profiles in control time points throughout the CT

To evaluate the possible damage to liver parenchyma and to understand whether liver function is compromised, blood levels of intracellular aminotransferases – ALT and AST, bilirubin and bilirubin fractions, alkaline phosphatase – were measured. In order to analyze kidney function, blood creatinine levels were assayed.

ALT is an enzyme catalyzing transamination reaction from L-alanine to a-ketoglutarate, with reaction products being pyruvate and glutamate.

AST catalyzes a similar reaction involving aspartate and a-ketoglutarate resulting in oxaloacetate and glutamate.

These enzymes have specific intracellular localization. ALT is found in the cytosol, whereas AST resides in cytosol and mitochondria. ALT and AST are present in many tissues, but are most concentrated in liver and heart. When integrity of these organs in compromised, ALT and AST concentrations increase, thereby showing an increased cytolysis. It must be noted, that there is significantly more AST than ALT in the cardiac muscle, whereas the opposite is true for the liver. Therefore, the ratio of AST to ALT levels also known as De Ritis ratio is an informative parameter suggestive of the organ damage.

Alkaline phosphatase catalyzes hydrolysis of complex phosphate esters of organic compounds. The enzyme is most abundant in hepatocytes, osteoclasts, less so in liver parenchyma cells and placenta. Higher alkaline phosphatase activity in blood plasma is typically associated with either bone tissue damage or with cholestasis. When bile ducts become obstructed, reactive increase in alkaline phosphatase expression by bile duct endothelial cells occurs.

Bilirubin is a pigment and a breakdown product of haemoglobin. Cells of reticularendothelial system of spleen and bone marrow express hemeoxygenase and biliverdin synthetase whose activity produces free bilirubin. Upon binding to the transport molecule of albumin, bilirubin is delivered to liver parenchyma cells called hepatocytes, where it is converted into conjugated bilirubin via association with two molecules of glucuronic acid. This reaction is catalyzed by UDP-glucuronyl transferase, and it is in this form that bilirubin is excreted with bile into duodenum.

Thus, higher levels of free bilirubin in blood plasma may point at either higher hemolysis of erythrocytes, or at dysfunctional state of hepatocytes that become less efficient in converting free bilirubin into conjugated form. Concurrent changes in both bilirubin fractions indicate combined cytolysis and functional depression of hepatocytes, as is typically observed in toxic hepatitis. Increased levels of conjugated bilirubin suggest compromised bile outflow.

Creatinine is an anhydride of creatine, a final product of protein breakdown. Creatinine is produced in liver, it is transported via the bloodstream from liver to the muscle tissue where it becomes phosphorylated and is thereby converted into phosphocreatine. The latter is a high-energy compound and is used to store energy; when necessary phosphocreatine is broken down to form creatinine. Creatinine is cleared by the kidneys, so higher creatinine levels in peripheral blood in the absence of increased physical exercise may indicate either reduced kidney function or damage to the muscle tissue.

The data obtained are shown in the **Tables 16-20.** Changes in biochemical parameters relatively to the normal values are summarized in the **Table 21**.

Table 16. ALT levels (U/L) in peripheral blood of patients from Panagen and placebo groups, at starting time point on day 0 and in different control time points. Normal ALT range is 10-44 U/L.

	0	Day 21		
		After the 1st CT	After the 2nd CT	After the 3rd CT
**Panagen**				
02-01	35	37	17	
02-02		19	16	54
02-03		10	16	17
02-04	28	30	30	
02-05	18	23	66	73
02-06	13	13	22	48
02-08	19	25	31	38
02-09	44	41	46	65
02-10	14	18	12	17
02-11	34		16	21
02-14	7		6	
02-15	22	25	112	166
02-16		20	28	19
Median	**21**	**23**	**22**	**43**
**Placebo**				
02-07	11	14	18	
02-12	17	30	47	25
02-13	26	22	69	

02-17	20	21		
Median	**19**	**22**	**47**	**25**

Table 17 AST levels (U/L) in peripheral blood of patients from Panagen and placebo groups, at starting time point on day 0 and in different control time points. Normal AST range is 10-34 U/L.

	0	Day 21		
		After the 1st CT	After the 2nd CT	After the 3rd CT
**Panagen**				
02-01	26	23	26	
02-02		21	21	41
02-03		16	19	20
02-04	65	10	20	
02-05	16	22	30	43
02-06	21	18	24	32
02-08	23	30	39	48
02-09	28	35	50	74
02-10	23	22	23	20
02-11	31		29	32
02-14	19	45	23	
02-15	26	34	100	119
02-16		18	19	18
Median	**25**	**22**	**24**	**37**
**Placebo**				
02-07	18	19	24	
02-12	18	33	54	27
02-13	25	27	45	
02-17	20	17		
Median	**19**	**23**	**45**	**27**

Table 18. Alkaline phosphatase levels (U/L) in peripheral blood of patients from Panagen and placebo groups, at starting time point on day 0 and in different control time points. Normal alkaline phosphatase range is 32-126 U/L.

	0	Day 21

		After the 1st CT	After the 2nd CT	After the 3rd CT
**Panagen**				
02-01			73	76
02-02		75		83
02-03		80		79
02-05	61	54	99	95
02-06	129	114	108	139
02-08	93	81	97	93
02-09		341	393	418
02-10		61	54	56
02-11	68		60	64
02-14	106	85	84	
02-15	83	91	105	83
02-16		79	92	67
Median	**88**	**81**	**95**	**83**
**Placebo**				
02-07	54	49	54	
02-12	87		74	68
02-13	75		99	
02-17	94	68		
Median	**81**	**59**	**74**	**68**

Table 19. Bilirubin levels (mkM/L) in peripheral blood of patients from Panagen and placebo groups, at starting time point on day 0 and in different control time points. Normal bilirubin range is 3.4-17 mkM/L.

	0	Day 21		
		After the 1st CT	After the 2nd CT	After the 3rd CT
**Panagen**				
02-01	8.8	5.1	5.3	4.9
02-02		4.8	7.2	6.0
02-03		4.9	10.3	5.0
02-04	6.9	7.3	10.9	

02-05	7.1	10.6	2.8	6.6
02-06	5.0	4.8	4.9	3.9
02-08	5.0	4.0	4.6	4.9
02-09		12.3	5.1	9.9
02-10	8.8	5.1	11.1	4.1
02-11	7.3		5.1	4.4
02-14	6.9		5.9	
02-15	11.9	4.8	4.4	4.9
02-16		4.0	4.6	3.9
Median	**7.1**	**4.9**	**5.1**	**4.9**
**Placebo**				
02-07	5.9	5.1	9.0	
02-12	13.1		5.4	4.0
02-13	11.1	9.1	14.1	
02-17	6.6	3.9		
Median	**8.9**	**5.1**	**9.0**	**4.0**

Table 20. Creatinine levels (mkM/L) in peripheral blood of patients from Panagen and placebo groups, at starting time point on day 0 and in different control time points. Normal creatinine range is 44-97 mkM/L.

	0	Day 21		
		After the 1st CT	After the 2nd CT	After the 3rd CT
**Panagen**				
02-01	82	41	37	
02-02		51	70	27
02-03		63	70	31
02-04	82	100	90	
02-05		64	64	61
02-06	73		67	58
02-08		48	48	49
02-09	61	63	57	55
02-10	77	48	47	
02-11	69		64	49

02-14	65	54	40	
02-15	98	80	59	74
02-16		68	71	61
Median	**75**	**63**	**64**	**55**
**Placebo**				
02-07		59	64	
02-12	48	51	48	34
02-13	50	47	32	
02-17	52	47		
Median	**50**	**49**	**48**	**34**

Table 21. Percentage of patients in Panagen and placebo groups showing abnormal values of blood biochemical parameters before and during the therapy. Except for creatinine, all blood parameters were above the normal range. Creatinine levels in the blood were below the normal range.

Blood parameter	Panagen		Placebo	
	Before the therapy	During the study	Before the therapy	During the study
ALT	0	38	0	50
AST	8	46	0	50
Alkaline phosphatase	8	17	0	0
Bilirubin	0	0	0	0
Creatinin	0	15	0	50

This table demonstrates that increased ALT and AST values are observed in both Panagen- and placebo-groups, which results from the toxic effects of the CT drugs. Yet, the patients additionally receiving Panagen were somewhat less likely to display increased ALT and AST, as compared to the placebo group patients.

Thus, both placebo and Panagen-group patients displayed cytolytic syndrome, which has developed during the study. Notably, hepatic cytolysis syndrome is 1.4-fold more frequent in the placebo group. This can be interpreted as a consequence of cytostatic drugs used throughout the CT, and that this syndrome is alleviated upon taking Panagen.

The percentage of Panagen group patients showing increased alkaline phosphatase levels changed from 8 to 17%, in the absence of notable increase in conjugated bilirubin levels. Thus, the patients in fact underwent destruction of bone tissue, rather than experienced cholestatic syndrome. As we found out, the patients with increased alkaline phosphatase level, had metastatic lesions in locomotor system.

Increased bilirubin levels were not registered in any of the groups.

Reduced concentration of creatinine in the blood of patients from both groups was only observed during but not before the study, and totaled 15% and 50% in Panagen and placebo groups, respectively.

This, Panagen displays a hepatoprotective activity when combined with cytostatic drugs CP, doxorubicin and fluorouracil.

Analysis of side effects associated with Panagen administration to stage II-IV breast cancer patients during three consecutive CT cycles

The primary goal of this analysis was to identify possible side effects associated with Panagen. The following specific goals were established:

1) Identify the patients' symptoms that are related to Panagen administration but not the cytostatic drugs used;

2) Compare the frequencies of side effects caused by the medications used in the study.

Side effects throughout the trial were reported by the patients on day 14 (+/- 1 day) and on day 21 (+/- 1 day) of each cycle of CT. Patients complaints on the day of survey and those experienced between the surveys were recorded. When evaluating the results, only the symptoms that have appeared after taking Panagen medication were taken into account. The results are summarized in the **Table 22**.

Table 22. Frequencies of symptoms reported by Panagen and placebo-group patients (%).

Symptom	Panagen	Placebo
Heartburn	21,4	25
Nausea after the day 7 of the CT cycle	14,3	0
Nausea after the CT (before day 7)	100	100
Pain under ribs on the righthand side after consuming fatty meals	14,3	0
Pain under ribs on the lefthand side after consuming fatty meals	7,1	0
Stabbing periumbilical pain	0	25
Fecal impaction, rare bowel movements	21,4	0
Frequent bowel movements, loose stool	0	25
Flatulence	7,1	0
Metallic aftertaste	7,1	0
Dry mouth	57,1	25
Mouth sores	7,1	25
Throat irritation	14,2	25

Dry eyes	50	25
Dry and flaking skin	35,7	75
Alopecia	100	100
Hive-like allergic reaction	7,1	0
Herpetic eruption	0	25
Café-au-lait spots	7,1	0
Post-injection phlebitis	14,3	0
Joint pain	14,3	0
Backbone pain	7,1	25
Flail legs	7,1	0
Painful urination	7,1	0
Dysfunctional uterine bleeding	7,1	0

Anticancer CT regimen (500 mg/m^{2} CP, 50 mg/m^{2} doxorubicin and 500 mg/m^{2} fluorouracil) was used in this study, hence it was important to filter out the side effects caused the cytostatic drugs.

CP is an alkylating agent belonging to a chloroethylamine group of compounds. Its cytostatic mechanism is based on the formation of a covalent bond between DNA strands. Fluorouracil is an antimetabolite class compound, and is belongs to a group of pyrimidine antagonists. Active metabolites of fluorouracil cause two distinct effects. First, they inhibit an enzyme called thymidylsynthetase, thereby reducing the pool of available thymidine and inhibiting DNA synthesis. Second, they outcompete uridine triphosphate and become incorporated into nascent RNA molecules thereby compromising proper processing of RNA. Doxorubicin in an anthracycline antibiotic. It intercalates into double-stranded DNA, which alters its spatial configuration. Doxorubicin also compromises DNA molecule integrity, because it induces formation of free radicals.

Thus, different molecular pathways are involved in the potent cytostatic activity of these drugs. Cytostatic drugs primarily target actively proliferating cells. Besides cancerous cells, a number of other cell types also display active proliferation, namely hematopoietic cells, enterocytes, hair follicle cells, as well as basal layer epithelial cells. This underlies the wide range of side effects observed for these drugs.

Side effects of cytostatic drugs used in the study

Doxorubicin

Cardiovascular system: sinus node tachycardia and/or abnormalities of ECG. Also noted were tachyarrhythmia, VPB, bradycardia, AV block and BBB. Subsequent impaired myocardium function manifests as decline in asymptomatic LVEF with or without CCF. Phlebitis, thrombophlebitis, thromboembolic complications including pulmonary embolism.

Digestive system: anorexia, nausea, vomiting, stomatitis, esophagitis, oral mucosal hyperpigmentation, abdominal pain, gastrointestinal haemorrhages, diarrhea, colitis. Increased total bilirubin and liver transaminase activity in blood plasma.

Urinary system: red-colored urine during the first 1-2 days after taking doxorubicin.

Ocular: conjunctivitis, keratitis, increased tear excretion

Reproductive system: amenorrhoea, oligospermia, azoospermia

Cutaneous and skin structures: Reversible complete alopecia occurs in most cases. Skin and nail hyperpigmentation, light sensitivity, hives, rash, itching.

Allergic reactions: skin rash, dermatitis, hives, palm and feet skin hyperaemia, bronchospasm, anaphylaxis.

Local reactions: erythematous streaking along the infusion vein, subsequent local phlebitis or trombophlebitis are not infrequent. Phlebosclerosis may develop, particularly it may result from repeated injection of doxorubicin into the same small vein. In case of extravasation into surrounding tissues, local painful senstation, severe cellulitis and tissue necrosis may develop.

Upon intra-arterial injection: besides systemic toxicity, ulceration of stomach and duodenum, as well as common biliary duct stenosis resulting from drug-induced sclerosing cholangitis may occur.

CP

Digestive system: nausea, vomiting, diarrhea, stomach pain; rarely – toxic hepatitis.

Hemopoietic system: leucopenia, thrombocytopenia, anemia

Respiratory system: upon long-term high-dose administration – pneumonitis or interstitial pulmonary fibrosis.

Cardiovascular system: tachycardia, dyspnea, acute pericarditis; in rare cases – severe cardiac failure (secondary to hemorrhagic myocarditis and myocardial necrosis).

Urinary system: aseptic hemorrhagic cystitis, nephropathy.

Reproductive system: menstrual disorders, amenorrhoea, azoospermia.

Allergic reactions: skin rash, hives, anaphylactic reactions.

Other reactions: alopecia, muscle pain, bone pain and headache.

Fluorouracil

Digestive system: diarrhea, mouth sores, esophagitis, nausea, vomiting.

Hemopoietic system: anemia, leucopenia, thrombocytopenia.

Blood coagulation system: haemorrhages

CNS: ataxia, optic neuritis, headache, visual impairment, photophobia, euphoria, confusion, disorientation.

Cardiovascular system: myocardial eschemia, angina, thrombophlebitis.

Reproductive system: azoospermia, amenorrhea.

Skin reactions: alopecia, dermatitis, hyperpigmentation.

Allergic reactions: hives, bronchospasm.

The data presented above indicate that almost all side effects reported during the study are attributable to the cytostatic drugs used. The only notable exception is flail legs, which was a complication due to breast cancer metastases affecting the second lumbar vertebra.

It must also be noted that the Panagen-group patients were in general more prone to exocrine insufficiencies and rare bowel movements **(Table 22).**

A number of symptoms such as metallic aftertaste, flatulence, pain under lefthand side ribs, painful urination, hive-like allergic reactions, café-au-lait skin pigmentation, nausea after day 7 of the CT cycle, joint pain – were reported in isolated instances in Panagen-group patients. In all likelihood these symptoms are not caused by Panagen, but rather are identified due to the larger size of Panagen sampling, therefore they have higher chances to be reported as part of cytostatics-related side effects.

It must be noted that patients complained for dry mouth and dry and flaking skin less often if they received Panagen, as compared to those receiving placebo. This may result from improved regeneration of epithelium, which may in turn be caused by targeting of epithelial basal cells by exogenous DNA.

The analysis thus performed can be summarized as follows:

1. The medication studied here does not cause side effects than those associated with the use of cytostatics, CP, doxorubicin and fluorouracil.
2. Possible side effects associated with Panagen therapy include reduced secretory function of salivary glands and accessory lacrimal glands, as well as rare bowel movements.
3. Positive side effects of Panagen medication include improved regeneration of surface epithelium, which likely results from Panagen targeting and improving proliferation of basal cells.

Self-perceived quality of life assessment by the patients enrolled in the study (QoL scale)

The following results became apparent when patients responses to QLQ-C30 questionnaires were processed (**Table 23).**

Median of patients' global health values in the Panagen group equals 50.0 points, and stays so throughout the study, whereas in placebo-group patients it is reduced to 41.7 by the end of the study.

Patients' functional state in both groups is very close and remains unchanged throughout the study.

Severity of clinical symptoms in Panagen-group patients was initially four-fold lower than in the placebo group. By the end of the study, in Panagen cohort it remained at the same level, whereas it dropped two-fold in the placebo cohort.

Table 23. Data analysis of patients' responses to QLQ-C30 questionnaire. Scale ranged from 0 to 100.

	Global health				Functional scale				Symptomatic scale			
	Day 0	After the 1st CT	After the 2nd CT	After the 3rd CT	Day 0	After the 1st CT	After the 2nd CT	After the 3rd CT	Day 0	After the 1st CT	After the 2nd CT	After the 3rd CT
**Panagen**												
02-01			50.0	50.0			77.8	86.7			17.9	10.3
02-02	50.0	33.3	66.7	50.0	75.6	62.2	80.0	64.4	15.4	23.1	12.8	25.6
02-03		66.7	83.3	66.7		66.7	93.3	84.4		46.2	17.9	17.9
02-04	41.7	16.7	33.3		57.8	24.4	26.7		38.5	66.7	51.3	
02-05	83.3		66.7		97.8		97.8		0.0		12.8	
02-06	50.0	50.0	66.7	66.7	82.2	73.3	75.6	88.9	7.7	15.4	15.4	10.3
02-08		83.3	83.3	100.0		95.6	95.6	95.6		5.1	2.6	5.1
02-10	83.3	83.3			97.8	97.8			0.0	10.3		
02-11	58.3	41.7	50.0	33.3	77.8	80.0	73.3	73.3	25.6	35.9	15.4	15.4
02-14	41.7	41.7		50.0	82.2	84.4	86.7	86.7	12.8	10.3	12.8	12.8
02-15	41.7	75.0	66.7		100.0	97.8	95.6		7.7	2.6	7.7	
Median	**50.0**	**50.0**	**66.7**	**50.0**	**82.2**	**80.0**	**83.3**	**86.7**	**10.3**	**15.4**	**14.1**	**12.8**
**Placebo**												
02-07		83.3	83.3			82.2	97.8			5.1	7.7	
02-12	50.0	33.3	33.3	33.3	71.1	75.6	93.3	91.1	35.9	28.2	7.7	15.4

02-13	50.0	50.0	50.0	50.0	91.1	86.7	77.8	82.2	46.2	28.2	25.6	23.1
Median	**50.0**	**50.0**	**50.0**	**41.7**	**81.1**	**82.2**	**93.3**	**86.7**	**41.0**	**28.2**	**7.7**	**19.2**

QLQ-BR23 questionnaire was specifically developed for breast cancer patients, and our analysis of QLQ-BR23 responses suggest that functional activity of Panagen-group patients remained stable throughout two consecutive CT cycles, and increased from 30 to 40 points by the third CT. Notably, in placebo cohort, the functional activity values dropped by 15-25% at intermediate control time points, and initial levels were never achieved by the end of the third CT (**Table 24).**

Severity of clinical symptoms of Panagen arm participants was significantly increased two-fold ($p<0.05$, paired Wilcoxon test). Placebo group patients displayed lower scores on the symptomatic scale.

Table 24. Data analysis of patients' responses to QLQ- BR23. Scale ranged from 0 to 100.

	**Functional scale**				**Symptomatic scale**			
	Day 0	After the 1st CT	After the 2nd CT	After the 3rd CT	Day 0	After the 1st CT	After the 2nd CT	After the 3rd CT
**Panagen**								
02-01	33.3	33.3	29.2	54.2	16.3	40.3	38.2	26.3
02-02	50.0	50.0	56.3	58.3	7.9	28.3	14.3	15.2
02-03	6.3	8.3	33.3		50.3	47.9	37.5	
02-04	25.0	16.7	8.3		29.6	54.0	21.3	
02-05	31.3	33.3	33.3		2.1	23.8	3.3	
02-06	39.6	25.0	33.3	12.5	10.7	19.6	18.8	17.6
02-08	33.3	33.3	41.7	41.7	4.5	11.3	8.0	5.7
02-10	52.1	58.3			9.7	10.3		
02-11	50.0	41.7	45.8	45.8	11.2	37.3	30.6	29.0
02-14	25.0	20.8	25.0	8.3	16.1	42.7	21.7	24.0
02-15	29.2	14.6	45.8		2.4	34.9	34.5	
Median	**33.3**	**33.3**	**33.3**	**43.8**	**10.7**	**34.9**	**21.5**	**20.8**
**Placebo**								
02-07	79.2	41.7	50.0		26.7	26.5	0.0	
02-12	22.9	8.3	50.0	41.7	31.3	33.5	9.7	9.7
02-13	66.7	75.0	58.3	83.3	23.2	23.2	19.9	19.9
Median	**66.7**	**41.7**	**50.0**	**62.5**	**26.7**	**26.5**	**9.7**	**14.8**

When we compared patients' responses to both questionnaires, we saw that the analyzed parameters displayed opposing trends. Whereas global health of Panagen-group patients stayed the same by the end of the third CT, localized clinical presentation was more severe in this group vs placebo patients. This more severe clinical symptomatic pattern could result from either inefficient anticancer therapy (when combined with Panagen), or, on the contrary, be due to boosted immune response, which resulted in increased local inflammatory reaction.

Thus, administration of Panagen in combination with a chemotherapeutic FAC regimen to treat breast cancer leads to the significant increase in localized symptomatic reactions in the lesion area as compared to the pre-therapy level. A trend for higher functional activity of patients receiving Panagen is noted.

Concluding remarks

1. The critical time point after the cytostatic therapy is day 14 after injection of cytostatics. It is at this point that all blood cell counts drop to minimum values. Panagen was established to have protective and stimulatory activity towards committed neutrophil progenitors. This results in higher peripheral-blood neutrophil counts in Panagen-group patients and in reduced occurrence of grade I-IV neutropenias in the control time point on day 14 after the third CT. A number of facts support the idea that provided that Panagen is used, critical neutrophil count on day 14 post injection of cytostatics should not necessarily call for G-CSF leukostimulatory intervention. When Panagen is used in combination with cytostatics, 1-3 days after the neutrophils drop to critical level, this parameter will be restored to a non-critical level, and will be completely normal by the 21 day after start of CT cycle. This allows the patient to proceed with the CT cycles according to the schedule, which is of utmost importance for efficient cancer therapy.

2. Our studies argue in favor of Panagen acting to protect and stimulate the leukopoietic progenitors across three FAC CT cycles to treat stage II-IV breast cancer. In all the crucial time points (day 21 after each CT cycle) throughout the cycles of cytoreductive CT this parameter was superior in the Panagen group to that in the placebo-group patients. On day 21 after the third CT this parameter was significantly ($p<0.05$, Wilcoxon-Mann-Whitney test) higher than in the placebo cohort. This indicates that Panagen safeguards the leukopoietic lineage from increasingly negative effects of cytostatic cytoreductive therapy throughout 3 consecutive CT cycles and stimulates proliferation of HSCs that give rise to this lineage.

Effects of Panagen on various pluripotent hematopoietic cell lineages, such as progenitors of neutrophil, monocyte, lymphocyte and erythropoietic lineage were studied separately.

3. It was shown that Panagen affect on activation of the neutrophil lineage cell proliferation. We demonstrated that statistically significant effect is observed on day 21 after the second CT. Notably, in all control time points on day 21 after 1, 2 and 3 CT this parameter scored higher in the Panagen cohort than in the placebo group of patients. This result points to the fact that Panagen displays protective and stimulatory activity towards committed progenitors of neutrophil lineage.

The most striking was the effect of Panagen on proliferation of monocytes. Despite the differences within the sampling were insignificant due to the large variability of parameter values in individual patients, the median values for Panagen group were much higher than those in the placebo group. As it follows from our data, by the end of the third CT the effect of the medication is the most pronounced as compared to the earlier control time points, which may

indicate that Panagen effects accumulate throughout the CT to induce monocyte proliferation. When the data were analyzed after the Panagen-group patients were split into responders and non-responders as defined by their monocyte counts on day 14 after the third CT, we saw that the responders had more than 4-fold higher values than placebo-group patients, and more than 8-fold higher values than did non-responders.

3. Lymphocyte counts in Panagen group are insignificantly higher as compared to the placebo-cohort. Yet, much as it was observed for other blood cell types, the values tend to be higher in Panagen-group patients at the very last control time point of the study. This observation also supports a positive role of Panagen in protecting and stimulating proliferation of lymphoid cell lineage. The same holds true when the Panagen-group patients are subdivided into responders and non-responders. Namely, on day 21 after the second CT peripheral-blood lymphocyte counts are statistically higher in Panagen-responders than in placebo or Panagen-non-responders ($p<0.1$, Wilcoxon-Mann-Whitney test).

4. As our analysis shows, Panagen has little if any activity towards the erythropoietic lineage. Nevertheless, the following effects of Panagen were found. Whereas 100% of placebo-group patients displayed reduced haemoglobin levels, only 63% of Panagen-group patients had low haemoglobin. This may attest to the protective activity of Panagen. Furthermore, we observed increased platelet counts by the day 21 after the CT in 23% of Panagen-group patients.

5. Panagen displays hepatoprotective activity when combined with CT drugs CT, doxorubicin and fluorouracil.

6. Possible side effects associated with Panagen include lower secretory function of salivary glands and accessory lacrimal glands, and rare bowel movements. Positive side effects of Panagen include improved regeneration of surface epithelium, which is likely attributable to Panagen targeting of basal cells in the skin leading to their increased proliferation.

7. Assessment of patients' quality of life shows that administration of Panagen in combination with the FAC CT treatment of breast cancer results in significantly stronger symptomatic manifestations at the lesion site as compared to the initial level. Inclusion of Panagen into the FAC therapy tends to result in an overall increased functional activity of the patients.
