# Supplementary material for: Results of multicenter double-blind placebo-controlled phase II clinical trial of Panagen preparation to evaluate its leukostimulatory activity and formation of the adaptive immune response in patients with stage II-IV breast cancer
Source: BMC Cancer. 2015 Mar 13;15:122. doi: 10.1186/s12885-015-1142-z (PMC4365563; doi:10.1186/s12885-015-1142-z)
Supplement: Additional file 3: — Results of the phase II double-blind multicenter placebo-controlled clinical trial to evaluate the safety and leukostimulatory activity of Panagen in breast cancer patients.The study was performed at the Oncology Department of Novosibirsk Municipal Hospital No 1 and included 26 patients receiving AC therapy (19 patients additionally received Panagen, 7 patients received placebo). [file 12885_2015_1142_MOESM3_ESM.pdf]

Additional file 3. Results of the phase II double-blind multicenter placebo-controlled clinical trial to evaluate the safety and leukostimulatory activity of Panagen in breast cancer patients. The study was performed at the Oncology Department of Novosibirsk Municipal Hospital No 1 and included 26 patients receiving AC therapy (19 patients additionally received Panagen, 7 patients received placebo).

After the run-in period, the patients scheduled for AC therapy (combination of cyclophosphamide and doxorubicin) were enrolled in the study. A total of 31 patients participated in the study, of which 20 patients formed the Panagen group, and 11 patients received placebo. Subsequently, 1 Panagen- and 4 placebo-group patients discontinued the study.

Panagen activity toward lymphoid lineage. Dynamics of various types of leukocytes in the peripheral blood of patients enrolled in the trial

Parameters were compared both directly and by normalizing to the values observed after the first chemotherapy (CT) cycle. This approach allowed us to characterize the effects of Panagen (protection, enhanced proliferation) towards the lymphoid lineage along with the increasingly negative effects of consecutive CT courses.

When analyzing how lymphoid lineage cells are affected by Panagen, we subgrouped the patients receiving Panagen into responders and non-responders. This allowed us to measure the efficiency of the medication across different control time points and to describe the striking effect of patients switching from responders into non-responders and vice versa. This trend suggests the importance of taking into account seemingly scattered individual values and performing relative comparisons between the groups. In other words, if comparisons performed within the total data set fail to demonstrate significant differences, this does not automatically mean that no differences exist. Upon proper sub-grouping of patients into responders and non-responders, such differences may be recognized.

Analysis of leukocyte cell counts in the control time points during three courses of CT

The state of lymphoid lineage progressively deteriorates along the consecutive courses of CT, unless Panagen is co-administered to buffer these effects and to protect lymphoid progenitor cells. Leukocyte counts persistently decrease in the placebo-group, but not in the Panagen-group patients, where they remain essentially the same as they were before the CT. It must be noted,

that the starting absolute leukocyte counts in the placebo group are significantly higher than those in Panagen-group patients. This may indicate that placebo patients initially appear somewhat “healthier”, yet this does not protect them from the negative impact of leukodepressive cytostatic therapy (**Table 1, Figure 1**).

Table 1. WBC counts ($\times 10^9$ cells/L) in Panagen- and placebo-group patients, as measured at different control time points after three courses of CT.

	Day 0	After the 1st CT			After the 2nd CT			After the 3rd CT		
		Day 7	Day 14	Day 21	Day 7	Day 14	Day 21	Day 7	Day 14	Day 21
**Panagen**										
02-20	4.7	1.4	1.4	3.5	1.8	1.5	4.4	2.6	1.4	3.5
02-21	7.9	3.7	3.2	7.3	6.1	2.7	8.5	4.1	2.3	7.1
02-22	5.0	3.9	2.7	6.2	4.3	3.1	7.3	4.9	4.2	
02-24	12.2	7.2	4.1	8.0	6.8	4.2	9.3	5.4	4.5	10.0
02-25	4.1	3.3	1.4	5.9	2.9	1.1	4.0	2.6	1.5	4.1
02-26	6.1	4.2	2.6	5.4	4.1	2.4	7.5	3.4	4.2	7.0
02-27	8.8	2.5	2.4	6.2	4.1	2.6		9.9	2.2	5.6
02-28	5.9	4.2	3.1	6.5	4.1	2.3	7.0		2.0	
02-29	8.0	5.4	2.6	6.5	3.4	2.2	6.0	3.5	3.5	7.3
02-30		3.7	3.4	4.3	3.3	2.3	4.0	2.3	1.8	
02-31	4.4	3.6	2.8	5.3	6.5		6.5	3.0		6.7
02-33	10.6	5.9	3.1	10.5	6.5	3.8	10.9	3.5	4.3	7.4
02-36	5.9	4.2	3.0	6.0	6.9	5.4	5.5			
02-39	3.9	2.1	0.9	5.3	2.2	1.0	5.1	4.2	0.9	4.1
02-40	4.8			5.4	3.5		3.9	2.8	1.4	4.6
02-42	5.1	1.2	1.4	3.2	2.6	1.2	3.7	3.4	3.0	3.3
02-43	6.0	3.7	2.6	8.8	5.6	2.8	7.9	6.1	4.1	3.3

02-44	7.7	4.5	2.5	9.2	3.8	2.1	6.6	3.8	4.2	6.0
02-45	4.2	2.0	1.4	4.6	2.1	1.4	4.7	2.2	1.6	4.6
n	18	18	18	19	19	17	18	17	17	15
Median	**5.9**	**3.7**	**2.6**	**6.0**	**4.1**	**2.3**	**6.3**	**3.5**	**2.3**	**5.6**
Minimum	3.9	1.2	0.9	3.2	1.8	1.0	3.7	2.2	0.9	3.3
Maximum	12.2	7.2	4.1	10.5	6.9	5.4	10.9	9.9	4.5	10.0
Lower Quartile	4.7	2.5	1.4	5.3	2.9	1.5	4.4	2.8	1.6	4.1
Upper Quartile	7.9	4.2	3.1	7.3	6.1	2.8	7.5	4.2	4.2	7.1
**Placebo**										
02-23	7.5	5.3			4.8	3.5	3.8	3.9	2.4	2.9
02-32		4.1	3.2	9.6	4.5	2	5.8			7.4
02-34		3.1	1.5	6.1	2.7	1.2	1.6	1.1		3.4
02-35	6.4	4.1	2.0	7.4	3.3	1.7	5.9	3.4		6.4
02-37	8.6	4.5	6.5	8.8	5.6	4.2	8.2	5.8	2.5	9.3
02-38	16.0	5.3	3.4	7.9	6.4		5.4			
02-41	10.6	5.7	2.7	10.8	6.2	3.3	9.3		4.8	
n	5	7	6	6	7	6	7	4	3	5
Median	**8.6**	**4.5**	**3.0**	**8.4**	**4.8**	**2.7**	**5.8**	**3.7**	**2.5**	**6.4**
Minimum	6.4	3.1	1.5	6.1	2.7	1.2	1.6	1.1	2.4	2.9
Maximum	16.0	5.7	6.5	10.8	6.4	4.2	9.3	5.8	4.8	9.3
Lower Quartile	7.5	4.1	2.0	7.4	3.3	1.7	3.8	2.3	2.4	3.4

Upper Quartile	10.6	5.3	3.4	9.6	6.2	3.5	8.2	4.9	4.8	7.4

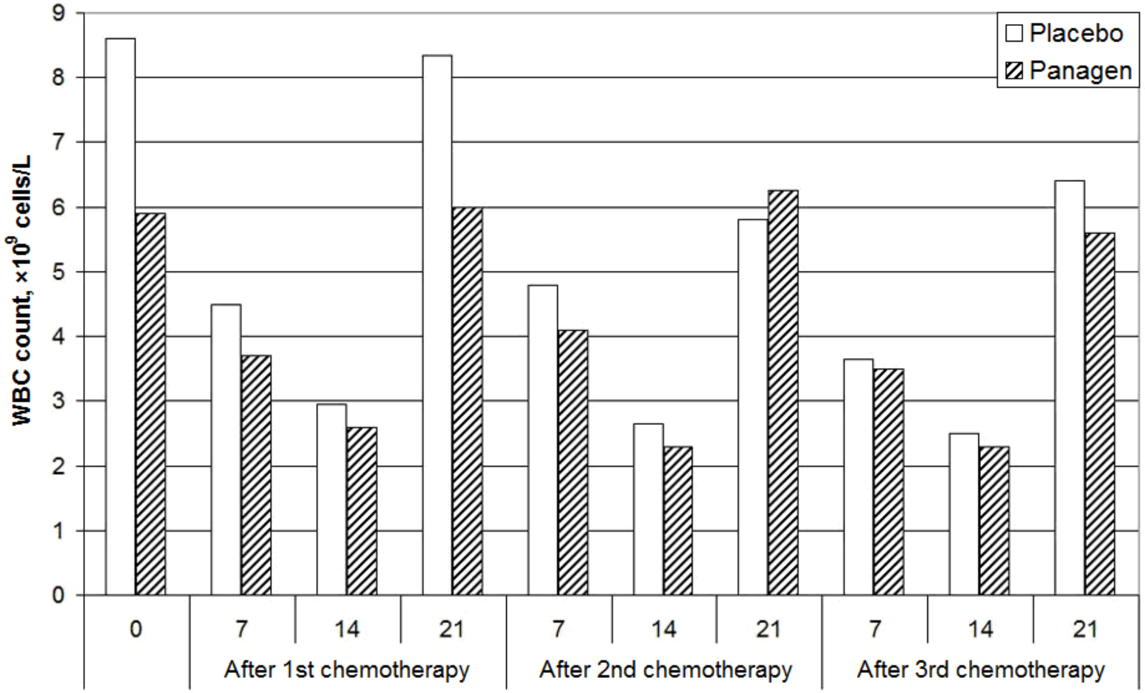

Figure 1. WBC counts at control time points on day 0, and days 7, 14 and 21 of the three CT courses. Bars denote median values in groups.

As it is shown on the plots, Panagen has leukostimulatory activity, which is best manifested on day 21 after the second and third CT. The values observed in responding patients are significantly higher than in placebo group, and the combined percentage of responding patients totals 58% (**Table 2, Figure 2**).

Table 2. Relative WBC levels (%) in Panagen- and placebo-group patients on days 14 and 21 after the 2nd and 3rd CT, normalized to the levels after the 1st CT course.

	Day 14		Day 21	
	After the 2nd CT	After the 3rd CT	After the 2nd CT	After the 3rd CT
**Panagen**				
02-20	107.1	100.0	125.7	100.0
02-21	84.4	71.9	116.4	97.3
02-22	114.8	155.6	117.7	
02-24	102.4	109.8	116.3	125.0
02-25	78.6	107.1	67.8	69.5
02-26	92.3	161.5	138.9	129.6
02-27	108.3	91.7		90.3
02-28	74.2	64.5	107.7	
02-29	84.6	134.6	92.3	112.3
02-30	67.6	52.9	93.0	
02-31			122.6	126.4
02-33	122.6	138.7	103.8	70.5
02-36	180.0		91.7	
02-39	111.1	100.0	96.2	77.4
02-40			72.2	85.2
02-42	85.7	214.3	115.6	103.1
02-43	107.7	157.7	89.8	37.5
02-44	84.0	168.0	71.7	65.2
02-45	100.0	114.3	102.2	100.0
Median value	**100.0**	**112.0**	**103.0**	**97.3**
Percent responders, %	53	75	56	47
Percent non-responders, %	47	25	44	53
Median value for responders	**108.3**	**136.7**	**116.3**	**112.3**

Median value for non-responders	84.2	68.2	90.7	73.9
Combined percentage of responders, %	**82**		**58**	
**Placebo**				
02-32	62.5		60.4	77.1
02-34	80.0		26.2	55.7
02-35	85.0		79.7	86.5
02-37	64.6	38.5	93.2	105.7
02-38			68.4	
02-41	122.2	177.8	86.1	
Median value	**80.0**	**108.1**	**74.0**	**81.8**

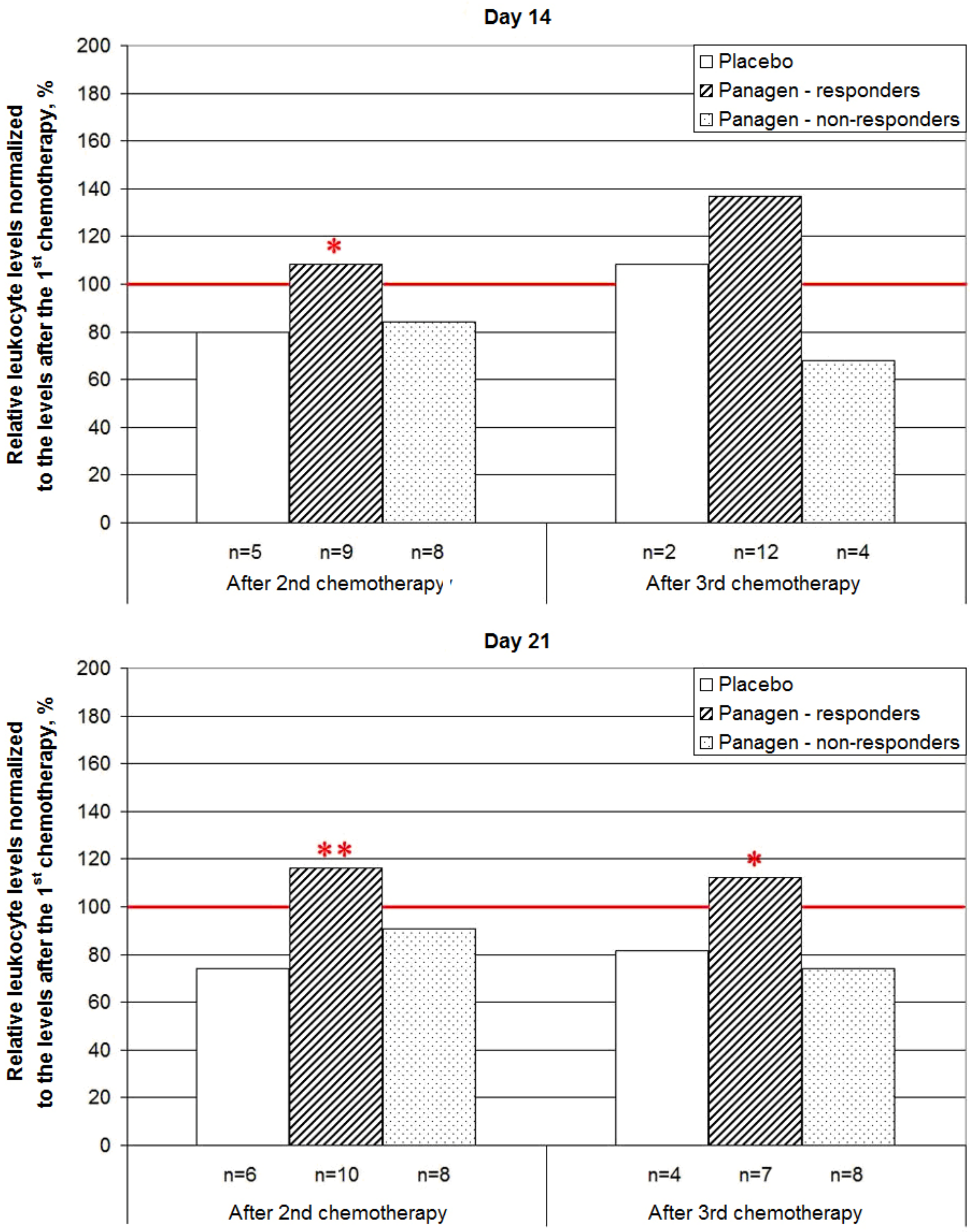

Figure 2. Relative peripheral-blood WBC levels in patients on day 14 and 21 after the 2nd and 3rd CT courses normalized to the levels observed after the 1st CT set to 100% (red line).
Red asterisks denote significant difference from the placebo group values (single asterisk, $p<0.1$, and double asterisk, $p<0.01$, Wilcoxon-Mann-Whitney test).

Measurements of neutrophil counts at control time points across the three cycles of CT

Dynamics of neutrophil cell counts is very similar to that of the leukocytes. Significant decrease in neutrophil counts is observed in the placebo cohort throughout three consecutive courses of CT. On day 21 after the 2nd and 3rd CT, the neutrophil counts are significantly lower in this group than at the starting point ($p<0.05$ and $p<0.11$, respectively, Wilcoxon-Mann-Whitney test). Notably, the neutrophil counts in Panagen-group patients remain the same across the three CT courses and matches that of the starting day 0 time point (**Table 3, Figure 3**).

Table 3. Neutrophil counts ($\times 10^9$ cells/L) in patients from Panagen and placebo cohorts, measured in control time points after three cycles of CT.

	Day 0	After the 1st CT			After the 2nd CT			After the 3rd CT		
		Day 7	Day 14	Day 21	Day 7	Day 14	Day 21	Day 7	Day 14	Day 21
**Panagen**										
02-20	3.76	0.98	0.81	2.17	1.48	0.75	2.29	1.95	0.56	2.42
02-21	5.53	2.37	1.63	5.18	4.45	0.97	5.61	2.54	0.48	5.04
02-22	3.00	2.42	1.84	4.03	2.97	1.05	4.16	2.84	2.06	
02-24	9.52	4.97	1.64	4.88	4.90	1.18	4.56	3.40	2.07	8.50
02-25	2.34	2.51	0.95	4.07	2.29	0.24	3.24	1.85	0.39	2.87
02-26	3.97	3.11	1.01	1.67	2.21	0.58	4.80	2.55	1.26	4.20
02-27	6.07	1.83	0.91	3.72	3.03	1.30		7.33	0.97	3.81
02-28	3.10	2.18	0.65	2.93	2.46	0.53	4.20		1.02	
02-29	5.60	4.27	0.65	3.25	2.48	1.12	3.78	2.66	2.56	4.53
02-30		1.96	1.05	1.81	2.01	1.17	1.60	1.01	1.04	
02-31	2.29	2.23	1.01	3.66	5.85		4.68	2.04		4.02
02-33	4.56	3.07	0.96	6.41	4.75	2.24	6.98	2.21	2.06	5.11
02-36	3.60	2.14	1.56	2.68	3.52	3.62	3.19			
02-39	2.77	1.45		3.92	1.85	0.61	3.47	3.28	0.58	3.28
02-40	3.31			3.35			2.69	1.79	0.62	2.81
02-42	3.32			1.70	1.69	0.79	2.63	2.28	1.32	1.55
02-43	3.48	2.78	1.61	6.42	4.26	1.74	4.11	4.09	1.93	1.65

02-44	4.31	2.93	0.63	5.98	2.43	1.32	4.29		2.27	4.26
02-45	2.65	0.98	0.43	2.85	1.45	0.60	3.34	1.74	0.83	3.73
n	18	17	16	19	18	17	18	16	17	15
Median	**3.54**	**2.37**	**0.98**	**3.66**	**2.47**	**1.05**	**3.94**	**2.41**	**1.04**	**3.81**
Minimum	2.29	0.98	0.43	1.67	1.45	0.24	1.60	1.01	0.39	1.55
Maximum	9.52	4.97	1.84	6.42	5.85	3.62	6.98	7.33	2.56	8.50
Lower Quartile	3.00	1.96	0.73	2.68	2.01	0.61	3.19	1.90	0.62	2.81
Upper Quartile	4.56	2.93	1.59	4.88	4.26	1.30	4.56	3.06	2.06	4.53
**Placebo**										
02-23	6.30	3.98			3.70	2.10	1.67	2.57	1.56	1.74
02-32		2.62	0.70	6.82	3.38	1.18	3.89			4.37
02-34		2.48	0.18	4.09	1.67		0.80			2.38
02-35	4.29	2.67	0.20	3.92	1.78	0.65	3.95	2.48		3.52
02-37	5.33	2.66	4.03	5.72	4.31	2.14	5.25	3.65	1.28	5.02
02-38	11.04	3.34	1.46	4.82	4.16		3.51			
02-41	7.31	4.22	0.92	8.32	3.84	1.55	6.05		1.82	
n	5	7	6	6	7	5	7	3	3	5
Median	**6.30**	**2.67**	**0.81**	**5.27**	**3.70**	**1.55**	**3.89**	**2.57**	**1.56**	**3.52**
Minimum	4.29	2.48	0.18	3.92	1.67	0.65	0.80	2.48	1.28	1.74
Maximum	11.04	4.22	4.03	8.32	4.31	2.14	6.05	3.65	1.82	5.02
Lower Quartile	5.33	2.62	0.20	4.09	1.78	1.18	1.67	2.48	1.28	2.38

Upper Quartile	7.31	3.98	1.46	6.82	4.16	2.10	5.25	3.65	1.82	4.37

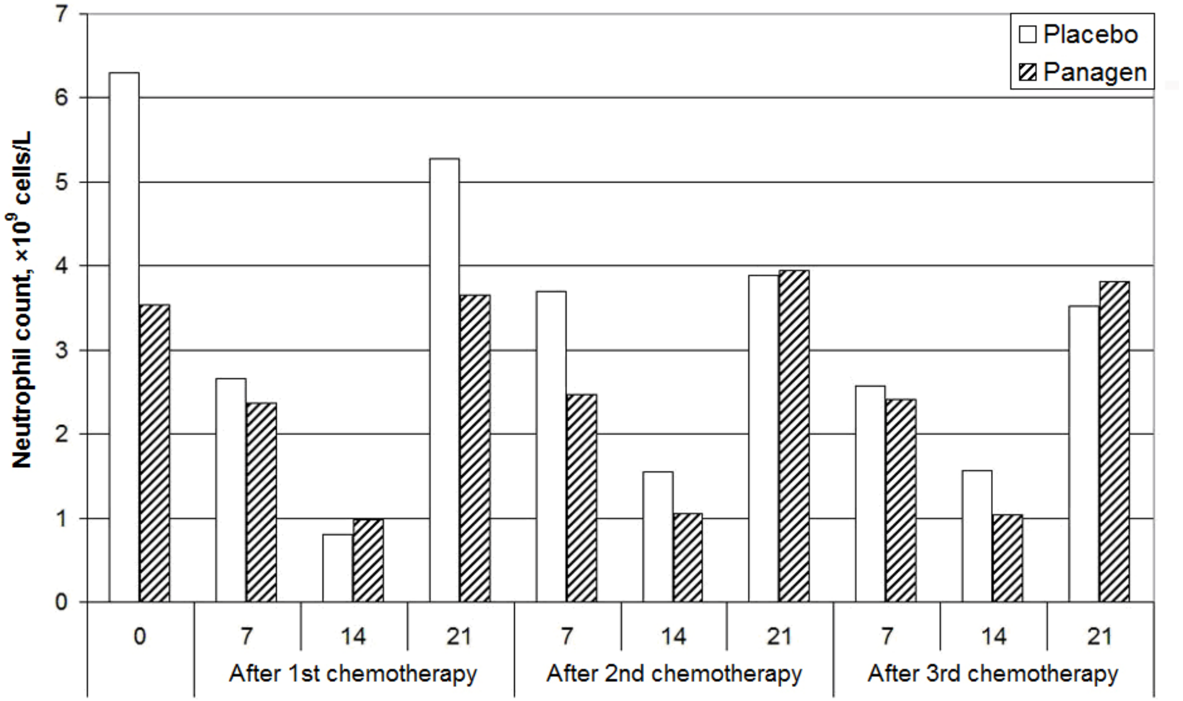

Figure 3. ANC counts at the starting time point on day 0 and at the control points on days 7, 14 and 21 after three cycles of CT. Bars denote median values for each group. Bars denote median values in groups.

We present comparison of responding and non-responding patients. Significant increase among Panagen-responders is notable (**Table 4, Figure 4**). It must be pointed that percentage of responding patients at the control time point on day 21 after the second and third CT is 61% and 74%. This is consistent with the sensitivity of neutrophil progenitor lineage to the medication in most Panagen-group patients.

Table 4. Relative neutrophil levels (%) on days 14 and 21 after the 2nd and 3rd CT rounds normalized to the levels after the 1st CT.

	Day 14		Day 21	
	After the 2nd CT	After the 3rd CT	After the 2nd CT	After the 3rd CT
**Panagen**				
02-20	92.4	69.0	105.4	111.3
02-21	59.6	29.6	108.2	97.3
02-22	57.4	112.1	103.3	
02-24	71.7	126.2	93.4	174.2
02-25	25.4	41.0	79.6	70.5
02-26	56.8	124.3	286.7	250.9
02-27	142.5	106.1		102.4
02-28	81.3	156.7	143.6	
02-29	172.6	393.1	116.3	139.3
02-30	111.3	99.1	88.6	
02-31			128.0	109.9
02-33	233.3	214.8	108.9	79.7
02-36	231.9		119.2	
02-39			88.4	83.6
02-40			80.4	83.8
02-42			154.9	91.5
02-43	107.7	119.5	63.9	25.7
02-44	211.7	362.9	71.7	71.2
02-45	138.7	191.7	117.0	130.6
Median value	**107.7**	**121.9**	**106.8**	**97.3**
Percent responders, %	53	71	61	47

Percent non-responders, %	47	29	39	53
Median value for responders	**157.6**	**141.5**	**117.0**	**130.6**
Median value for non-responders	59.6	55.0	80.4	81.7
Combined percentage of responders, %	**80**		**68**	
**Placebo**				
02-32	167.6		57.0	64.1
02-34			19.6	58.2
02-35	323.0		100.8	89.8
02-37	53.2	31.6	91.7	87.8
02-38			72.8	
02-41	169.0	198.7	72.7	
Median value	**168.3**	**115.2**	**72.8**	**75.9**

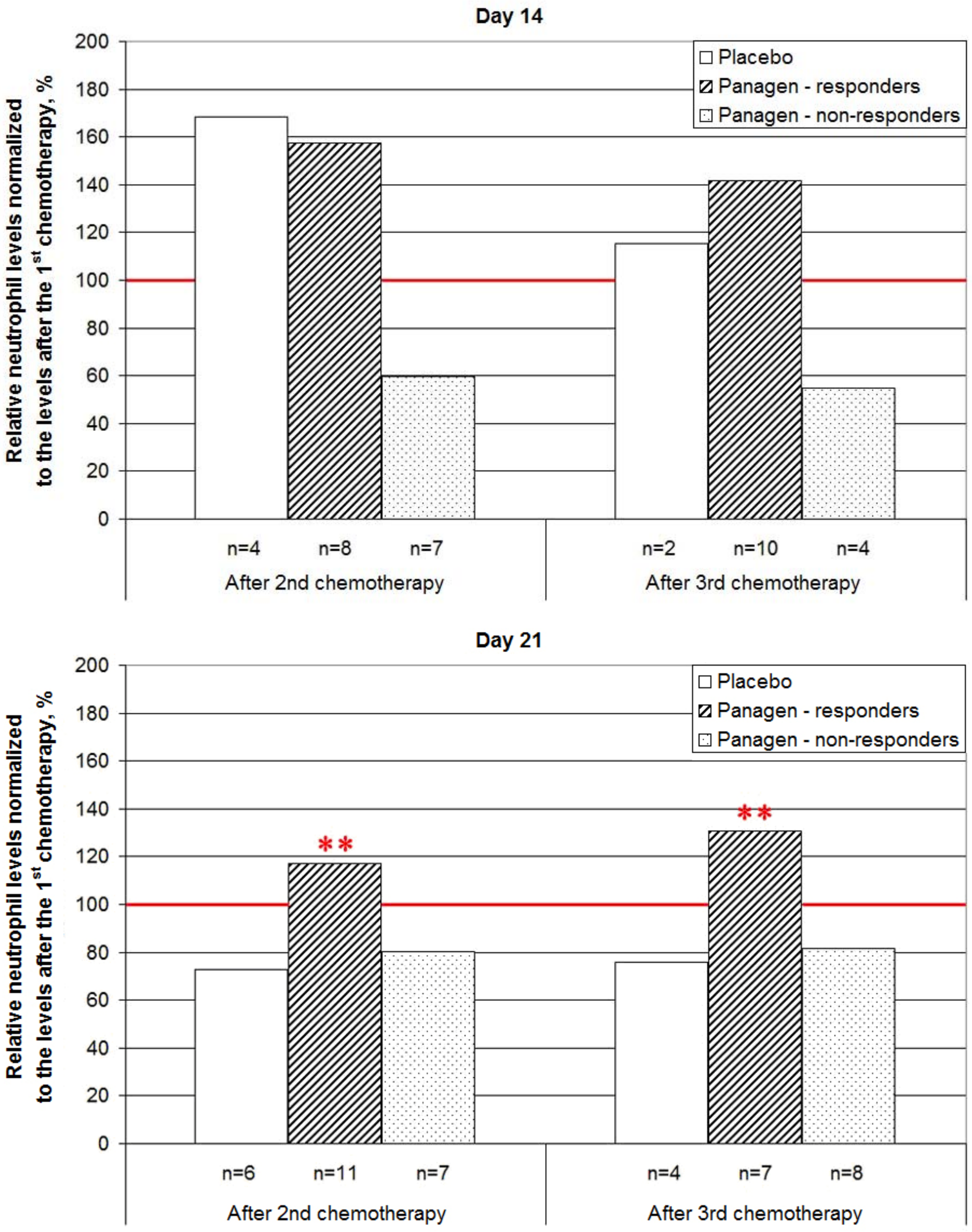

Figure 4. Relative neutrophil levels in the peripheral blood of patients on days 14 and 21 after the 2nd and 3rd CT normalized to the level after the 1st CT set to 100% (red line). Two red asterisks denote significant differences from the placebo group ($p<0.01$, Wilcoxon-Mann-Whitney test).

The occurrence of grade I-IV neutropenias on day 14 after cytostatic therapy throughout the three courses of CT increases from 0.8 to 1.0 in placebo-group patients. In Panagen patients, this parameter declines from 1.0 to 0.7 at the end of the study (**Table 5, Figure 5**).

Table 5. Occurrence of neutropenias in Panagen and placebo cohorts at control points throughout the three courses of CT.

	Neutro-penia grade	Day 0	Day 7			Day 14			Day 21		
			After 1st CT	After 2nd CT	After 3rd CT	After 1st CT	After 2nd CT	After 3rd CT	After 1st CT	After 2nd CT	After 3rd CT
Panagen	I	0	0.12	0.11	0.25	0.31	0.06	0.06	0.16	0.06	0.13
	II	0	0.06	0.11	0.06	0.19	0.35	0.24	0	0	0
	III	0	0.12	0	0	0.44	0.41	0.29	0	0	0
	IV	0	0	0	0	0.06	0.06	0.12	0	0	0
Placebo	I	0	0	0.29	0	0	0.20	0.67	0	0.14	0.20
	II	0	0	0	0	0.17	0.20	0.33	0	0	0
	III	0	0	0	0	0.33	0.20	0	0	0.14	0
	IV	0	0	0	0	0.33	0	0	0	0	0

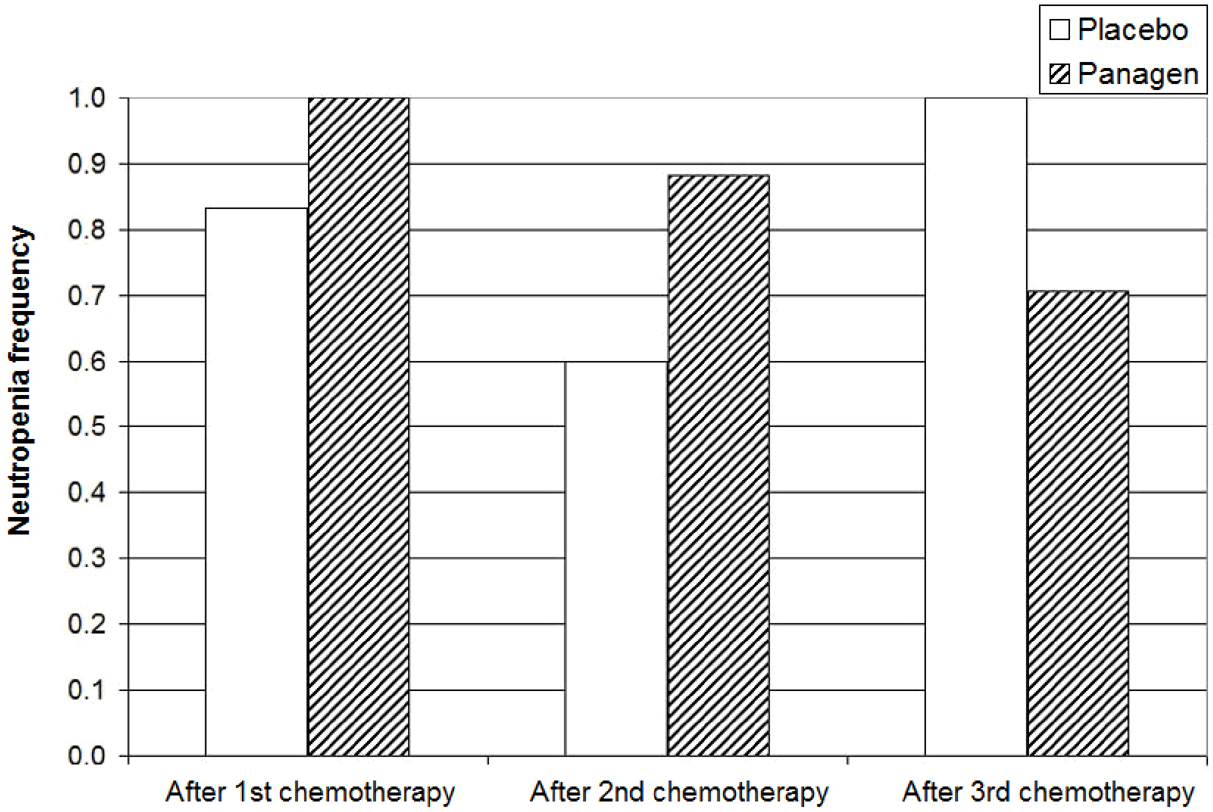

Figure 5. Occurrence of grade I-IV neutropenias in patients on day 14 of each of the three courses of CT.

Analysis of monocyte counts at control time points throughout the three courses of CT

Overall, this analysis supports a highly pronounced stimulation of monocyte lineage by the end of the second CT course (**Table 6, Figure 6**). By the end of the third CT, monocyte counts reach the levels found in the placebo-group patients.

Table 6. Monocyte counts (×10^9 cells/L) in Panagen- and placebo-group patients, as measured at different control time points after three courses of CT.

	Day 0	After the 1st CT			After the 2nd CT			After the 3rd CT		
		Day 7	Day 14	Day 21	Day 7	Day 14	Day 21	Day 7	Day 14	Day 21
**Panagen**										
02-20	0.376	0.014	0.014	0.175	0.072	0.210	0.616	0.130	0.224	0.630
02-21	0.316	0.074	0.320	0.219	0.061	0.432	0.850	0.164	0.437	0.213
02-22	0.200	0.039	0.054	0.310	0.129	0.341	0.438	0.098	0.420	
02-24	0.122	0.072	0.082	0.560	0.136	0.378	1.395	0.054	0.765	0.700
02-25	0.164	0.033	0.280	0.590	0.029	0.253	0.160	0.052	0.270	0.041
02-26	0.427	0.042	0.260	0.702	0.082	0.216	0.600	0.034	0.504	0.210
02-27	0.440	0.025	0.192	0.558	0.082	0.260		0.495	0.330	0.168
02-28	0.401	0.042	0.372	0.715	0.246	0.184	0.770		0.060	
02-29	0.160	0.108	0.286	0.650	0.068	0.154	0.540	0.105	0.140	0.219
02-30		0.037	0.986	0.086	0.099	0.092	0.640	0.023	0.018	
02-31	0.396	0.216	0.392	0.583	0.065		0.195	0.060		0.134
02-33	0.742	0.295	0.465	0.840	0.130	0.190	1.199	0.035	0.129	0.296
02-36	0.177	0.042	0.090		0.069	0.540	0.165			
02-39	0.039	0.021		0.106	0.022	0.060	0.204	0.084	0.144	0.082
02-40	0.048			0.324	0.063		0.156	0.028	0.056	0.184
02-42	0.051			0.160	0.026	0.024	0.074	0.170	0.090	0.264
02-43	0.180	0.037	0.078	0.440	0.112	0.084	0.948	0.305	0.164	0.264

02-44	0.231	0.045	0.175	0.828	0.038	0.147	0.066		0.168	0.300
02-45	0.084	0.100	0.238	0.092	0.021	0.042	0.094	0.022	0.160	0.046
n	18	17	16	18	19	17	18	16	17	15
Median value	**0.190**	**0.042**	**0.249**	**0.499**	**0.069**	**0.190**	**0.489**	**0.072**	**0.164**	**0.213**
Minimum	0.039	0.014	0.014	0.086	0.021	0.024	0.066	0.022	0.018	0.041
Maximum	0.742	0.295	0.986	0.840	0.246	0.540	1.395	0.495	0.765	0.700
Lower Quartile	0.122	0.037	0.086	0.175	0.038	0.092	0.160	0.035	0.129	0.134
Upper Quartile	0.396	0.074	0.346	0.650	0.112	0.260	0.770	0.147	0.330	0.296
**Placebo**										
02-23	0.150	0.053			0.048	0.140	0.152	0.078	0.216	0.216
02-32		0.123	0.416	0.768	0.045	0.120	0.232			1.110
02-34		0.093	0.300	0.305	0.027		0.096			0.102
02-35	0.256	0.123	0.380	0.518	0.099	0.102	0.118	0.034		0.320
02-37	0.258	0.045	0.325	0.792	0.056	0.420	0.410	0.058	0.050	0.372
02-38	0.640	0.053	0.102	0.158	0.064		0.108			
02-41	0.318	0.228	0.270	0.216	0.062	0.231	0.372		1.152	
n	5	7	6	6	7	5	7	3	3	5
Median value	**0.258**	**0.093**	**0.313**	**0.412**	**0.056**	**0.140**	**0.152**	**0.058**	**0.216**	**0.320**
Minimum	0.150	0.045	0.102	0.158	0.027	0.102	0.096	0.034	0.050	0.102
Maximum	0.640	0.228	0.416	0.792	0.099	0.420	0.410	0.078	1.152	1.110
Lower Quartile	0.256	0.053	0.270	0.216	0.045	0.120	0.108	0.034	0.050	0.216

Upper Quartile	0.318	0.123	0.380	0.768	0.064	0.231	0.372	0.078	1.152	0.372

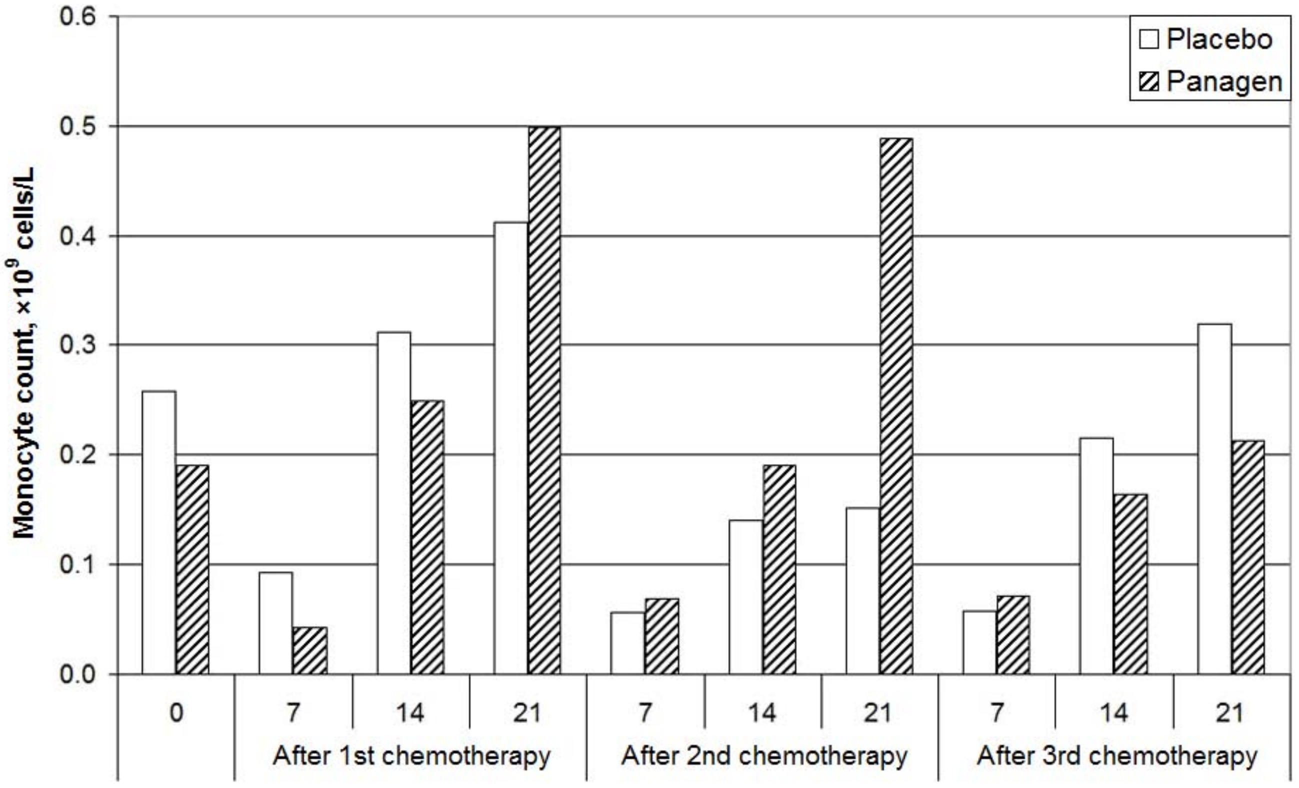

Figure 6. Absolute monocyte counts at the control time points on day 0 and on days 7, 14 and 21 after the CT courses. Median values in groups are shown.

We note that at the control time point on day 21 after the second CT course, the monocyte counts are significantly higher in Panagen-group patients, than in the placebo control ($p<0.09$, Wilcoxon-Mann-Whitney test). When Panagen-group patients are further subdivided into responders and non-responders, Panagen effects are discernible (much as with the entire Panagen-group sampling) at the control time points on days 14 and 21 after the second CT course in roughly half of the patients (47 and 59%, respectively) (**Table 7, Figure 7**). This analysis demonstrates significantly greater difference relatively to the placebo cohort ($p<0.05$ and $p<0.01$ for responders and non-responders, Wilcoxon-Mann-Whitney test).

Table 7. Relative monocyte counts (%) in Paganen- and placebo-group patients on days 14 and 21 after the 2nd and 3rd CT courses, normalized to the level after the 1st CT.

	Day 14		Day 21	
	After the 2nd CT	After the 3rd CT	After the 2nd CT	After the 3rd CT
**Panagen**				
02-20	1500.0	1600.0	352.0	360.0
02-21	135.0	136.6	388.1	97.3
02-22	631.5	777.8	141.3	
02-24	461.0	932.9	249.1	125.0
02-25	90.4	96.4	27.1	6.9
02-26	83.1	193.8	85.5	29.9
02-27	135.4	171.9		30.1
02-28	49.5	16.1	107.7	
02-29	53.8	49.0	83.1	33.7
02-30	9.3	1.8	744.2	
02-31			33.4	23.0
02-33	40.9	27.7	142.7	35.2
02-36	600.0			
02-39			192.5	77.4
02-40			48.1	56.8
02-42			46.3	165.0
02-43	107.7	210.3	215.5	60.0
02-44	84.0	96.0	8.0	36.2
02-45	17.6	67.2	102.2	50.0

Median value	**90.4**	**116.5**	**107.7**	**50.0**
Percent responders, %	47	50	59	20
Percent non-responders, %	53	50	41	80
Median value for responders	**461.0**	**210.3**	**204.0**	**165.0**
Median value for non-responders	51.7	49.0	46.3	35.7
Combined percentage of responders, %	**53**		**61**	
**Placebo**				
02-32	28.8		30.2	144.5
02-34			31.5	33.4
02-35	26.8		22.8	61.8
02-37	129.2	15.4	51.8	47.0
02-38			68.4	
02-41	85.6	426.7	172.2	
Median value	**57.2**	**221.0**	**41.6**	**54.4**

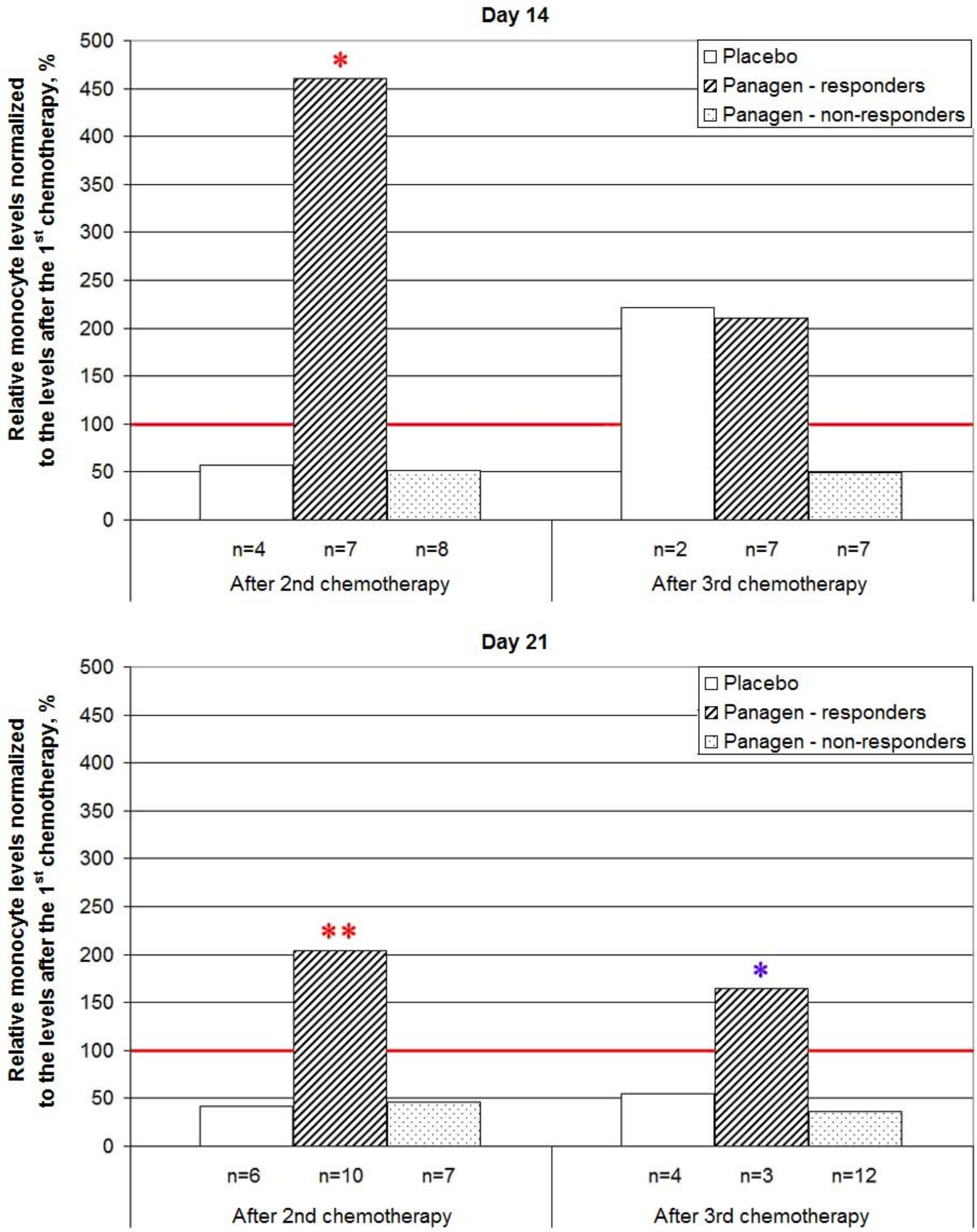

Figure 7. Relative monocyte levels in the peripheral blood of patients on day 14 and 21 after the 2nd and 3rd CT normalized to the 1st CT level set to 100% (red line). Median values that are significantly different from placebo group (Wilcoxon-Mann-Whitney test) are shown as blue asterisk ($p<0.08$), red asterisk ($p<0.05$) and two red asterisks ($p<0.01$).

Analysis of lymphocyte cell counts at the control time points throughout three CT courses

Differences in lymphocyte cell counts between placebo- and Panagen-group patients are noteworthy. In placebo group, the values progressively decline as we move from one cycle to another, and in the very last control time point (day 21 after the third CT) absolute lymphocyte counts are indistinguishable from those in Panagen group. Yet, at the starting, pre-therapy, time point lymphocyte cell counts in placebo-group patients exceed those in the Panagen group, which points to the fact that the sampling of placebo-group patients happened to be healthier. In the Panagen group, lymphocyte counts stay essentially unaltered until the last control time point, which support a protective activity of Panagen on this cell lineage (**Table 8, Figure 8**).

Table 8. Absolute lymphocyte counts (×10^9 cells/L) in patients from Panagen and placebo groups as measured at different control points throughout control points of three CT courses

	Day 0	After the 1st CT			After the 2nd CT			After the 3rd CT		
		Day 7	Day 14	Day 21	Day 7	Day 14	Day 21	Day 7	Day 14	Day 21
**Panagen**										
02-20	0.56	0.39	0.56	1.16	0.25	0.54	1.41	0.52	0.59	0.42
02-21	2.05	1.26	1.18	1.90	1.59	1.30	2.04	1.39	1.38	1.85
02-22	1.80	1.44	0.81	1.55	1.20	1.71	2.48	1.91	1.72	
02-24	2.56	2.02	2.38	2.48	1.43	2.52	3.16	1.94	1.53	0.70
02-25	1.60	0.76	0.17	1.18	0.55	0.61	0.60	0.68	0.81	1.15
02-26	1.65	1.05	1.30	2.92	1.80	1.58	2.10	0.75	2.31	2.59
02-27	2.29	0.65	1.27	1.92	0.94	1.04		1.98	0.86	1.34
02-28	2.21	1.85	2.05	2.80	1.39	1.56	2.03		0.88	
02-29	2.24	0.76	1.59	2.60	0.85	0.90	1.62	0.70	0.70	2.48
02-30		1.67	1.33	2.41	1.19	0.94	1.60	1.24	0.54	
02-31	1.36	1.12	1.37	1.06	0.59		1.56	0.84		2.55
02-33	5.09	2.48	1.67	3.26	1.56	1.37	2.51	1.23	2.11	1.85
02-36	2.42	1.93	1.32		3.17	1.13	2.04			
02-39	1.09	0.63		1.27	0.33	0.33	1.38	0.84	0.49	0.74
02-40	1.44			1.73	0.79		1.05	0.92	0.73	1.56
02-42	1.48	0.65	0.80	1.34	0.88	0.36	0.96	0.95	1.56	1.42

02-43	2.28	0.89	0.83	1.94	1.18	0.98	2.69	1.34	1.89	1.39
02-44	3.16	1.53	1.68	2.39	1.33	0.63	2.24		1.26	1.44
02-45	1.43	0.92	0.73	1.56	0.63	0.76	1.27	0.44	0.61	0.74
n	18	18	17	18	19	17	18	16	17	15
Median	**1.93**	**1.08**	**1.30**	**1.91**	**1.18**	**0.98**	**1.83**	**0.94**	**0.88**	**1.42**
Minimum	0.56	0.39	0.17	1.06	0.25	0.33	0.60	0.44	0.49	0.42
Maximum	5.09	2.48	2.38	3.26	3.17	2.52	3.16	1.98	2.31	2.59
Lower Quartile	1.44	0.76	0.81	1.34	0.63	0.63	1.38	0.72	0.70	0.74
Upper Quartile	2.29	1.67	1.59	2.48	1.43	1.37	2.24	1.37	1.56	1.85
**Placebo**										
02-23	0.90	1.06			1.01	1.23	1.98	1.25	0.62	1.07
02-32		1.27	2.05	2.02	0.81	0.70	1.68			1.70
02-34		0.50	1.02	2.32	1.00	0.56	0.69			0.92
02-35	1.79	1.23	1.34	2.96	1.42	0.95	1.77	0.88		2.56
02-37	2.92	1.80	2.15	2.29	1.12	1.34	2.46	2.09	1.18	3.91
02-38	4.64	1.91	1.84	2.92	2.18		1.78			
02-41	2.76	1.20	1.43	2.16	2.23	1.49	2.79		1.68	
n	5	7	6	6	7	6	7	3	3	5
Median	**2.76**	**1.23**	**1.63**	**2.30**	**1.12**	**1.09**	**1.78**	**1.25**	**1.18**	**1.70**
Minimum	0.90	0.50	1.02	2.02	0.81	0.56	0.69	0.88	0.62	0.92
Maximum	4.64	1.91	2.15	2.96	2.23	1.49	2.79	2.09	1.68	3.91

Lower Quartile	1.79	1.06	1.34	2.16	1.00	0.70	1.68	0.88	0.62	1.07
Upper Quartile	2.92	1.80	2.05	2.92	2.18	1.34	2.46	2.09	1.68	2.56

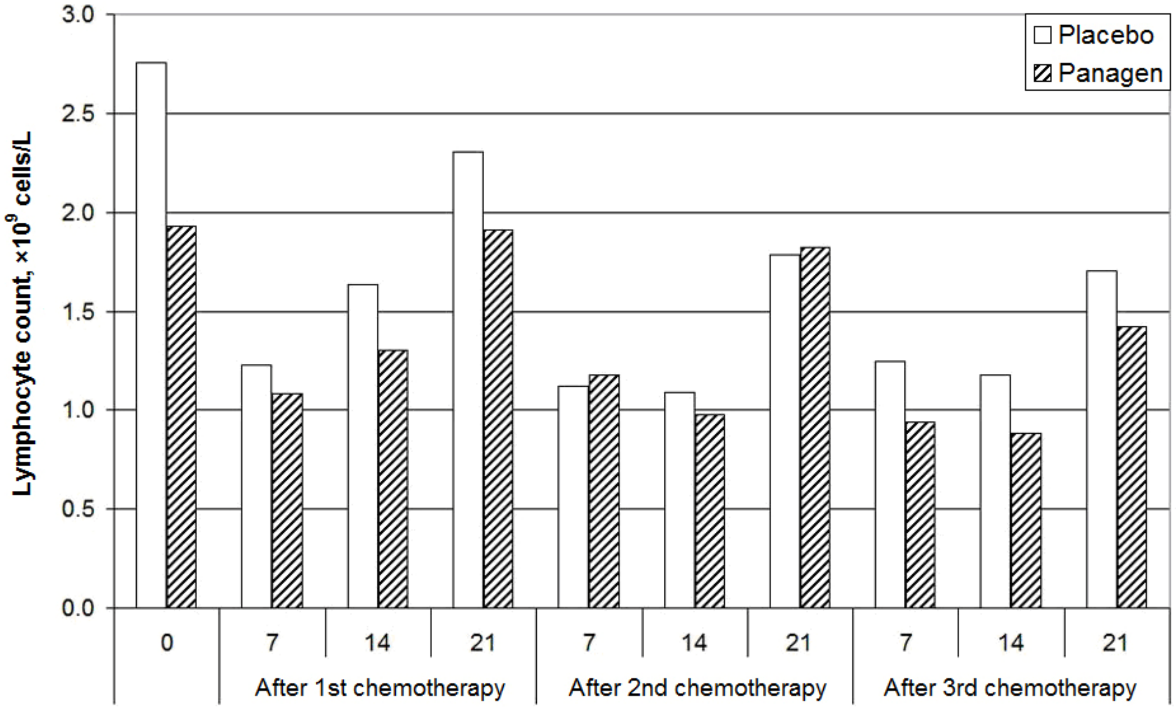

Figure 8. Absolute lymphocyte counts at the starting point on day 0 and at control points on days 7, 14 and 21 after each of the three CT courses. Median values in groups are plotted.

When Panagen-group patients were further subdivided into responders and non-responders, the differences between the groups become significant, with the percentage of responding patients at different control time points ranging from 13 to 53% of patients. It must be noted, that patients tend to fluctuate between responding and non-responding sub-groups. This fact shows highly individualized timing of stimulating activity of Panagen on lymphocytic progenitor lineage in different patients that took part in the study (**Table 9, Figure 9**).

Table 9. Relative levels of peripheral-blood lymphocytes (%) in patients samples on days 14 and 21 after the 2nd and 3rd CT courses, normalized to the level after the 1st CT.

	Day 14		Day 21	
	After the 2nd CT	After the 3rd CT	After the 2nd CT	After the 3rd CT
**Panagen**				
02-20	96.4	105.0	121.9	36.4
02-21	109.5	116.6	107.5	97.3
02-22	210.5	212.6	160.1	
02-24	106.0	64.3	127.5	28.2
02-25	360.1	482.1	50.8	97.3
02-26	121.8	177.7	72.0	88.8
02-27	81.8	67.5		69.9
02-28	76.4	43.0	72.6	
02-29	56.9	44.1	62.3	95.5
02-30	71.1	40.7	66.4	
02-31			147.2	240.2
02-33	81.7	125.9	77.0	56.8
02-36	85.9			
02-39			108.3	58.0
02-40			60.9	90.5
02-42	45.3	196.2	71.6	105.6
02-43	117.8	226.7	138.7	71.6
02-44	37.6	75.2	93.8	60.2
02-45	103.8	83.5	81.1	47.1
Median value	**91.2**	**105.0**	**81.1**	**71.6**
Percent	44	53	41	13

responders, %				
Percent non-responders, %	56	47	59	87
Median value for responders	**117.8**	**186.9**	**127.5**	**172.9**
Median value for non-responders	76.4	64.3	71.8	69.9
Combined percentage of responders, %	**63**		**44**	
**Placebo**				
02-32	34.2		83.4	84.4
02-34	54.5		29.7	39.6
02-35	71.0		59.8	86.5
02-37	62.7	54.8	107.5	170.7
02-38			61.0	
02-41	103.8	117.4	129.2	
Median value	**62.7**	**86.1**	**72.2**	**85.5**

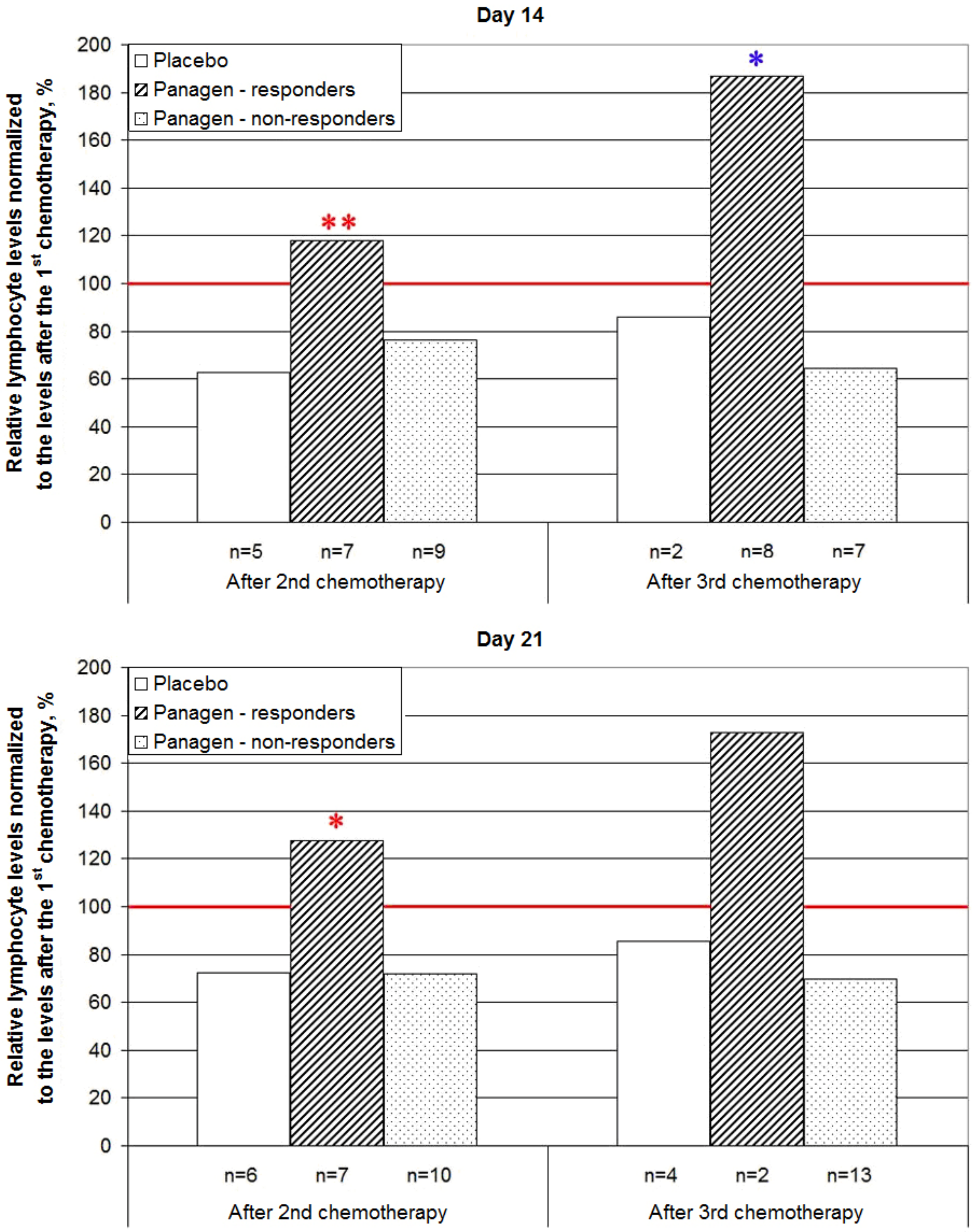

Figure 9. Relative levels of peripheral-blood lymphocytes in patients on days 14 and 21 of the 2nd and 3rd CT normalized to the levels found after the 1st CT set to 100% (red line). Median values showing significant difference from the placebo-group values are marked as a blue asterisk(p<0.12), red asterisk (p<0.05) or two red asterisks (p<0.01).

Panagen activity towards erythroid lineage. Erythropoiesis and effects on megakaryocyte cell lineage.

We evaluated the Panagen activity on several parameters that are informative of the erythroid lineage state throughout the CT courses (**Tables 10-14**).

RBC counts below the normal range were observed in 53% of Panagen-group patients at some point of the CT. In contrast, among placebo-group patients this was observed in only 1 out of 7 patients, which translates into 14% (**Table 10**).

In terms of Hb levels, 37% of Panagen-group patients had low Hb, whereas none of the placebo-group patients had Hb levels below the norm (**Table 11**).

We observed declining platelet counts in 68% Panagen-group patients and in 71% placebo-group patients (**Table 12**). However, by day 21 after each CT course, we noted increased platelet counts in both patient groups, and sometimes in Panagen-group patients platelet counts were even higher than the normal range. Importantly, on day 21 of each CT course Panagen-group patients displayed platelet counts higher than the starting levels ($p<0.05$, paired Wilcoxon test).

No significant changes in MCHC were found during the study. Only 2 out of 19 Panagen-group patients (11%) had MCHC above the normal range, which was likely an individual feature of those patients (**Table 13**).

Throughout the study, low Hct was observed in 79% of Panagen-group and in 57% of placebo-group (**Table 14**).

As it follows from our analysis, use of Panagen results in higher platelet counts on day 21 after the CT courses.

Table 10. RBC counts in the blood of patients from Panagen and placebo groups at different time points of the CT. Normal RBC range is 3.8 – 5.1 $\times 10^{12}$ cells/L.

	0	Days after the 1st CT			Days after the 2nd CT			Days after the 3rd CT		
		7	14	21	7	14	21	7	14	21
**Panagen**										
02-20		4.61	4.56	4.78	4.52	4.52	4.94	4.51	4.39	4.61
02-21	4.60	4.51	4.10	4.34	4.44	4.09	4.02	4.22	4.13	4.29
02-22		4.03	3.82	4.15	3.56	3.84	4.00	3.68	3.76	
02-24	4.55	4.17	3.79	3.95	4.17	3.87	4.24	3.96	3.98	4.23
02-25	4.59	4.10	4.08	4.41	3.71	3.53	3.69	3.45	3.58	3.84
02-26	3.81	3.76	3.52	3.97	3.87	3.41	4.03	3.46	3.45	3.34

02-27	4.49	4.03	4.00	4.27	4.07	3.90		4.14	3.88	3.96
02-28	4.82	4.56	4.44	4.36	4.43	4.22	4.33		3.89	
02-29	4.46	4.36	3.90	4.18	3.72	3.71	3.80	3.66	3.80	3.58
02-30		3.91	3.96	3.98	3.80	3.80	4.08	3.40	3.69	
02-31	4.27	3.97	3.39	4.10	3.75		3.63	3.40		3.55
02-33	4.60	4.54	4.25	4.51	4.55	4.33	4.70	4.07	4.30	3.87
02-36	5.18	5.06	4.79		4.77		4.40			
02-39	3.74	3.74	3.25	3.47	3.29	3.90	3.44	3.50	3.35	3.31
02-40	4.16			4.13	3.90		4.07	3.60	3.73	3.48
02-42	4.09	3.58	3.49	3.99	3.58	3.49	3.80	3.70	3.64	4.11
02-43	4.70	4.18	4.17	4.47	4.28	3.90	4.26	4.25	4.21	4.14
02-44	4.31	3.73	3.90	4.49	3.88	3.75	4.09	3.40		3.90
02-45	4.48	4.42	4.40	4.53	4.07	4.33	4.45	3.83	3.96	4.38
Median value	**4.49**	**4.14**	**3.98**	**4.23**	**3.90**	**3.89**	**4.08**	**3.68**	**3.84**	**3.90**
**Placebo**										
02-23	4.52	4.87			4.43	4.39	4.81	4.75	4.79	4.33
02-32		4.28	4.24	4.44	4.00	3.87	4.30			4.14
02-34		4.26	4.35	4.30	3.99	3.69	3.78	3.30		3.69
02-35	5.02	4.66	4.70	4.55	4.24	4.01	4.70	3.95		4.30
02-37	4.55	3.95	4.49	4.93	4.37	4.11	4.36	3.94	4.06	4.16
02-38	4.74	4.13	4.11	4.40	3.98		4.15			
02-41	5.37	5.26	4.80	4.88	4.88	4.73	4.46		3.80	
Median value	**4.74**	**4.28**	**4.42**	**4.50**	**4.24**	**4.06**	**4.36**	**3.95**	**4.06**	**4.16**

Table 11. Hb levels in the blood of patients from Panagen and placebo cohorts at different control time points of the CT. Normal Hb range is 120-155 g/L.

	0	Days after the 1st CT			Days after the 2nd CT			Days after the 3rd CT		
		7	14	21	7	14	21	7	14	21
**Panagen**										
02-20		132	129	133	127	130	139	128	132	139
02-21	139	132	133	125	127	120	122	120	124	128
02-22		127	119	129	111	116	124	114	116	
02-24	134	119	111	114	119	109	124	119	119	125
02-25	143	127	128	140	118	113	123	109	112	126

02-26	133	132	128	136	133	127	142	121	117	113
02-27	134	124	133	135	131	125		136	121	131
02-28	144	142	131	133	138	135	130		125	
02-29	121	131	119	127	112	116	120	118	110	116
02-30		118	121	125	117	117	120	103	114	
02-31	122	111	100	119	110		109	104		111
02-33	133	131	127	135	134	129	133	122	132	120
02-36	115	122	118		125		118			
02-39	119	117	102	109	104	129	109	113	105	104
02-40	129			131	119		127	113	118	104
02-42	121	121	103	110	105	101	110	101	107	115
02-43	145	132	123	141	132	120	132	142	126	130
02-44	131	112	119	130	121	114	128		110	125
02-45	148	134	139	143	131	139	141	122	127	141
Median value	**133**	**127**	**122**	**131**	**121**	**120**	**124**	**119**	**118**	**125**
**Placebo**										
02-23	131	124			129	120	137	137	144	128
02-32		131	128	131	125	121	121			131
02-34		138	133	140	130	120	125	112		124
02-35	146	142	136	140	133	124	140	122		134
02-37	141	121	134	157	132	126	135	123	120	130
02-38	133	123	123	130	120		127			
02-41	150	146	130	134	140	138	131		124	
Median value	**141**	**131**	**132**	**137**	**130**	**123**	**131**	**123**	**124**	**130**

Table 12. Platelet counts in the blood samples of patients from Panagen and placebo cohorts measured at different control time points of the CT. Normal range is 150 – 400x10^9 cells/L

	0	Days after the 1st CT			Days after the 2nd CT			Days after the 3rd CT		
		7	14	21	7	14	21	7	14	21
**Panagen**										
02-20		152	173	341	225	139	236	242	162	310
02-21		101	200	205	157	128	245	110	147	219
02-22		162	192	356	208	172	245	287	199	
02-24	236	201	232	364	185	259	361	235	186	200

02-25	213	128	138	225	90	128	347	100	132	223
02-26	241	213	216	420	224	173	302	197	104	391
02-27	265	169	384	280	186	179		199	276	209
02-28	283	192	213	254	206	210	267		279	
02-29	228	201	141	452	211	96	341	204		192
02-30		145	175	476	252	155	310	149	101	
02-31	225	241	340	344	268		450	200		379
02-33	277	170	145	299	141	165	421	159	143	271
02-36	196	115	196		155		257			
02-39	125	96	131	226	154	118	208	172	123	221
02-40	139			204	181		234	118	167	281
02-42	166	223	192	349	177	125	386	235	136	219
02-43	150	107	141	224	134	151	177	88	200	225
02-44	253	173	218	333	220	194	354			270
02-45	141	112	306	281	177	192	250	167	239	310
Median value	**225**	**166**	**194**	**316**	**185**	**160**	**285**	**185**	**162**	**225**
**Placebo**										
02-23	308	349			267	137	247	263	158	270
02-32		157	283	341	241	167				332
02-34		109	233		115	153	185	90		195
02-35	130	127	207	393	119	218		110		297
02-37	299	313	257	215	206	251	205	222	168	297
02-38	122	169	273	385	279		322			
02-41	193	119	144	131	178	145	230		203	
Median value	**193**	**157**	**245**	**341**	**206**	**160**	**230**	**166**	**168**	**297**

Table 13. MCHC in Panagen- and placebo-group patients' blood samples at different control time points of the CT. Normal MCHC range is 27-34 pg.

	0	Days after the 1st CT			Days after the 2nd CT			Days after the 3rd CT		
		7	14	21	7	14	21	7	14	21
**Panagen**										
02-20		28.6	28.3	27.8	28.1	28.8	28.1	28.4	30.1	30.2
02-21		29.3		28.8	28.6	29.3	30.3	28.4	30.0	29.8
02-22		31.5	31.2	31.1	31.2	30.2	31.0	31.0	30.9	

02-24	29.5	28.5	29.3	28.9		28.2	29.2	30.1	29.8	29.6
02-25	31.2	31.0	31.4	31.7	31.8	32.0	33.3	31.6	31.3	32.8
02-26	34.9	35.1	36.4	34.2	34.4		35.2	35.0	33.8	33.8
02-27	29.8	30.8		31.6	32.2	32.1			31.2	33.1
02-28	29.8	31.3	29.5	30.5	31.2	32.0	30.0		32.1	
02-29	27.1	30.1	30.5	30.4	30.1	31.3	31.6	32.2		32.4
02-30		30.2	30.6	31.4			29.4		30.9	
02-31		27.9	29.5	29.0	29.3		30.0	30.6		31.3
02-33	28.9	28.9	29.9	29.9	29.5	29.8	28.3	30.0	30.7	31.0
02-36	22.2	24.1	37.3		26.2					
02-39	31.8	31.3	31.4	31.4	31.6	33.1	31.7	32.3	31.3	31.4
02-40	31.0				30.5		31.2	31.4	31.6	29.9
02-42	29.6	33.8	29.5	27.6	29.3	28.9	28.9	27.3	29.4	28.0
02-43		31.6	29.5	31.5	30.8	30.8	31.0	33.4	29.9	31.4
02-44	30.4	30.0	30.5	29.0	31.2	30.4	31.3			32.1
02-45	33.0	30.3	31.6	31.6	32.2	32.1	31.7	31.9	32.1	32.2
Median value	**29.8**	**30.3**	**30.5**	**30.5**	**30.8**	**30.6**	**31.0**	**31.2**	**30.9**	**31.4**
**Placebo**										
02-23	29				29.1	27.3	28.5	28.8	30.1	29.6
02-32		30.6	30.2	29.5	31.3	31.3				31.6
02-34		32.4	30.5		32.6	32.5	33.1			33.6
02-35	29.1	30.5	28.8		31.4	30.9		30.9		31.2
02-37	31.0	30.6	29.8	31.8	30.2	30.7	31.0	31.2	29.6	31.3
02-38	28.1	29.8	29.9	29.5	30.2					
02-41	27.9	27.8	27.1	27.5	28.7	29.2	29.4			
Median value	**29.0**	**30.6**	**29.9**	**29.5**	**30.2**	**30.8**	**30.2**	**30.9**	**29.9**	**31.3**

Table 14. Hct values in Panagen- and placebo-group patients' blood samples at different control time points of the CT. Normal Hct level is 35-45%.

	0	Days after the 1st CT			Days after the 2nd CT			Days after the 3rd CT		
		7	14	21	7	14	21	7	14	21
**Panagen**										
02-20		36.9	37.6	41.4	38.6	37.4	43.9	39.3	37.0	39.4
02-21		37.0		36.7	38.5	34.0	36.4	36.8	34.6	36.3

02-22		35.5	34.3	37.6	33.2	37.4	36.0	34.8	35.9	
02-24	38.6	35.5	32.5	33.6		34.7	35.8	33.7	35.8	36.6
02-25	43.1	38.3	35.9	39.1	32.7	31.2	33.1	32.2	33.3	37.0
02-26	39.1	38.9	34.8	41.9	39.3		38.7	34.6	34.2	33.6
02-27	41.6	35.7		39.0	36.6	35.3		37.7	36.3	37.4
02-28	42.8	40.2	42.2	39.0	39.1	36.9	40.7		37.4	
02-29	38.7	38.6	33.2	38.4	33.6	31.7	32.7	31.6		32.8
02-30		33.2	33.8	35.4		32.0	37.0		32.9	
02-31	39.8	35.3	27.9	34.1	31.2		30.8	30.7		32.0
02-33	41.2	40.0	36.0	38.4	38.7	36.7	40.1	35.7	38.4	34.4
02-36	39.8	39.0	37.3		36.9					
02-39	33.9	33.9	29.0	31.2	29.4	34.9	30.7	31.0	30.1	30.1
02-40	37.3				32.5		36.2	32.0	33.9	25.6
02-42	38.1	31.7	30.5	35.1	30.9	29.8	32.5	31.7	30.9	35.6
02-43		39.4	36.5	39.0	36.7	33.9	37.1	35.9	36.8	36.5
02-44	38.4	33.3	34.7	41.0	34.8	33.9	36.7			36.1
02-45	39.4	39.2	38.7	39.9	35.8	38.3	39.3	33.9	34.5	38.9
Median value	**39.3**	**37.0**	**34.8**	**38.4**	**35.8**	**34.7**	**36.4**	**33.9**	**34.6**	**36.1**
**Placebo**										
02-23	38.0				37.5	37.6	40.9	39.8	39.7	37.0
02-32		37.5	37.1	39.4	35.4	34.4				37.5
02-34		38.2	39.1		37.0	34.4	35.6			37.8
02-35	45.4	40.4	39.8		37.8	35.9		38.3		41.8
02-37	40.9	35.4	40.7	43.3	38.5	36.0	38.8	35.0	33.8	36.7
02-38	39.6	35.7	35.5	37.9	34.0					
02-41	44.1	42.9	39.3	40.1	39.9	39.3	36.6			
Median value	**40.9**	**37.9**	**39.2**	**39.8**	**37.5**	**36.0**	**37.7**	**38.3**	**36.8**	**37.5**

Blood biochemistry parameters measured at different control time points of the CT

To monitor the dynamics of blood biochemistry parameters, blood was collected before the study and on day 21 of each CT course.

The results are summarized in **Tables 15-25.** Changes in biochemical parameters relatively to their normal ranges were assessed at the end of the study (**Table 26**).

Table 15. ALT levels (U/L) in peripheral blood of patients from Panagen and placebo groups, at starting time point on day 0 and at different control time points. Normal ALT range is 10-44 U/L

	Day 0	Day 21		
		After the 1st CT	After the 2nd CT	After the 3rd CT
**Panagen**				
02-20	28	29	22	22
02-21	13	14	16	17
02-22		15	23	
02-24	13	12	11	
02-25	14	20	13	24
02-26	22		15	13
02-27	27	30	27	42
02-28	21	21	28	16
02-29	14	16	17	14
02-30	11	21	45	47
02-31	12	13	12	10
02-33	24	45	32	81
02-36	25	21	22	29
02-39	33		24	
02-40	46	50	54	
02-42	15			17
02-43		25	61	30
02-44	24	36	22	17
02-45	46	28	42	35
Median	**22**	**21**	**23**	**22**

Placebo				
02-23	24		100	61
02-32		38		37
02-34	16	24	11	21
02-35	11	12	12	16
02-37	16		284	
02-41	23	24	19	16
Median	**16**	**24**	**19**	**21**

Table 16. AST levels (U/L) in peripheral blood of patients from Panagen and placebo groups, at starting time point on day 0 and at different control time points. Normal AST range is 10-34 U/L.

	Day 0	Day 21		
		After the 1st CT	After the 2nd CT	After the 3rd CT
**Panagen**				
02-20	27	33	31	27
02-21	17	21	23	21
02-22		15	26	
02-24	12	21	10	
02-25	19	21	21	26
02-26	24		18	22
02-27	26	26	21	29
02-28	17		32	14
02-29	26	23	33	27
02-30	15	29	16	43
02-31	18		14	17
02-33	29	29	27	57
02-36	32	27	25	33
02-39	22		23	
02-40	29	30	29	
02-42	16			18
02-43		14	39	25
02-44	25	23	20	15
02-45	33	27	33	23
Median	**24**	**25**	**24**	**25**

**Placebo**				
02-23	22		83	55
02-32		35		28
02-34	20	22	20	29
02-35	14	17	18	21
02-37	17		129	
02-41	24	22	20	15
Median	**20**	**22**	**20**	**28**

Table 17. Alkaline phosphatase levels (U/L) in peripheral blood of patients from Panagen and placebo groups, at the starting time point on day 0 and at different control time points. Normal alkaline phosphatase range is 32-126 U/L.

	Day 0	Day 21		
		After the 1st CT	After the 2nd CT	After the 3rd CT
**Panagen**				
02-20	92	79	81	72
02-21	71	61	68	67
02-22		55	58	
02-24		72		
02-25	6	57	58	48
02-26	70		55	53
02-27	89	81	70	91
02-28	74		161	
02-29	64	61	17	58
02-30	55	39	184	72
02-31	106	93	75	100
02-33	61	85	76	
02-36	52			74
02-39	66		68	
02-40	73	68	62	
02-42	55			60
02-43			64	67
02-44	103	69	83	86

02-45	105	88	80	82
Median	**71**	**69**	**69**	**72**
**Placebo**				
02-23	71		84	96
02-32		99		
02-34	74	144	73	
02-35	65	35	68	
02-37	180		429	
02-41	85	78	76	84
Median	**74**	**89**	**76**	**90**

Table 18. Total bilirubin levels (mkM/L) in peripheral blood of patients from Panagen and placebo groups, at starting time point on day 0 and at different control time points. Normal total bilirubin range is 3.4-17 mkM/L.

	Day 0	Day 21		
		After the 1st CT	After the 2nd CT	After the 3rd CT
**Panagen**				
02-20	10.0	4.8	5.1	
02-21	22.1	5.1	6.0	
02-22		9.8	5.2	
02-24	7.4	4.8	9.6	
02-25	6.0	6.1	7.6	5.0
02-26	6.7		6.4	6.0
02-27	8.5	6.8	4.7	
02-28	7.7		10.1	
02-29	7.8	6.0	5.6	
02-30	14.2	6.0	14.6	9.9
02-31	6.0	21.8	4.0	4.8
02-33	6.3	5.0	5.4	5.0
02-36	6.7	5.0		6.0
02-39	7.4		8.1	
02-40	7.1	5.0	6.3	
02-42	6.9			7.0

02-43		8.9	7.9	7.8
02-44	7.2	5.6	5.4	6.7
02-45	6.0	7.0	6.4	6.9
Median	**7.2**	**6.0**	**6.3**	**6.4**
**Placebo**				
02-23	6.6		6.3	6.6
02-32		8.5		6.7
02-34	6.7	10.0	5.5	6.4
02-35	7.9	7.8	7.2	7.2
02-37	8.9		9.7	
02-41	9.4	6.4	6.1	5.7
Median	**7.9**	**8.2**	**6.3**	**6.6**

Table 19. Creatinine levels (mkM/L) in the blood samples of patients from Panagen and placebo groups, as measured at the starting time point and at different control time points throughout the study. Normal range is 44-97 mkM/L.

	Day 0	Day 21		
		After the 1st CT	After the 2nd CT	After the 3rd CT
**Panagen**				
02-20	60	72	66	63
02-21	44	55	51	49
02-22		54	79	
02-24	72	53	72	
02-25	60	62	37	53
02-26	6		35	57
02-27	49	56	49	55
02-28	50	69	84	53
02-29	49	62	56	50
02-30	55	38	66	52
02-31	51	46	39	46
02-33	61	58	62	52
02-36	56	50	63	44
02-39	66		79	

02-40	58	53	50	
02-42	48			63
02-43		69	76	73
02-44	68	70	69	58
02-45	61	61	62	54
Median	**56**	**57**	**62**	**53**
**Placebo**				
02-23	52		68	62
02-32		61		55
02-34	40	86	34	55
02-35	51	49	53	67
02-37	84		77	
02-41	7	66	65	57
Median	**51**	**64**	**65**	**57**

Table 20. Urea (mM/L) in blood samples of patients from Panagen and placebo groups, measured at the starting time point and at different control time points throughout the study. Normal range is 2.5-8.3 mM/L.

	Day 0	Day 21		
		After the 1st CT	After the 2nd CT	After the 3rd CT
**Panagen**				
02-20	3.9	4.3	4.3	5.6
02-21	2.2	2.1	2.8	4.4
02-22		2.4	7.1	
02-24	5.4	3.0	6.2	
02-25	2.6	3.7	4.1	4.6
02-26	3.5		3.7	3.6
02-27	3.5	3.5	3.8	
02-28	4.9	3.3	7.2	3.2
02-29	3.7	2.7	2.8	
02-30		4.9	6.0	3.2
02-31	2.6	1.8	2.1	1.8
02-33	4.0	4.3	3.3	3.8

02-36	4.5	4.5	4.4	4.8
02-39	5.9		6.3	
02-40	8.1	7.4	6.5	
02-42	3.7			4.2
02-43		3.1	3.9	4.0
02-44	4.7	3.8	6.0	3.7
02-45	6.8	6.6	3.9	4.0
Median	**4.0**	**3.6**	**4.2**	**4.0**
**Placebo**				
02-23	5.8		2.8	3.3
02-32		6.0		3.4
02-34	4.6	4.2	2.9	4.3
02-35	4.9	4.3	4.2	6.3
02-37	6.9		5.8	
02-41	3.9	5.4	5.6	5.9
Median	**4.9**	**4.9**	**4.2**	**4.3**

Table 21. Blood glucose levels (mM/L) in Panagen-and placebo-group patients on day 0 and at different control time points. Normal range is 3.3-5.5 mM/L.

	Day 0	Day 21		
		After the 1st CT	After the 2nd CT	After the 3rd CT
**Panagen**				
02-20	5.8	5.2	5.4	5.6
02-21	6.0	5.5	5.8	6.2
02-22		4.5	5.3	
02-24		5.3		
02-25	4.6	4.8	6.0	3.8
02-26	5.1		5.4	
02-27	4.1	4.4	5.9	4.4
02-28	5.5	6.1		6.3
02-29	4.9	5.3	4.9	3.8
02-30	4.7	5.3	4.4	4.6
02-31	4.3	4.9	4.0	5.1

02-33	4.9	5.4		
02-36				4.7
02-39	5.8		6.2	
02-40	5.9	5.9	6.9	
02-42	4.5			5.0
02-43			5.5	5.0
02-44	5.8	6.0	6.0	4.8
02-45	5.3	5.2	4.6	4.5
Median	**5.1**	**5.3**	**5.5**	**4.8**
**Placebo**				
02-23	6.4		6.0	5.1
02-32		6.6		
02-34	3.7		5.2	5.1
02-35	4.4	3.9	6.0	4.8
02-41	8.4	8.1	7.9	7.1
Median	**5.4**	**6.6**	**6.0**	**5.1**

Table 22. Blood cholesterol levels (mM/L) in Panagen-and placebo-group patients on day 0 and at different control time points. Normal range is 3.4-5.2 mM/L.

	Day 0	Day 21		
		After the 1st CT	After the 2nd CT	After the 3rd CT
**Panagen**				
02-20	3.9	4.3	4.3	5.6
02-21	2.2	2.1	2.8	4.4
02-22		2.4	7.1	
02-24	5.4	3.0	6.2	
02-25	2.6	3.7	4.1	4.6
02-26	3.5		3.7	3.6
02-27	3.5	3.5	3.8	
02-28	4.9	3.3	7.2	3.2
02-29	3.7	2.7	2.8	
02-30		4.9	6.0	3.2
02-31	2.6	1.8	2.1	1.8

02-33	4.0	4.3	3.3	3.8
02-36	4.5	4.5	4.4	4.8
02-39	5.9		6.3	
02-40	8.1	7.4	6.5	
02-42	3.7			4.2
02-43		3.1	3.9	4.0
02-44	4.7	3.8	6.0	3.7
02-45	6.8	6.6	3.9	4.0
Median	**4.0**	**3.6**	**4.2**	**4.0**
**Placebo**				
02-23	5.8		2.8	3.3
02-32		6.0		3.4
02-34	4.6	4.2	2.9	4.3
02-35	4.9	4.3	4.2	6.3
02-37	6.9		5.8	
02-41	3.9	5.4	5.6	5.9
Median	**4.9**	**4.9**	**4.2**	**4.3**

Table 23. Blood potassium levels (mM/L) in Panagen-and placebo-group patients on day 0 and at different control time points. Normal range is 3.7-5.5 mM/L.

	Day 0	Day 21		
		After the 1st CT	After the 2nd CT	After the 3rd CT
**Panagen**				
02-20	4.8	4.6	4.5	4.7
02-21	4.1	4.7	3.8	4.0
02-22		4.8	5.2	
02-24		4.7		
02-25	5.6	5.2	4.6	6.0
02-26	4.3		3.9	
02-27	4.8	5.0		4.8
02-29	4.8	5.5		4.7
02-30	4.4	4.5		4.4
02-31	4.9	4.5	4.6	4.3

02-33	5.0		4.5	4.5
02-36		4.0		
02-43				4.5
02-44		5.4	5.6	
02-45		4.6	4.3	
Median	**4.8**	**4.7**	**4.5**	**4.5**
**Placebo**				
02-23				4.3
02-32		4.7		
02-34	5.4	3.8	4.6	
02-35	5.2	4.5	5.1	
02-41		4.0	3.6	
Median	**5.3**	**4.3**	**4.6**	**4.3**

Table 24. Blood sodium levels (mM/L) in Panagen-and placebo-group patients on day 0 and at different control time points. Normal range is 126-151 mM/L.

	Day 0	Day 21		
		After the 1st CT	After the 2nd CT	After the 3rd CT
**Panagen**				
02-20	145	155	150	143
02-21	142	156	154	144
02-22		149	151	
02-24		147		
02-25	156	152	139	173
02-26	154		142	
02-27	158	150		147
02-29	151	152		146
02-30	152	135		
02-31	152	147	148	
02-33	152		144	144
02-36		141		
02-43				141
02-44		148	147	

02-45		145	143	
Median	**152**	**149**	**147**	**144**
**Placebo**				
02-23				147
02-32		147		
02-34	152	144	145	
02-35	151	146	143	
02-41		144	143	
Median	**152**	**145**	**143**	**147**

Table 25. PHOS (inorganic phosphorus) levels (mM/L) in Panagen-and placebo-group patients on day 0 and at different control time points. Normal PHOS range is 0.82-1.48 mM/L.

	Day 0	Day 21		
		After the 1st CT	After the 2nd CT	After the 3rd CT
**Panagen**				
02-20		1.19		1.20
02-21		1.11		1.25
02-22		1.00		
02-24		1.26		
02-25	1.19	1.39	1.54	1.50
02-26	1.32		0.91	
02-27	0.99	0.95	1.51	1.10
02-29	0.97	1.30	1.26	1.09
02-30	0.95	1.16		1.18
02-31	1.48	1.27	1.55	1.30
02-33	1.01	1.25		
02-36	1.10			1.33
02-40		1.11	1.27	
Median value	**1.06**	**1.19**	**1.39**	**1.23**
**Placebo**				
02-23	1.08			
02-34	1.04		1.20	
02-35	1.19		1.14	

Median value	**1.08**		**1.17**	

Table 26. Percentage of patients in Panagen and placebo groups showing abnormal values of blood biochemical parameters before and during the therapy. Except for creatinine and urea levels, all blood parameters were above the normal range. Creatinine and urea levels in the blood were below the normal range.

Blood parameter	Panagen group		Placebo group	
	Before the therapy	During the study	Before the therapy	During the study
ALT	11	21	0	33
AST	0	16	0	33
Alkaline phosphatase	0	11	17	33
Total bilirubin	5	5	0	0
Creatinine	5	21	33	17
Urea	5	16	0	0
Glucose	26	42	40	80
Cholesterol	53	89	33	83
Potassium	7	13	0	0
Sodium	40	27	20	0
PHOS	0	23	0	0

The following changes in blood biochemistry parameters became evident.

Both ALT and AST values tend to increase relatively to the starting levels in both Panagen- and placebo-group patients (**Tables 15, 16**). This increase is far less pronounced for ALT. The starting ALT levels in Panagen patients are already above the norm, and this parameter reaches 21% during the study. In contrast, ALT levels in the placebo-group patients were normal pre-therapy and rose to 33% during the study. AST levels in both cohorts increased only after the beginning of the therapy, and placebo-group patients were twice as likely to have increased AST levels.

Alkaline phosphatase levels slightly increased in both patient groups, with placebo patients showing somewhat higher frequency of high alkaline phosphatase (**Table 17**). Percentage of patients showing high total bilirubin values stayed unchanged and was only 5% and 0% for Panagen and placebo groups, respectively (**Table 18**). Thus, higher alkaline phosphatase levels in these patients are likely linked to the destruction of bone tissue. Taking into account that this trend is equally pronounced in the placebo group, this adverse reaction is

not related to the Panagen activity. We believe, this pattern is best explained by bone tissue destruction by metastasizing breast cancer or by postpenopausal or senile osteoporosis, which fits the average age of the study participants.

Decrease of creatinine and urea levels below the normal range is only observed in Panagen-group patients, whereas in patients receiving placebo these parameters become normal (creatinine) or stay normal (urea) (**Tables 19, 20**). One of the reasons of this decline could be endogenous protein deficiency caused by pregnancy, impaired hepatic function or glucocorticosteroid therapy; alternatively it may result from insufficient protein intake. Pregnancy and glucocorticosteroids were exclusion criteria; hepatic insufficiency is a contraindication to CT. Therefore, the likeliest reason of lower creatinine and urea levels is lower protein intake, which may be caused by loss of appetite, poor digestion or protein absorbtion.

In both Panagen and placebo groups, we observed a pronounced tendency for blood glucose and cholesterol levels to increase after the beginning of therapy (**Tables 21, 22**).

Increased blood potassium levels were restricted to Panagen group patients, and these incidents were isolated (**Table 23**).

During the study, both groups displayed progressively fewer cases of high blood sodium levels (**Table 24**).

PHOS levels was initially normal in both patient groups and increased in Panagen-group patients reaching the rate of 23% (**Table 25**).

Thus, our analysis shows that Panagen has a pronounced hepatoprotective activity during CT courses.

Analysis of side effects associated with Panagen administration to stage II-IV breast cancer patients during three consecutive CT cycles

Side effects throughout the trial were reported by the patients on days 1, 14 and 21 of each of the CT course throughout the study. The following results were obtained (**Table 27**).

Table 27. Frequencies of symptoms reported by Panagen and placebo-group patients (%).

Symptom	Panagen	Placebo
Herpetic eruption	0	29
Diarrhea	11	43
Abdominal bloating	0	14
Pain under ribs on the righthand side	11	29
Dry skin	53	86
Dry eyes	32	14
Heartburn	32	14

This table shows that placebo group patients tend to report the symptoms associated with weakening immune reaction, such as herpetic eruption on the lips, diarrhea and abdominal bloating.

Herpes simplex virus (HSV) is transmitted through the skin and mucosa and is transported throughout the body through blood and neural routes. HSV-specific antibodies generally appear by the third-fourth week of infection and are persist for the lifetime of the host. Yet the virus persists indefinitely, too. According to the published data, by the age of 15, about 83% of people are HSV-positive, and by the age of 30 years, over 90% of people are infected with HSV. Regional sensory ganglia serve as the major reservoir for the virus, where it establishes a latent infection. Reactivation of latent virus is normally constrained by the host, but when immune reaction weakens reactivation occurs. Thus, the clinical presentation showing HSV reactivation may serve as an indirect measure of the patients' immune status. In this study, the peculiar HSV-related symptoms (mouth sores) were restricted to the placebo group (reported by 29% of patients).

One of the plausible causes of diarrhea and abdominal bloating is intestinal dysbiosis accompanied with epithelium athrophy and compromised local immunity, both of which are caused by cytostatic activity of the CT drugs. In the placebo cohort, diarrhea was reported four

times as often compared to the Panagen-group patients. Abdominal bloating was also reported only in the placebo (14%), but not Panagen cohort.

Taken together, these data support the idea that administration of Panagen in combination with the AC CT regimen results in the decreased drug-mediated immunodeficiency in breast cancer patients.

Pain under the ribs on the righthand side of the body was reported by the placebo-group patients three times more frequently than by Panagen-group patients. This agrees well with the blood biochemistry test results, wherein cytolytic syndrome was similarly 3.3-fold more frequent in the placebo cohort. These data further reinforce the idea regarding possible hepatoprotective activity of Panagen.

Dry skin was reported by placebo-group patients 1.5 times more frequently. This symptom stems from the direct cytostatic effect of CP and doxorubicin on basal cells in the skin, although indirect effects CP and doxorubicin are also possible.

Heartburn and dry eyes constitute negative side effects associated with Panagen therapy, as these symptoms are found two times more frequently in the Panagen cohort.

The rest of the symptoms reported by the patients during the study had isolated incidence, and were not considered in the analysis.

Conclusions

1) When Panagen is combined with the CT to treat breast cancer patients, it prevents development of immune deficiency;
2) The medication has a hepatoprotective activity;
3) Use of Panagen ameliorates the symptoms caused by the cytostatic effects of CT on basal cells in the skin;
4) Possible negative side effects associated with Panagen include heartburn and dry eyes.

Quality of life. Self-perceived quality of life assessment by the patients enrolled in the study (QoL scale).

The following results were obtained when patients' responses to QLQ-C30 questionnaires were analyzed (**Table 28).**

During the study, median values of patients' global health in both Panagen and placebo groups stayed essentially unchanged. Global health was originally lower in the placebo group as compared to the Panagen group, and it tended to decline in placebo patients throughout the study.

Patients' functional state in both groups was very similar, on the order of 80% and remained constant throughout the study.

Symptomatic scale values in both groups increased similarly throughout the CT courses.

Table 28. Data analysis of patients' responses to QLQ-C30 questionnaire. Scale ranged from 0 to 100.

	**Global health**				**Functional scale**				**Symptomatic scale**			
	Day 0	After the 1st CT	After the 2nd CT	After the 3rd CT	Day 0	After the 1st CT	After the 2nd CT	After the 3rd CT	Day 0	After the 1st CT	After the 2nd CT	After the 3rd CT
**Panagen**												
02-20		83.3	83.3	83.3		95.6	95.6	95.6		12.8	12.8	10.3
02-21		50.0	41.7	50.0		77.8	66.7	68.9		30.8	43.6	43.6
02-22		50.0	33.3	66.7		64.4	86.7	91.1		28.2	23.1	17.9
02-25	75.0	58.3			84.4	80.0			17.9	43.6		
02-28	66.7		50.0	66.7	84.4		68.9	77.8	23.1		33.3	10.3
02-29	66.7	66.7			84.4	86.7			5.1	7.7		
02-40	66.7	66.7	66.7	58.3	93.3	95.6	88.9	80.0	7.7	12.8	17.9	28.2
02-42	83.3	83.3			93.3	97.8			5.1	5.1		
02-43		83.3	66.7	75.0		84.4	77.8	86.7		20.5	17.9	15.4
02-45	50.0		41.7	33.3	46.7		35.6	31.1	35.9		56.4	46.2
Median value	**66.7**	**66.7**	**50.0**	**66.7**	**84.4**	**85.6**	**77.8**	**80.0**	**12.8**	**16.7**	**23.1**	**17.9**
**Placebo**												
02-23		33.3	33.3	33.3		80.0	84.4	82.2		15.4	12.8	15.4
02-32		33.3	50.0			73.3	64.4			25.6	30.8	
02-34	33.3			33.3	60.0			77.8	43.6			25.6

02-35	66.7		50.0	50.0	88.9		86.7	84.4	12.8		17.9	15.4
02-37	33.3		33.3		51.1		46.7		51.3		33.3	
02-38	58.3		66.7	58.3	93.3		91.1	93.3	7.7		10.3	12.8
02-41	91.7	75.0	66.7	58.3	100.0	80.0	62.2	73.3	7.7	15.4	38.5	25.6
Median value	**58.3**	**33.3**	**50.0**	**50.0**	**88.9**	**80.0**	**74.4**	**82.2**	**12.8**	**15.4**	**24.4**	**15.4**

QLQ-BR23 questionnaire was specifically developed for breast cancer patients, and our analysis of QLQ-BR23 responses showed that functional activity of Panagen-group patients declined progressively throughout the CT courses. Placebo-group patients had fluctuating functional values, but by the end of the third CT they evened out to reach the initial level (**Table 29).**

Symptomatic scale values display very similar dynamics and increase 2-fold in both groups by the end of the study

Table 29. Data analysis of patients' responses to QLQ- BR23 questionnaire. Scale ranged from 0 to 100.

	Functional scale				Symptomatic scale			
	Day 0	After the 1st CT	After the 2nd CT	After the 3rd CT	Day 0	After the 1st CT	After the 2nd CT	After the 3rd CT
**Panagen**								
02-20		58.3	58.3	58.3		48.9	42.9	40.5
02-21		33.3	33.3	38.9		71.4	35.8	35.8
02-22		22.9	37.5	20.8		48.1	24.0	28.7
02-25	87.5	79.2			35.4	20.8		
02-28	52.1		55.6	47.9	14.3		4.8	10.4
02-29	55.6	66.7			4.2	21.4		
02-40	33.3	41.7	33.3	41.7	14.9	14.1	18.2	14.6
02-42	75.0	68.8			0.0	20.2		
02-43		38.9	29.2	37.5		20.8	5.7	8.9
02-45	0.0		0.0	0.0	68.4		69.9	67.9
Median value	**53.8**	**50.0**	**33.3**	**38.9**	**14.6**	**21.1**	**24.0**	**28.7**
**Placebo**								
02-32		16.7	16.7			38.1	7.9	
02-34	16.7			27.8	29.8			42.3
02-35	50.0		44.4	30.6	17.6		42.9	71.4
02-37	16.7		12.5		33.3		36.8	
02-38	39.6		47.9	45.8	2.4		6.0	1.2
02-41	75.0	33.3	77.8	45.8	13.7	30.6	18.4	24.1
Median value	**39.6**	**25.0**	**44.4**	**38.2**	**17.6**	**34.3**	**18.4**	**33.2**

Conclusions:

1. The most crucial time point after the cytostatic therapy is day 14 after injection of cytostatics. It is at this point that all blood cell counts drop to their minimum values. No significant differences between Panagen and placebo patient groups are observed if the blood parameters are compared across the entire dataset. Nevertheless, protective activity of Panagen is clearly observed. Cell counts (leukocytes, neutrophils and lymphocytes) at two last control time points are significantly lower in the placebo group as compared to the starting, pre-therapy levels. In other words, these cell populations and their progenitors become inhibited as the patients undergo multiple courses of CT. In contrast, these parameters remain essentially unchanged throughout the CT courses in Panagen patients, which points to the Panagen protective activity.
2. The observed protective activity of Panagen is manifested as lower incidence of grade I-IV neutropenias at the control time point on day 14 after the third CT.
3. When Panagen-group patients are subdivided into responders and non-responders, we observe significantly (Wilcoxon-Mann-Whitney test) higher values (for leukocytes, neutrophils, monocytes and lymphocytes) in the responding patients vs placebo-group patients. These data support a stimulatory activity of Panagen towards these cell populations.
4. As it follows from our analysis, Panagen has moderately negative effects on erythroid lineage. Lower RBC counts, Hb levels and Hct levels are observed. Panagen medication helps increase the platelet counts on day 21 after the CT.
5. Panagen has hepatoprotective activity.
6. Panagen alleviates the drug-mediated immunodeficiency in breast cancer patients treated with AC CT.
7. Panagen reduces the secretory function of salivary glands and accessory lacrimal glands.
8. Self-perceived Quality of life scores are very similar in placebo and Panagen cohorts.
