# Supplementary material for: Results of multicenter double-blind placebo-controlled phase II clinical trial of Panagen preparation to evaluate its leukostimulatory activity and formation of the adaptive immune response in patients with stage II-IV breast cancer
Source: BMC Cancer. 2015 Mar 13;15:122. doi: 10.1186/s12885-015-1142-z (PMC4365563; doi:10.1186/s12885-015-1142-z)
Supplement: Additional file 4: — Results of the phase II double-blind multicenter placebo-controlled clinical trial to evaluate the safety and leukostimulatory activity of Panagen in breast cancer patients. The study was performed at the Irkutsk Regional Oncology Dispensary and included 23 patients receiving FAC therapy (18 patients additionally received Panagen, 5 patients received placebo). [file 12885_2015_1142_MOESM4_ESM.pdf]

Additional file 4. Results of the phase II double-blind multicenter placebo-controlled clinical trial to evaluate the safety and leukostimulatory activity of Panagen in breast cancer patients. The study was performed at the Irkutsk Regional Oncology Dispensary and included 23 patients receiving FAC therapy (18 patients additionally received Panagen, 5 patients received placebo).

Twenty-nine patients were enrolled in the study: 22 patients formed Panagen cohort, and 7 were in a placebo group. Four Panagen- and two placebo-group patients discontinued the therapy.

Analysis of leukostimulatory activity of Panagen. Dynamics of various types of WBCs in the peripheral blood of patients enrolled in the study.

Measurements of WBC counts at control time points throughout the three cycles of chemotherapy

Table 1. WBC counts in the peripheral blood samples of patients enrolled in the study, as measured at the starting time point on day 0 and on day 21 after the 1st and 3rd chemotherapies. Significant differences of median values in Panagen-responders subgroup vs placebo patients are shown in red and are marked with an asterisk (*) (p<0.05. Wilcoxon-Mann-Whitney test).

	**Absolute values, $*10^6$ cells/ml**			**Relative values (%), normalized to the baseline**	
	Day 0	After the 1st CT	After the 3rd CT	After the 1st CT	After the 3rd CT
**Panagen**					
01-01	4.5	2.8	5.1	62.2	113.3
01-02	7.0	6.1	7.8	87.1	111.4
01-04	6.6	3.9	3.8	59.1	57.6
01-05	3.2	8.6	7.6	272.2	239.7
01-06	10.2	9.7		95.0	
01-07	4.0	3.9	4.9	96.8	121.6

01-13	9.0	5.9		65.6	
01-14	6.4	5.3		82.4	
01-15	7.9	8.3		105.1	
01-17	7.0	7.9	5.2	113.7	74.8
01-19	5.3	2.7		51.2	
01-20	6.0	9.7	6.4	160.6	105.8
01-21	14.6		7.8		53.6
01-22	7.6	5.1		67.1	
Median value	**6.8**	**5.9**	**5.8**	**87.1**	**108.6***
% Responders				31	63
% Non-responders				69	38
Median for responders				137.1	113.3*
Median for non-responders				67.1	57.6
Combined percentage of responding patients				**50**	
**Placebo**					
01-03	3.2	4.3	2.1	134.4	65.6
01-12	6.1	4.0		65.9	
01-16	12.0		6.0		50.0
01-23	9.2	5.0	3.8	54.3	41.3
Median value	**7.6**	**4.3**	**3.8**	**65.9**	**50.0**

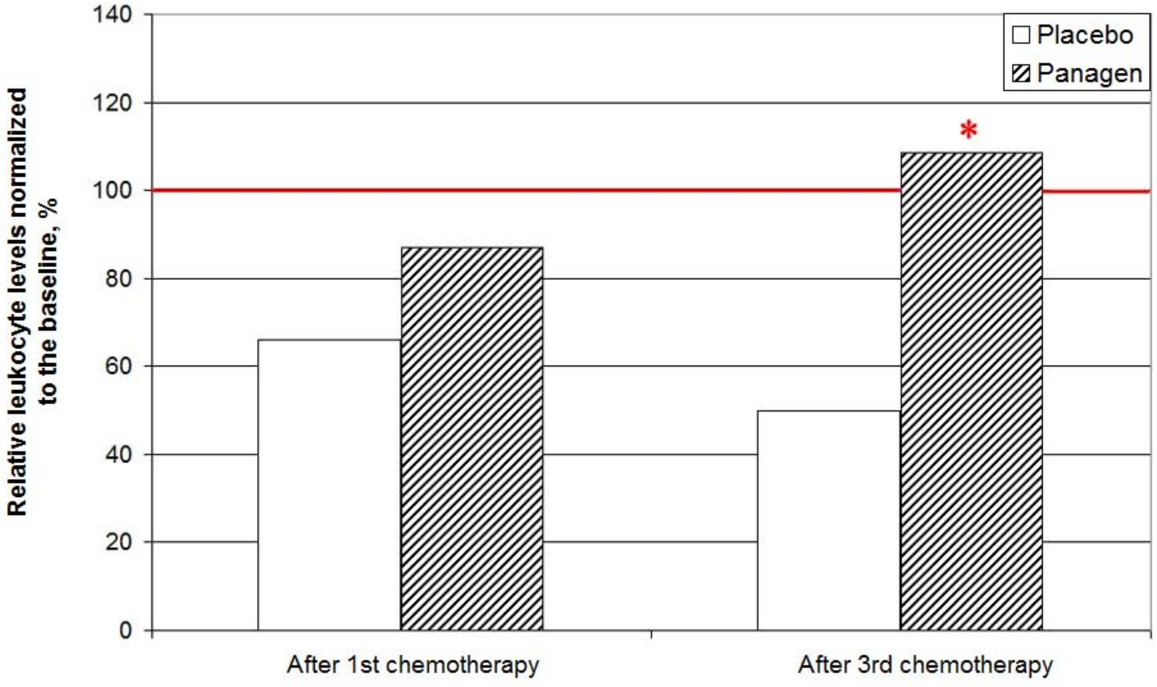

Figure 1. Relative levels of peripheral-blood leukocytes in patients on day 21 after the 1st and 3rd chemotherapies compared to the baseline (set to 100%, red line). Red asterisk denotes significant differences in Panagen-group relatively to the placebo-group ($p<0.05$, Wilcoxon-Mann-Whitney test).

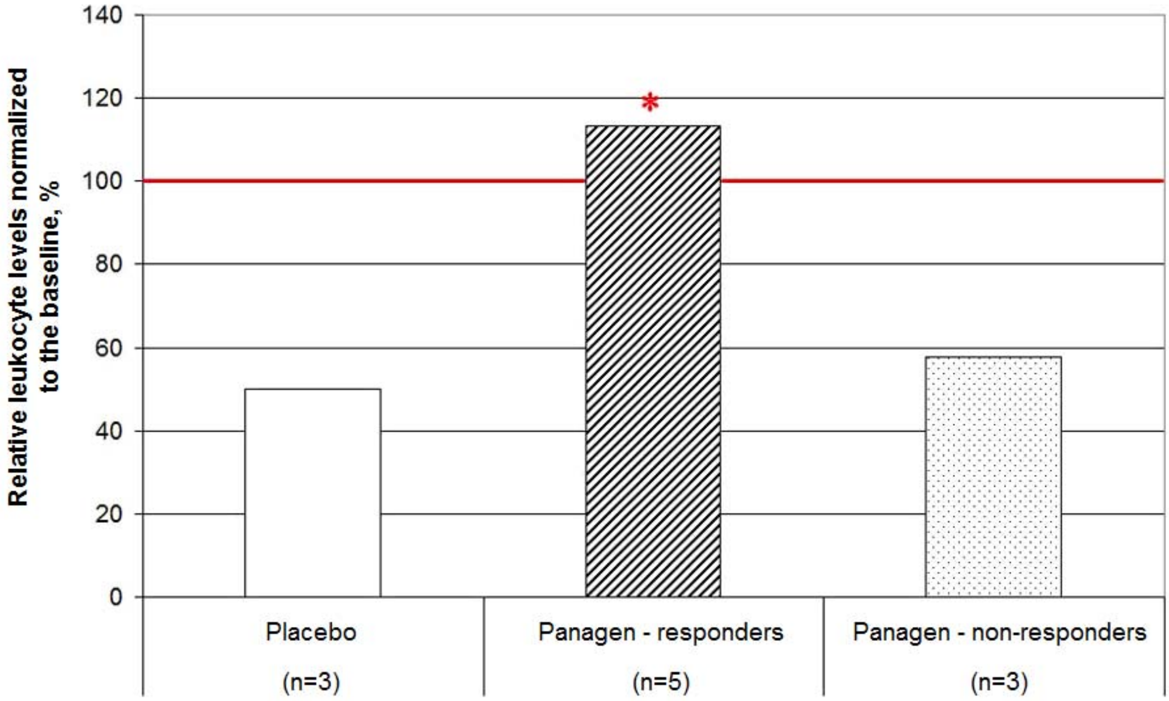

Figure 2. Relative levels of peripheral-blood leukocytes in placebo-group patients, Panagen-responders and Panagen-non-responders on day 21 after the 3rd chemotherapy, normalized to the baseline (set to 100%, red line). Red asterisk denotes significant differences in Panagen-responders relatively to the placebo-group ($p<0.05$, Wilcoxon-Mann-Whitney test).

Measurements of neutrophil counts at control time points across the three cycles of chemotherapies

Table 2. Neutrophil counts in the peripheral blood of patients enrolled in the study, on day 0 and on day 21 after the 1st and 3rd chemotherapies. Median values significantly different from median values of placebo group are shown in blue and marked with an asterisk (*) ($p<0.11$, Wilcoxon-Mann-Whitney test). Median values significantly different from those of the placebo group are shown in red and marked with an asterisk (*) ($p<0.05$, Wilcoxon-Mann-Whitney test).

	**Absolute values, *10^6 cells/ml**			**Relative values (%), normalized to the baseline**	
	Day 0	After the 1st CT	After the 3rd CT	After the 1st CT	After the 3rd CT
**Panagen**					
01-01	2.25	1.06	2.19	47.3	97.5
01-02	5.32	3.72	5.69	69.9	107.0
01-04	4.62	1.91	1.71	41.4	37.0
01-05	2.06	6.95	5.90	337.2	286.2
01-06	5.43	5.45		100.4	
01-07	2.45	2.15	3.16	87.5	129.0
01-13	6.09	3.36		55.1	
01-14	4.14	2.95		71.2	
01-15	5.14	5.42		105.4	
01-17	6.51	4.86	2.54	74.6	39.0
01-19	3.31	0.93		28.2	
01-20	3.50	8.33	3.92	238.1	112.0
01-21	12.06		4.68		38.8
01-22	4.96	2.86		57.7	
Median value	**4.79**	**3.36**	**3.54**	**71.2**	**102.2***
% Responders				31	50
% Non-responders				69	50
Median for responders				171.7	120.5*
Median for non-				57.7	38.9

responders					
Combined percentage of responding patients				43	
**Placebo**					
01-03	1.98	3.05	0.97	153.9	48.7
01-12	4.03	2.00		49.6	
01-16	8.63		3.34		38.7
01-23	7.24	2.92	2.11	40.3	29.1
Median value	**5.64**	**2.92**	**2.11**	**49.6**	**38.7**

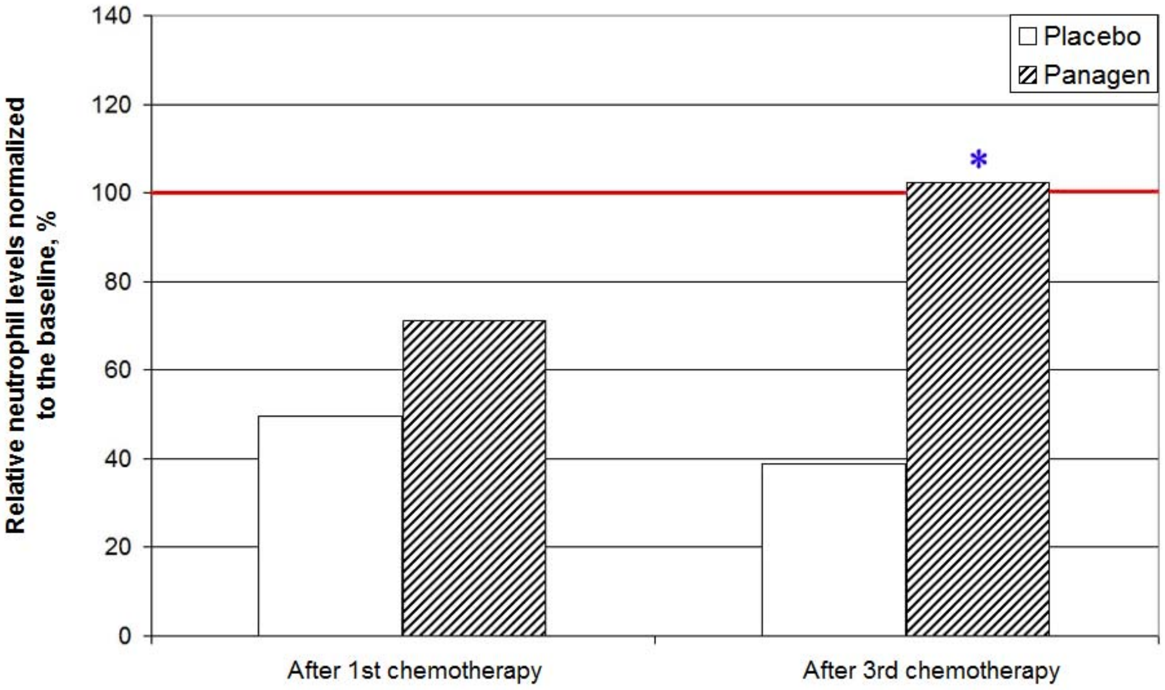

Figure 3. Relative neutrophil levels in the peripheral blood of patients on day 21 after the 1st and 3rd chemotherapies normalized to the baseline (set to 100%, red line). Significant differences of Panagen-group vs placebo-group patients are marked with a blue asterisk (*) ($p<0.11$, Wilcoxon-Mann-Whitney test).

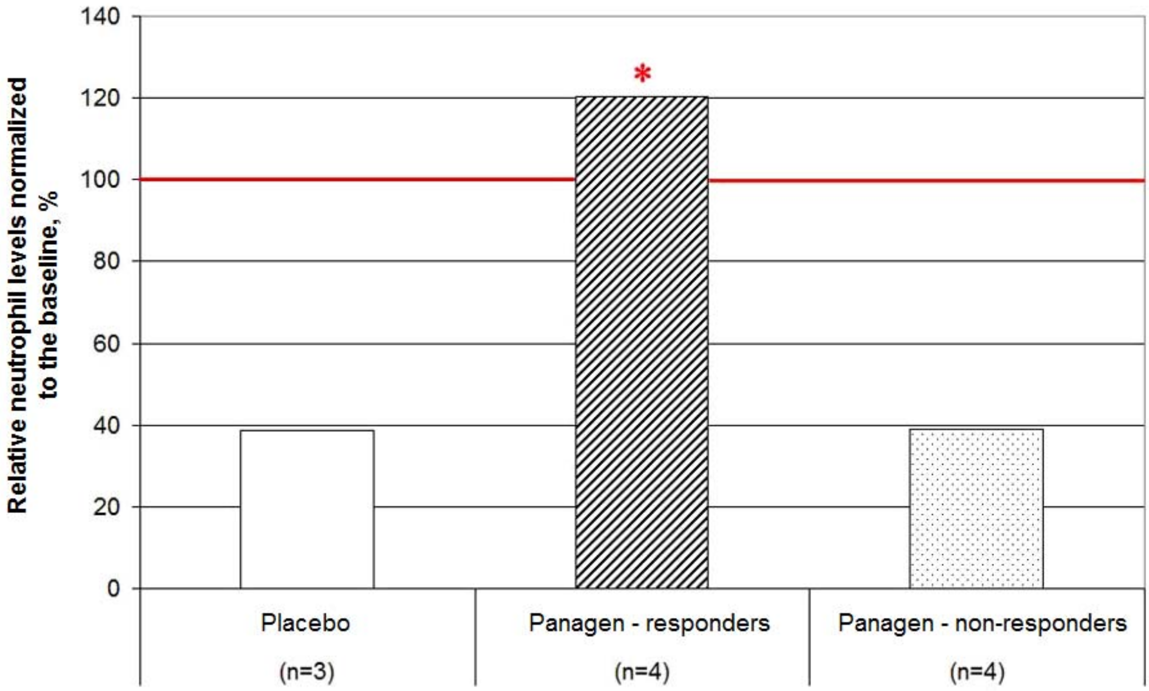

Figure 4. Relative neutrophil levels in peripheral blood of patients from placebo-group, Panagen-responders and Panagen-non-responders, as measured on day 21 after the 3rd chemotherapy and normalized to the baseline (set to 100%, red line). Red asterisk denotes significant differences compared to the placebo group level ($p<0.05$, Wilcoxon-Mann-Whitney test).

Monocyte counts at control time points across the three cycles of chemotherapies

Table 3. Monocyte levels in the peripheral blood of patients enrolled in the study, as measured at the starting time point on day 0 and on day 21 after the 1st and 3rd chemotherapies. Median values in groups that are significantly different from placebo-group values are shown in red and marked with an asterisk (*) ($p<0.05$, Wilcoxon-Mann-Whitney test).

	**Absolute values, *10⁶ cells/ml**			**Relative values (%), normalized to the baseline**	
	Day 0	After the 1st CT	After the 3rd CT	After the 1st CT	After the 3rd CT
**Panagen**					
01-01	0.32	0.62	1.07	195.6	340.0
01-02	0.56	0.79	0.39	141.6	69.6
01-04	0.25	0.39	0.73	155.5	292.4
01-05	0.13	0.76	0.47	598.9	371.6
01-06	0.69	0.70		101.0	

01-07	0.29	0.58	0.50	200.3	173.9
01-13	0.64	0.38		59.0	
01-14	0.40	0.31		76.9	
01-15	0.07	0.85		1190.7	
01-17	0.57	0.72	0.56	126.1	98.5
01-19	0.34	0.49		142.9	
01-20	0.36	0.46	0.81	126.6	225.0
01-21	0.39		1.04		263.8
01-22	0.37	0.52		139.7	
Median value	**0.37**	**0.58***	**0.65**	**141.6***	**244.4**
% Responders				85	75
% Non-responders				15	25
Median for responders				142.9*	278.1*
Median for non-responders				67.9	84.1
Combined percentage of responding patients				**86**	
**Placebo**					
01-03	0.26	0.17	0.40	67.2	155.9
01-12	0.42	0.30		71.4	
01-16	0.51		0.62		122.4
01-23	0.74	0.45	0.58	60.8	78.4
Median value	**0.47**	**0.30**	**0.58**	**67.2**	**122.4**

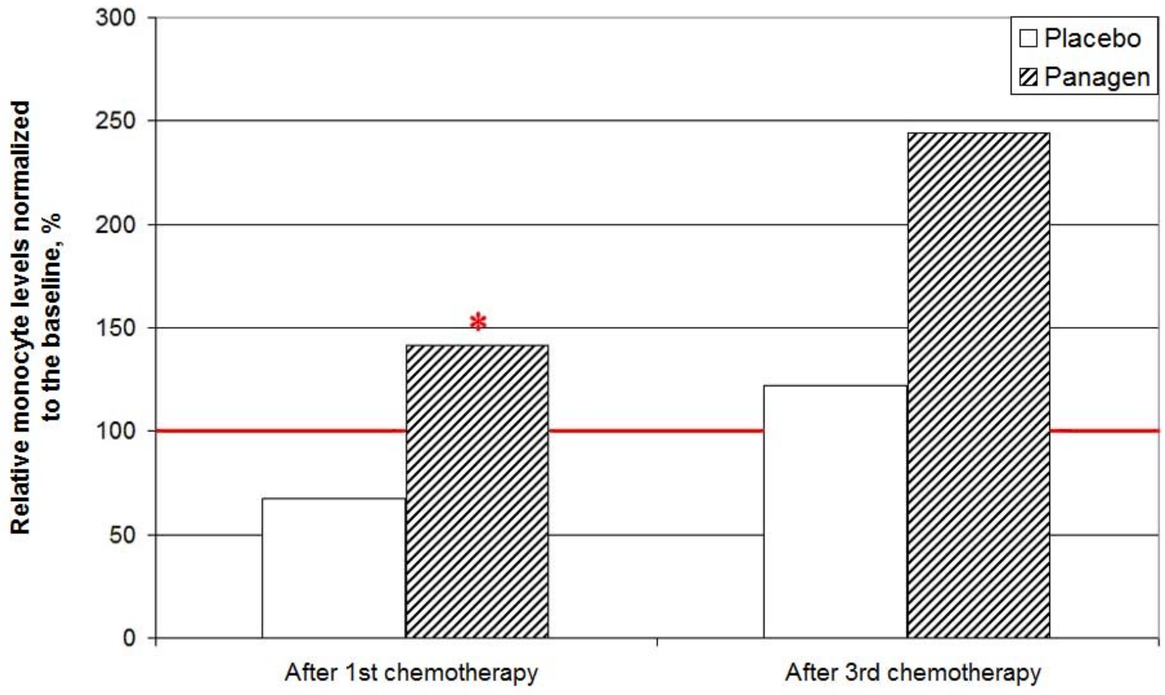

Figure 5. Relative levels of monocytes in the peripheral blood samples of patients on day 21 after the 1st and 3rd chemotherapies normalized to the baseline (set to 100%, red line). Red asterisk denotes significant differences from placebo-group levels ($p<0.05$, Wilcoxon-Mann-Whitney test).

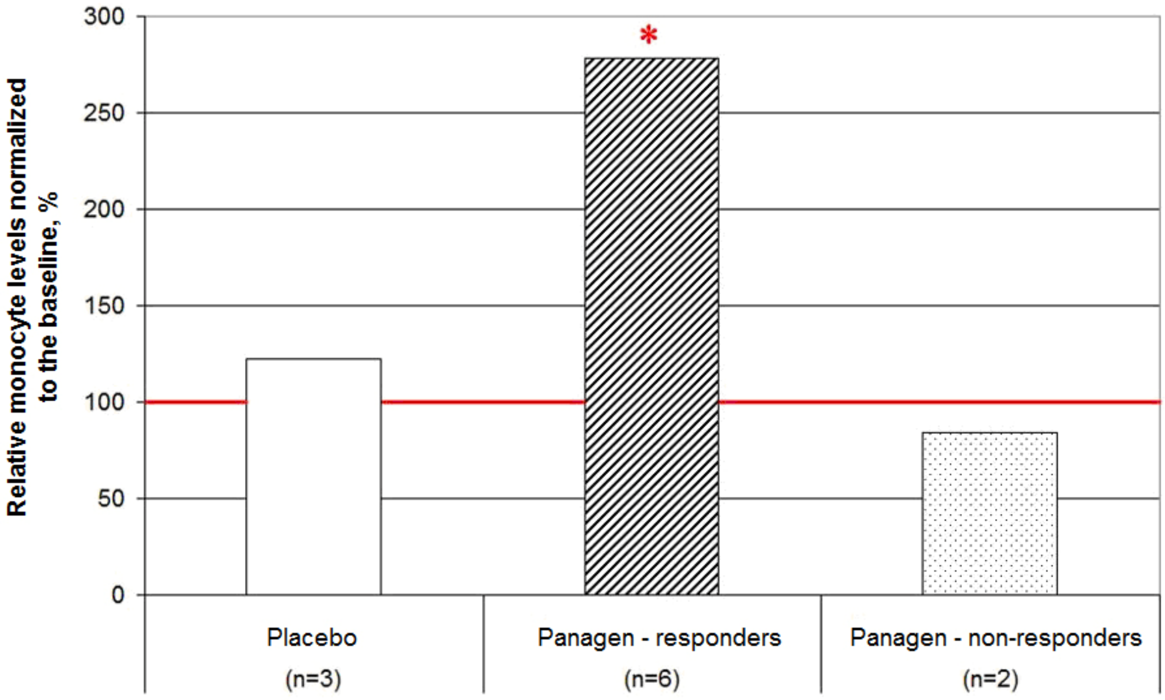

Figure 6. Relative levels of monocytes in the peripheral blood samples of patients from placebo group, responders and non-responders on day 21 after the 3rd chemotherapy normalized to the baseline (set to 100%, red line). Red asterisk denotes significant differences from placebo-group levels ($p<0.05$, Wilcoxon-Mann-Whitney test).

Analysis of lymphocyte counts at control points throughout the three chemotherapy courses

Table 4. Lymphocyte levels in the peripheral blood samples of participating patients at the starting point on day 0 and on day 21 after the 1st and 3rd chemotherapies. Median values in groups that are significantly different from the placebo group are marked with an asterisk (*) (blue, p<0.07, and red, p<0.05, Wilcoxon-Mann-Whitney test).

	**Absolute values, *10^6 cells/ml**			**Relative values (%), normalized to the baseline**	
	Day 0	After the 1st CT	After the 3rd CT	After the 1st CT	After the 3rd CT
**Panagen**					
01-01	1.89	1.06	1.84	56.3	97.1
01-02	0.91	1.34	1.48	147.5	162.9
01-04	1.72	1.52	1.24	88.6	72.0
01-05	0.95	0.91	1.23	95.3	129.5
01-06	3.63	3.24		89.4	
01-07	1.10	1.17	1.23	106.7	112.2
01-13	2.15	2.17		100.7	
01-14	1.75	2.05		116.9	
01-15	2.69	1.83		68.0	
01-17	1.66	2.15	1.86	129.4	112.1
01-19	1.54	1.22		79.1	
01-20	1.81	0.81	1.48	45.0	81.8
01-21	2.15		1.99		92.7
01-22	2.26	1.72		75.9	
Median value	**1.78**	**1.52**	**1.48**	**89.4**	**104.6***
% Responders				38	50
% Non-responders				62	50
Median for responders				116.9	120.8*
Median for non-responders				77.5	87.2
Combined				**43**	

percentage of responding patients					
**Placebo**					
01-03	0.90	1.08	0.69	120.0	77.3
01-12	1.33	1.70		127.8	
01-16	2.69		1.85		68.9
01-23	1.17	1.58	1.02	135.0	87.2
Median value	**1.25**	**1.58**	**1.02**	**127.8**	**77.3**

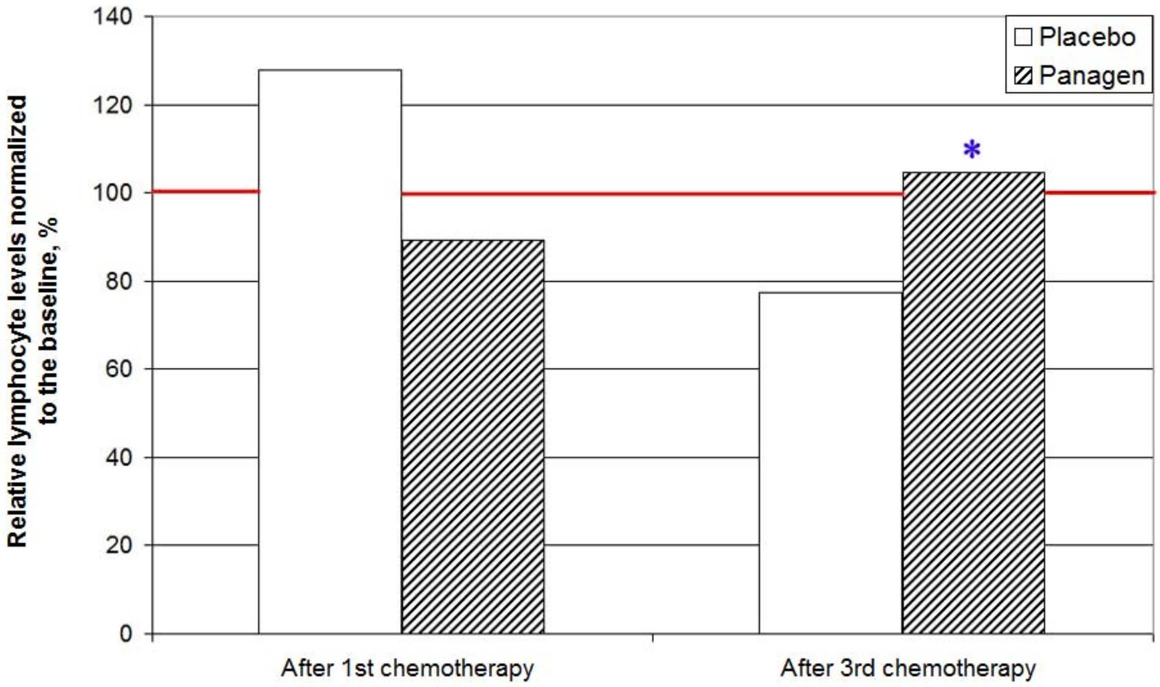

Figure 7. Relative levels of peripheral-blood lymphocytes in patients on day 21 after the 1st and 3rd chemotherapies normalized to the baseline (set to 100%, red line). Blue asterisk marks significant differences from placebo group ($p<0.07$, Wilcoxon-Mann-Whitney test).

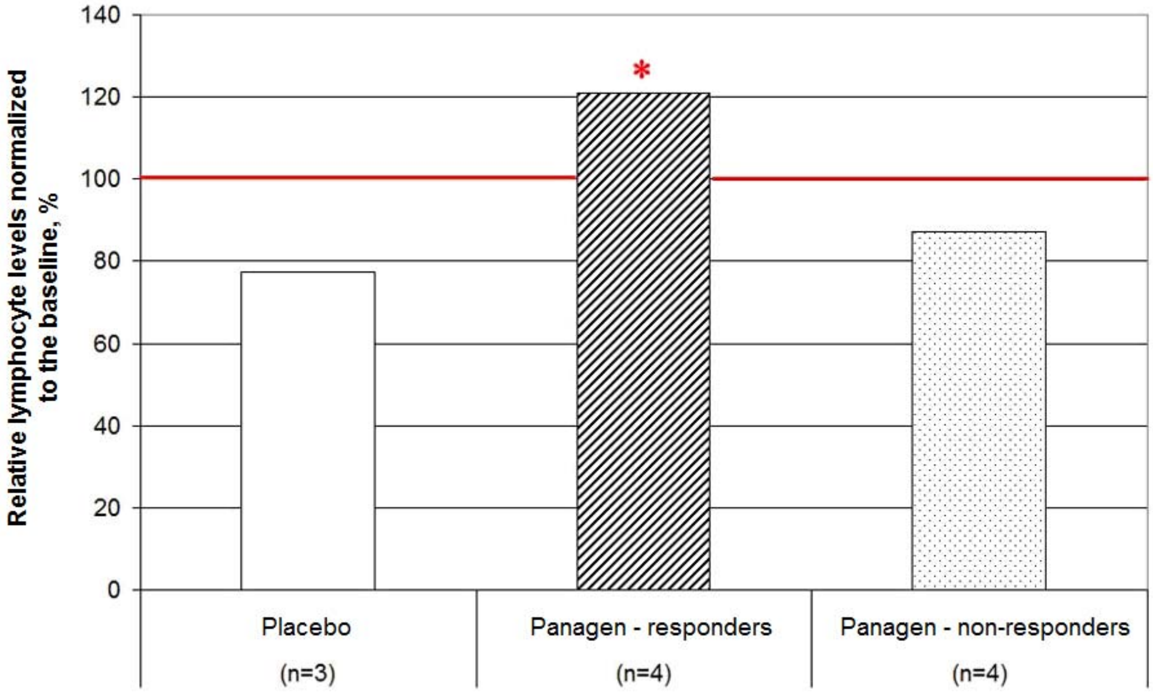

Figure 8. Relative levels of peripheral-blood lymphocytes in patients from placebo group, responders and non-responders subgroups on day 21 after the 3rd chemotherapy normalized to the baseline (set to 100%, red line). Red asterisk denotes significant differences from the placebo group ($p<0.05$, Wilcoxon-Mann-Whitney test).

Impact on erythroid lineage. Erythropoiesis and Panagen activity towards megakaryocyte cell lineage

Overall, RBC counts decline during the therapy in both Panagen and placebo groups (**Table 6**). In Panagen-group patients (14 total), 2 patients (14%) displayed RBC counts below the normal range, and 1 patient (7%) had RBC counts above the normal range. Among placebo-group patients, RBC level below the normal range were found in one out of four patients (25%).

Table 6. RBC counts in blood samples of patients from Panagen and placebo groups at starting time point on day 0 and on day 21 after the 1st and 3rd chemotherapies. Normal range is 3.8 – 5.1 $\times10^{12}$ cells/L.

	Day 0	Day 21 after the 1st CT	Day 21 after the 3rd CT
**Panagen**			
01-01	4.86	4.20	4.59
01-02	4.46	4.68	4.09
01-04	5.05	4.50	4.31

01-05	3.60	2.94	2.79
01-06	5.16	4.63	4.71
01-07	4.68	4.65	4.36
01-13	4.31	4.14	
01-14	5.39	4.93	
01-15	4.88	4.33	
01-17	4.24	3.61	4.04
01-19	4.77	4.32	
01-20	5.06	4.62	4.36
01-21	5.02		5.28
01-22	4.54	4.46	
Median value	**4.82**	**4.46**	**4.36**
**Placebo**			
01-03	4.70	4.34	4.13
01-12	3.91	3.49	
01-16	5.52		4.81
01-23	4.75	4.08	4.15
Median value	**4.73**	**4.08**	**4.15**

Hemoglobin levels in patients' blood samples also showed minor decline in both Panagen- and placebo-group patients (**Table 7**). One Panagen-group patient had hemoglobin level significantly lower than the normal range.

Table 7. Hemoglobin level in the blood of patients from Panagen and placebo groups at the starting time point on day 0, and on day 21 after the 1st and 3rd chemotherapies. Normal Hb range is 120 – 155 g/L.

	Day 0	Day 21 after the 1st CT	Day 21 after the 3rd CT
**Panagen**			
01-01	144	124	146
01-02	138	146	130
01-04	154	143	133
01-05	120	101	92
01-06	160	149	145

01-07	145	143	143
01-13	138	141	
01-14	165	160	
01-15	150	132	
01-17	133	114	124
01-19	151	132	
01-20	146	133	129
01-21	146		140
01-22	129	124	
Median value	**146**	**133**	**133**
**Placebo**			
01-03	132	122	117
01-12	120	113	
01-16	161		134
01-23	147	129	131
Median value	**140**	**122**	**131**

Virtually all patients from both Panagen and placebo groups had platelet counts rising relatively to the starting values (**Table 8**). Platelet counts were above the normal range in 29% and 25% of Panagen- and placebo-group patients, respectively.

Table 8. Platelet counts in the blood of patients from Panagen and placebo groups, as measured at the starting time point on day 0 and on day 21 after the 1st and 3rd chemotherapies. Normal range is 150-400 $\times 10^9$ cells/L.

	Day 0	Day 21 after the 1st CT	Day 21 after the 3rd CT
**Panagen**			
01-01	179	203	190
01-02	298	240	440
01-04	329	348	408
01-05	251	220	321
01-06	275	309	276
01-07	218	281	269
01-13	271	349	

01-14	231	291	
01-15	250	309	
01-17	422	427	373
01-19	204	223	
01-20	328	396	392
01-21	397		440
01-22	295	253	
Median value	**273**	**291**	**373**
**Placebo**			
01-03	172	315	283
01-12	354	422	
01-16	220		212
01-23	169	307	317
Median value	**196**	**315**	**283**

No changes in mean corpuscular hemoglobin concentration (MCHC) were observed in either groups and the values stayed within normal range throughout the study (**Table 9**).

Table 9. MCH in Panagen- and placebo-group patients' blood samples at the starting time point on day 0 and on day 21 after the 1st and 3rd chemotherapies. Normal range is 27 – 34 pg.

	Day 0	Day 21 after the 1st CT	Day 21 after the 3rd CT
**Panagen**			
01-01	29.6		31.8
01-02	30.9	31.2	31.8
01-04	30.5	29.8	30.9
01-05	33.3	34.4	33.0
01-06	31.0	32.2	30.8
01-07	31.0	30.8	32.8
01-13	32.0	34.1	
01-14	30.6	32.5	
01-15	30.7	30.5	
01-17	31.4	31.5	30.6
01-19	31.7	30.6	

01-20	28.9	28.6	29.6
01-21	29.1		26.5
01-22	28.4	27.8	
Median value	**30.8**	**31.0**	**30.9**
**Placebo**			
01-03	28.1	28.1	28.3
01-12	30.7	32.4	
01-16	29.2		27.9
01-23	30.9	31.6	31.6
Median value	**30.0**	**31.6**	**28.3**

Hematocrit (Hct) levels declined slightly throughout the therapy relatively to the starting values, in both Panagen- and placebo-group patients (**Table 10**). Isolated incidents were observed for patients showing Hct values outside the normal range

Table 10. Hct levels in the blood of patients from Panagen or placebo groups, measured at the starting time point on day 0 and on day 21 after the 1st and 3rd chemotherapies. Normal Hct levels range from 35 to 45%.

	Day 0	Day 21 after the 1st CT	Day 21 after the 3rd CT
**Panagen**			
01-01	43.8	41.5	
01-02	42.4	44.7	40.2
01-04	44.3	38.5	40.8
01-05	35.5	30.9	25.8
01-06	47.6	42.6	43.9
01-07	43.5	43.3	39.5
01-13	42.6	37.8	
01-14	51.0	43.6	
01-15	43.4		
01-17	41.0	34.4	38.8
01-19	42.9	40.6	
01-20	44.6	40.5	41.0
01-21	41.0		41.5

01-22	36.1	36.6	
Median value	**43.2**	**40.6**	**40.5**
**Placebo**			
01-03	40.2	37.3	36.2
01-12	36.7	31.0	
01-16	47.8		47.0
01-23	42.2	37.0	38.8
Median value	**41.2**	**37.0**	**38.8**

As it follows from the above analysis, only one parameter, namely platelet counts, displays significant difference between the groups compared. In Panagen-group patients, this parameter tends to increase by the last control time point. In placebo-group patients, platelet counts increased at the first control time point and then declined slightly by the last control time point. Notably, absolute platelet counts are higher in the Panagen group than in the placebo group.

Blood biochemistry at the control points throughout the chemotherapy courses

The following blood biochemistry test parameters were profiled in the patients: glucose, ALT, AST, bilirubin, total protein, creatinine and urea (**Tables 11-18**).

Table 11. Blood glucose levels (mM/L) in Panagen-and placebo-group patients on day 0 and at different control time points. Normal range is 3.3-5.5 mM/L.

	Day 0	Day 7	Day 21		
		After the 1st CT	After the 1st CT	After the 2nd CT	After the 3rd CT
**Panagen**					
01-01	7.4	5.4		5	6.7
01-02		6.4	6.4	5.7	5.9
01-04	5.4	5.4	5.9	4.9	5.1
01-05	6.4	5.5	6.4		
01-06	10.5	5.7	7.9		8.9
01-07	6.6		5.3		6.3
01-09	5.0	4.9			
01-11	5.4	7.3			

01-14	4.7		5.1		
01-15	5.6		6.3		
01-17	5.7	5.4	5.0		5.7
01-18	5.4	5.7			
01-19	5.4	5.3	5.3		
01-20	5.9	5.5	5.0		5.5
01-21	5.3			5.0	5.2
01-22	5.5		5.8	5.6	
Median value	**5.5**	**5.5**	**5.8**	**5.0**	**5.8**
**Placebo**					
01-03	4.9	5.4	4.9	4.9	5.3
01-08		13.9			10.4
01-12	5.1	5.9	4.8		
01-16	5.5				5.6
01-23		5.0	4.5		4.9
Median value	**5.1**	**5.7**	**4.8**	**4.9**	**5.5**

Table 12. ALT levels (U/L) in peripheral blood of patients from Panagen and placebo groups, at starting time point on day 0 and in different control time points. Normal ALT range is 10-44 U/L

	Day 0	Day 7	Day 21		
		After the 1st CT	After the 1st CT	After the 2nd CT	After the 3rd CT
**Panagen**					
01-01	14	32		15	26
01-02		39	19	24	17
01-04	17	10	20	17	10
01-05	17	14	17		
01-06	27	29			36
01-07	36				50
01-14			21		
01-15	18		26		
01-17	13	12	14		27
01-18	16	11			

01-19	12	13	15		
01-20	14	21	35		60
01-21	8			15	21
01-22	10		20	36	
Median value	**15**	**14**	**20**	**17**	**26**
**Placebo**					
01-03	53	72	22	42	34
01-08					24
01-12		10	18		
01-16	10				12
01-23		12	10		20
Median value	**32**	**12**	**18**	**42**	**22**

Table 13. AST levels (U/L) in peripheral blood of patients from Panagen and placebo groups, at starting time point on day 0 and in different control time points. Normal AST range is 10-34 U/L.

	Day 0	Day 7	Day 21		
		After the 1st CT	After the 1st CT	After the 2nd CT	After the 3rd CT
**Panagen**					
01-01	12	25		17	13
01-02		24	20	23	29
01-04	24	21	22	29	20
01-05	24	17	20		
01-06	17	24			31
01-07	23				33
01-14			23		
01-15	18		19		
01-17	15	14	16		21
01-18	20	15			
01-19	17	16	18		
01-20	15	18	20		30
01-21	13			16	19
01-22	17		28	39	

Median value	**17**	**18**	**20**	**23**	**25**
**Placebo**					
01-03	53	53	23	43	35
01-08					35
01-12		14	22		
01-16	22				25
01-23		16	12		23
Median value	**38**	**16**	**22**	**43**	**30**

Table 14. Total bilirubin levels (mkM/L) in peripheral blood of patients from Panagen and placebo groups, at starting time point on day 0 and in different control time points. Normal total bilirubin range is 3.4-17 mkM/L.

	Day 0	Day 7	Day 21		
		After the 1st CT	After the 1st CT	After the 2nd CT	After the 3rd CT
**Panagen**					
01-01	12.6	13.5		10.8	7.6
01-02		10.4	7.1	10.8	10.5
01-04	22.7	35.4	19.7	13.7	20.1
01-05	13.3	9.5	11.6		
01-06		31.0			
01-07	10.9		10.8		
01-09	11.8	11.6			
01-11	11.9	10.5			
01-14	10.7		8.1		
01-15	9.6		11.5		
01-17	9.6	13.0	4.6		10.5
01-18	8.1	15.6			
01-19	14.8	20.1	12.9		
01-20	8.8	13.9	8.6		8.8
01-21	8.1			10.2	9.0
01-22	11.9		9.2	9.5	
Median value	**11.4**	**13.5**	**10.0**	**10.8**	**9.8**
**Placebo**					

01-03	11.1	15.5	6.6	10.0	10.9
01-08		11.1			15.5
01-12	8.6	10.6	8.1		
01-16	10.9				9.4
01-23		13.4	11.7		8.8
Median value	**10.9**	**12.3**	**8.1**	**10.0**	**10.2**

Table 15. Conjugated (direct) bilirubin levels (mkM/L) in the blood of patients from Panagen and placebo groups, measured at the starting time point and at different control points during the therapy. Normal range for direct bilirubin is <25% of total bilirubin level.

	Day 0	Day 7	Day 21		
		After the 1st CT	After the 1st CT	After the 2nd CT	After the 3rd CT
**Panagen**					
01-01	3.4	3.3		0.0	0.0
01-02		0.0	0.0	0.0	0.0
01-04	8.4	10.4	6.5	3.3	7.9
01-05	1.0	0.0	2.0		
01-06		6.5			
01-07	0.0		2.0		
01-09	0.0	2.0			
01-11	0.0	0.0			
01-14	0.0		0.0		
01-15	0.0		0.0		
01-17	0.0	1.5	0.0		
01-18	0.0	4.2			
01-19	3.6	3.2	2.1		
01-20	0.0	3.1	0.0		1.3
01-21	0.0				
01-22	3.6		1.6		
Median value	**0.0**	**3.1**	**0.8**	**0.0**	**0.7**
**Placebo**					
01-03	0.0	2.0	0.0	2.0	0.0
01-08		0.0			0.0

01-12	1.0	0.0	0.0		
01-16	0.0				
01-23		3.0	1.5		0.0
Median value	**0.0**	**1.0**	**0.0**	**2.0**	**0.0**

Table 16. Total protein levels (g/L) in the blood of patients from Panagen and placebo groups, measured at the starting time point and at different control time points throughout the study. Normal range is 64-83 g/L.

	Day 0	Day 7	Day 21		
		After the 1st CT	After the 1st CT	After the 2nd CT	After the 3rd CT
**Panagen**					
01-01	78	77		77	86
01-02		73	75	78	72
01-04	80	71	69	71	75
01-05	70	68	64		
01-06	62	63	73		72
01-07	62		70		69
01-09	63	59			
01-11	77	56			
01-14	70		73		
01-15	74		68		
01-17	69	70	68		75
01-18	75	76			
01-19	74	74	69		
01-20	81	75	72		70
01-21	74			73	76
01-22	74		75	74	
Median value	**74**	**71**	**70**	**74**	**73**
**Placebo**					
01-03	77	76	78	76	75
01-08		73			
01-12	75	73	71		
01-16	77				77

01-23		68	65		79
Median value	**77**	**73**	**71**	**76**	**77**

Table 17. Urea (mM/L) in blood samples of patients from Panagen and placebo groups, measured at the starting time point and at different control time points throughout the study. Normal range is 2.5-8.3 mM/L.

	Day 0	Day 7	Day 21		
		After the 1st CT	After the 1st CT	After the 2nd CT	After the 3rd CT
**Panagen**					
01-01	4.4	4.7		5.7	7.0
01-02		6.6	4.6	4.0	4.6
01-04	6.6	6.4	5.7	5.9	5.1
01-05		7.5	5.9		
01-06	5.8	6.5	4.5		6.2
01-07	4.2				5.8
01-09	3.4	4.5			
01-14	6.0		4.6		
01-15	7.1		4.4		
01-17	4.8	3.7	4.1		5.7
01-18	5.9	2.9			
01-19	5.8	4.2	5.6		
01-20	5.7	3.8	4.0		5.0
01-21	3.6			5.4	4.2
01-22	5.2		6.7	5.0	
Median value	**5.7**	**4.6**	**4.6**	**5.4**	**5.4**
**Placebo**					
01-03	5.8	5.5	5.4	4.6	6.5
01-08		5.7			
01-12	4.4	7.8	3.0		
01-16	7.3				3.6
01-23		3.1	5.1		4.6
Median value	**5.8**	**5.6**	**5.1**	**4.6**	**4.6**

Table 18. Creatinine levels (mkM/L) in the blood samples of patients from Panagen and placebo groups, as measured at the starting time point and at different control time points throughout the study. Normal range is 44-97 mkM/L.

	Day 0	Day 7	Day 21		
		After the 1st CT	After the 1st CT	After the 2nd CT	After the 3rd CT
**Panagen**					
01-01	83	83		90	124
01-02		86	101	85	78
01-04	80	70	81	85	123
01-05	80	83	98		
01-06	78	73	73		85
01-07	80		79		91
01-09	83	73			
01-14	79		104		
01-15	84		80		
01-17	73	95	78		67
01-18	70	91			
01-19	77	99	95		
01-20	79	68	73		76
01-21	69			73	79
01-22	83		89	86	
Median value	**80**	**83**	**81**	**85**	**82**
**Placebo**					
01-03	73	88	87	77	80
01-08		75			71
01-12	78	77	102		
01-16	96				85
01-23		73	110		83
Median value	**78**	**76**	**102**	**77**	**82**

The following trends became apparent when we analyzed blood biochemistry tests of study participants (**Table 19**).

Table 19. Percentage of patients in Panagen and placebo groups showing abnormal values of blood biochemical parameters before and during the therapy. Except for total protein, all blood parameters were above the normal range. Total protein levels in the blood were below the normal range.

Blood parameter	Panagen	Placebo
Glucose	75%	60%
ALT	14%	20%
AST	7%	40%
Total bilirubin	19%	0%
Conjugated bilirubin	27%	0%
Total protein	25%	0%
Urea	0%	0%
Creatinine	40%	40%

Blood glucose levels in most of the Panagen- and placebo-group patients were slightly above the norm. In two patients (one from each groups), the glucose levels were above 10 mM/L, which is likely an individual feature of those patients (**Table 11**).

ALT and AST levels were increased in 1-2 patients from either groups (**Tables 12 and 13**).

In the Panagen group, total bilirubin levels appear higher, which is largely contributed by the indirect bilirubin. This points to either hemolysis of erythrocytes in these patients, or to the attenuation of hemoglobin-converting activity of the liver (**Tables 14 and 15**).

Total protein level in the blood of Panagen-group patients is just below the normal range in 25% cases (**Table 16**).

Urea levels stay normal in all patients throughout the therapy (**Table 17**).

Both placebo- and Panagen-group patients show minor increase in creatinine levels in the blood (**Table 18**).

Conclusions

Analysis of leuko- and erythropoiesis in patients characterized the changes in parameter values and the following effects of Panagen.

1. Our analysis of Panagen's leukostimulatory activity showed that by the end of the third chemotherapy course the leukocyte counts are significantly higher than the starting, pre-therapy levels, which supports the protective and stimulatory effects of Panagen on white blood cell lineage.

2. ANC levels rise upon Panagen administration. By the end of the third chemotherapy course, this effect reaches significance level of $p<0.11$ (Wilcoxon-Mann-Whitney test). When patients are further subdivided into responders and non-responders, the effects become even more significant ($p<0.05$, Wilcoxon-Mann-Whitney test).

3. Panagen medication gradually enhances proliferation of monocytes. At the control time point on day 21 after the first chemotherapy, this parameter is significantly higher in the Panagen group vs placebo group ($p<0.05$, Wilcoxon-Mann-Whitney test). By the end of the third chemotherapy course, monocyte proliferation shows further two-fold increase, and is significantly different between responders and placebo-group patients ($p<0.05$, Wilcoxon-Mann-Whitney test).

4. Committed lymphoid progenitor cells are also positively stimulated by Panagen. By the end of the third chemotherapy, lymphocyte count in Panagen group is significantly higher compared to the placebo ($p<0.07$, Wilcoxon-Mann-Whitney test). When patients are further subdivided into responders and non-responders, the effects become even more pronounced ($p<0.05$, Wilcoxon-Mann-Whitney test).

5. When erythroid and megakaryocyte lineage cells are analyzed, only platelet counts display significant increase upon Panagen administration, which indicates that Panagen is active toward this blood cell lineage.
