# Supplementary material for: Results of multicenter double-blind placebo-controlled phase II clinical trial of Panagen preparation to evaluate its leukostimulatory activity and formation of the adaptive immune response in patients with stage II-IV breast cancer
Source: BMC Cancer. 2015 Mar 13;15:122. doi: 10.1186/s12885-015-1142-z (PMC4365563; doi:10.1186/s12885-015-1142-z)
Supplement: Additional file 5: — Analysis of CD34+/45+ progenitor cell dynamics at control time points throughout the three cycles of chemotherapy. [file 12885_2015_1142_MOESM5_ESM.pdf]

Additional file 5. Analysis of CD34+/45+ progenitor cell dynamics at control time points throughout the three cycles of chemotherapy

Cell counts for CD34+/45+ hematopoietic stem cells (HSCs) were performed on day 7 after the first chemotherapy and on days 21 after each first, second and third chemotherapy cycles according to the protocol of the clinical trial (FAC chemotherapy, Novosibirsk Municipal Hospital No 1).

When analyzing the data obtained, we found that many or all patients in the placebo group responded to the therapy by significantly higher CD34+/45+ HSC counts (as compared to the starting level). To explore this in more detail, the following approaches were used. First, we compared all the parameters obtained from a total dataset. Alternatively, we used Wilcoxon-Mann-Whitney statistic after sub-grouping the Panagen patients into responders and non-responders and matching them to the reference placebo group. Panagen-responders were defined as the patients showing parameter values exceeding the starting values which were set to 100%. We intentionally ignored whether or not this parameter was higher than the starting value observed in the placebo group. Such analysis assumed that even if responding patient had values that were lower than the maximum value observed among the placebo group patients, this nevertheless still argued for the stimulated proliferation of the cell population of interest.

We also performed a general comparison which accommodated the fact that many or even all placebo-group patients similarly responded to the therapy by increased values of the parameter relatively to the pre-therapy level. Notably, sizes of the patient subgroups were different at every control time point.

FAC chemotherapy at Novosibirsk Municipal Hospital No 1

Tables 1 and **2** show absolute and relative levels of CD34+/45+ cells in the peripheral blood of patients.

Table 1. Percentage of peripheral-blood CD34+/45+ cells in patients receiving Panagen or placebo, as measured in different control time points throughout three chemotherapy cycles.

	**Day 0**	Day 7	Day 21		
		After the 1st CT	After the 1st CT	After the 2nd CT	After the 3rd CT
**Panagen**					
02-01	1.90	0.05	0.19	0.03	0.15

02-02	0.03	0.08	0.15	0.20	0.10
02-03	0.02	0.03	0.25	0.70	0.30
02-04	1.20	0.05	0.15	0.05	
02-05	0.14	0.12	0.26	0.14	0.07
02-06	0.09	0.16	0.26	0.22	0.25
02-08	0.38	0.01	0.66	0.50	0.20
02-09	0.02	0.10	0.14	0.09	0.08
02-10	0.02	0.10	0.10	0.08	0.08
02-11	0.05	0.03	0.30	0.22	0.31
02-14	0.03		0.08	0.07	
02-15	0.08	0.02	0.17	0.28	
02-16	0.02		0.24	0.04	0.11
02-18	0.04	0.20			
**Placebo**					
02-07	0.05	0.08	0.19	0.45	0.08
02-12	0.05	0.05	0.20	0.30	0.43
02-13	0.12	0.01	0.20	0.31	
02-17	0.08	0.04	0.55		

Table 2. Relative levels of peripheral-blood CD34+/45+ cells in the patients at different control time points during three chemotherapy cycles, normalized to the starting, pre-therapy level. Median values showing statistically significant difference in Panagen-responders vs placebo-group patients are shown in red and marked with an asterisk (*) ($p<0.05$, Wilcoxon-Mann-Whitney test).

	Day 7	Day 21		
	After the 1st CT	After the 1st CT	After the 2nd CT	After the 3rd CT
**Panagen**				
02-01	2.6	10.0	1.6	7.9
02-02	230.3	454.5	606.1	303.0
02-03	165.0	1250.0	3500.0	1500.0
02-04	4.2	12.5	4.2	
02-05	85.7	185.7	100.0	50.0
02-06	177.8	288.9	244.4	277.8
02-08	3.2	173.7	131.6	52.6

02-09	476.2	666.7	428.6	381.0
02-10	588.2	588.2	470.6	470.6
02-11	68.1	638.3	468.1	659.6
02-14		266.7	233.3	
02-15	25.0	212.5	350.0	
02-16		1000.0	166.7	458.3
02-18	500.0			
Median value	**125.4**	**288.9**	**244.4**	**342.0**
Percentage of responding patients	50	85	85	70
Percentage of non-responding patients	50	15	15	30
Median value for responders	**353.2***	**454.5**	**350.0**	**458.3**
Median value for non-responders	14.6	11.3	2.9	50.0
Combined percentage of responding patients	**86**			
**Placebo**				
02-07	152.0	380.0	900.0	160.0
02-12	100.0	400.0	600.0	860.0
02-13	5.8	166.7	258.3	
02-17	43.8	687.5		
Медиана	**71.9**	**390.0**	**600.0**	**510.0**

Analysis of relative levels of CD34+/45+ cells on day 7 after the first chemotherapy (normalized to their starting levels) uncovered significant and clear difference in HSC counts between placebo and Panagen-responders groups (**Table 2**, **Figure 1**). On day 7 after the first chemotherapy, 50% of Panagen-group patients have higher HSC counts than what is observed in the placebo-group.

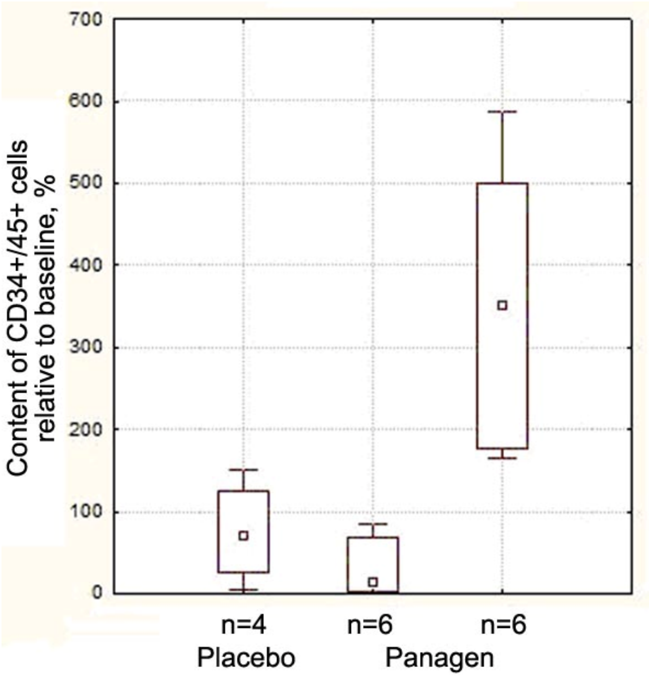

Figure 1. Relative levels of CD34+/45+ cells in peripheral blood samples of participating patients on day 7 after the 1st chemotherapy. Median values in groups, 25-75% quartile range (box) and min/max range are shown. n – the number of patients per group.

As our analysis revealed, by the day 21 of the next cycle of chemotherapy, CD34+/45+ HSC counts exceeded the pre-therapy levels several-fold in both Panagen and placebo cohorts **(Table 2)**. This is explained by the fact that CP-based chemotherapy regimens display sustained mobilizing activity on HSCs and are frequently used in combination with G-CSF in order to maximally activate the proliferation and exit of neutrophil lineage cells into the bloodstream.

We confirmed experimentally the activated proliferation of CD34+/45+ totipotent HSCs by analyzing the cell counts on day 7 following the first chemotherapy. As it turned out, significant differences between Panagen and placebo cohorts are only observed at this control time point. In the rest of the control points the effect of Panagen is masked by the HSC-mobilizing activity of CP.

AC chemotherapy, Novosibirsk Municipal Hospital No 1

Given the data from FAC study, we decided to measure CD34+/45+ cell counts only on day 7 after the first chemotherapy cycle and compare them to the starting pre-therapy levels (**Table 3**).

Table 3. Percentage of peripheral-blood CD34+/45+ cells in patients at the starting point on day 0 and on day 7 after the first chemotherapy. Median values displaying significant difference in Panagen cohort vs placebo cohort are shown in color and marked by an asterisk (*) ($p<0.07$ (blue), and $p<0.05$ (red), Wilcoxon-Mann-Whitney test).

	**Absolute values, % cells in the blood sample**		**Relative values normalized to the starting values, %**
	**Day 0**	**Day 7 after the 1st CT**	
**Panagen**			
02-20	0.19	0.04	21.1
02-21	0.09	0.01	14.4
02-22	0.08	0.05	62.5
02-24	0.03	0.09	264.7
02-25	0.08	0.02	18.8
02-26	0.06	0.02	33.3
02-27	0.04	0.10	250.0
02-28	0.03	0.09	300.0
02-29	0.06	0.01	16.7
02-30	0.05	0.04	78.4
02-31	0.03	0.05	150.0
02-33	0.28	0.20	71.4
02-36	0.05	0.08	168.0
02-39	0.01	0.01	87.5
02-40	0.04		
02-42	0.18	0.02	11.1
02-43	0.03	0.01	16.7
02-44	0.04	0.02	55.0
02-45	0.03	0.01	28.0
Median	**0.05***	**0.03***	**58.8**

Placebo			
02-23	0.02		
02-32	0.01	0.01	128.6
02-34	0.01	0.00	0.0
02-35	0.03	0.00	0.0
02-37	0.10	0.09	90.0
02-41	0.00	0.01	175.0
Median	**0.02**	**0.01**	**90.0**

Three out of 18 patients (16.6%) turned out to have significantly higher hemopoietic progenitor cell counts in their blood at this control time point (**Figure 2**).

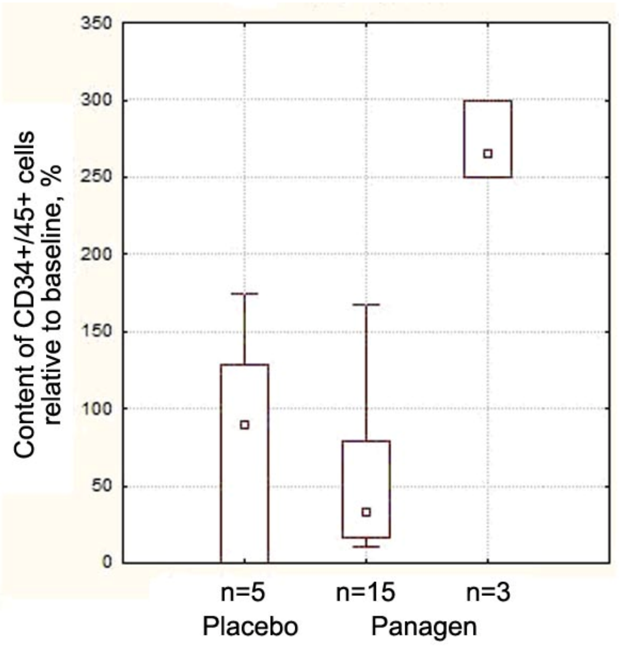

Figure 2. Relative level of peripheral-blood CD34+/45+ cells in participating patients on day 7 after the 1st chemotherapy. Median values in groups, 25-75% quartile range (box) and min/max range are shown. n – the number of patients per group.

FAC chemotherapy, Irkutsk Regional Oncology Dispensary

Table 4. Percentage of peripheral-blood CD34+/45+ cells in patients at the starting point on day 0 and on day 7 after the first chemotherapy. The values in the placebo group a significantly higher than in the Panagen group. Median value is shown in red and marked with an asterisk ($p<0.05$, Wilcoxon-Mann-Whitney test).

	**Absolute values, % cells in the blood sample**		**Relative values normalized to the starting values, %**
	**Day 0**	**Day 7 after**	

		the 1st CT	
**Panagen**			
01-02	0.10	0.16	160.0
01-04	0.10	0.09	90.0
01-05	0.13	0.12	92.3
01-06	0.12	0.03	25.0
01-09	0.15	0.03	20.0
01-10	0.10	0.01	10.0
01-11	0.18	0.11	61.1
01-13	0.07	0.12	171.4
01-15	0.08	0.09	112.5
01-17	0.03	0.14	466.7
01-18	0.18	0.06	33.3
01-19	0.21	0.04	19.0
01-20	0.05	0.02	40.0
01-21	0.15	0.07	46.7
01-22	0.12	0.04	33.3
Median value	**0.12**	**0.07**	**46.7**
Percentage of responding patients			27
Percentage of non-responding patients			73
Median value for responders			165.7
Median value for non-responders			33.3
**Placebo**			
01-03	0.15	0.20	133.3
01-08	0.09	0.12	133.3
01-12	0.05	0.10	200.0
01-16	0.06	0.07	116.7
01-23	0.14	0.22	157.1
Median value	**0.09**	**0.12**	**133.3***

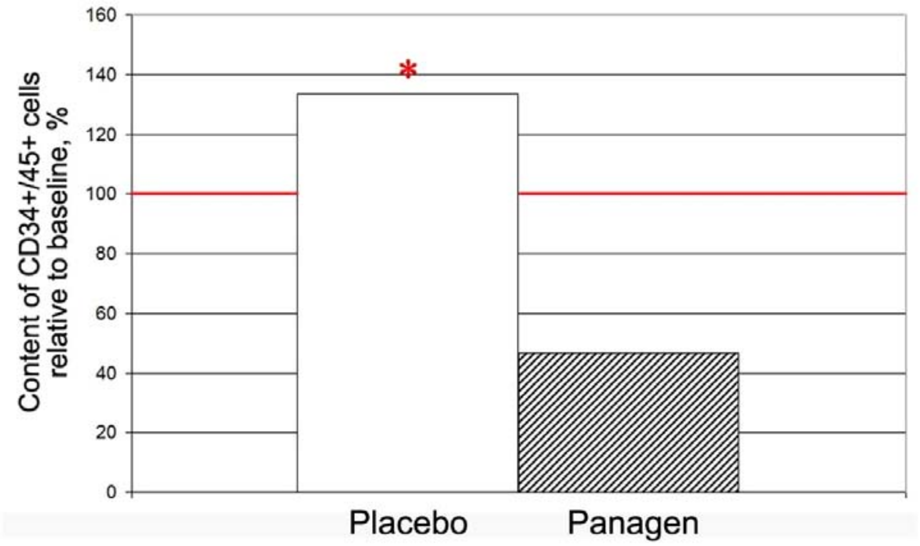

Figure 3. Relative amount of CD34+/45+ lymphocytes in the peripheral blood samples of patients enrolled in the study on day 0 and on day 7 after the 1st chemotherapy. Placebo-group patients display significantly higher values ($p<0.05$, Wilcoxon-Mann-Whitney test) than Panagen-group patients, which is marked by red asterisk (*).

As it follows from the above results (**Table 4, Figure 3**), on day 7 after the beginning of cytostatic therapy, the effects of CP that are clearly seen in the placebo group are abrogated in the Panagen-group patients.

Comparison of the data from three oncology centers

Data from the Irkutsk city Oncology Dispensary indicate that on day 7 after the beginning of cytostatic therapy, the effects of CP otherwise apparent in the placebo group, are cancelled in the Panagen-group patients. Data from the Novosibirsk Municipal Hospital No 1 show that significant response to Panagen therapy (as compared to the placebo treatment) is only observed when comparing relative values for Panagen-responders subgroup.

In light of these observations, we performed comparative analysis of these data **(Figure 4)**. When analyzing the CD34+/45+ cell population, one important parameter is a combination of two distinct categories: % of responding patients and cell counts (%) normalized to the starting values. In this scheme, all patients' values in *both* Panagen and placebo groups were classified as either responders or non-responders. Responders were the patients whose parameter values were higher than they were at the starting time point set to 100%. This analysis allowed accommodating the responding patients in both groups regardless of what exactly their response was to: CP, Panagen or combination thereof.

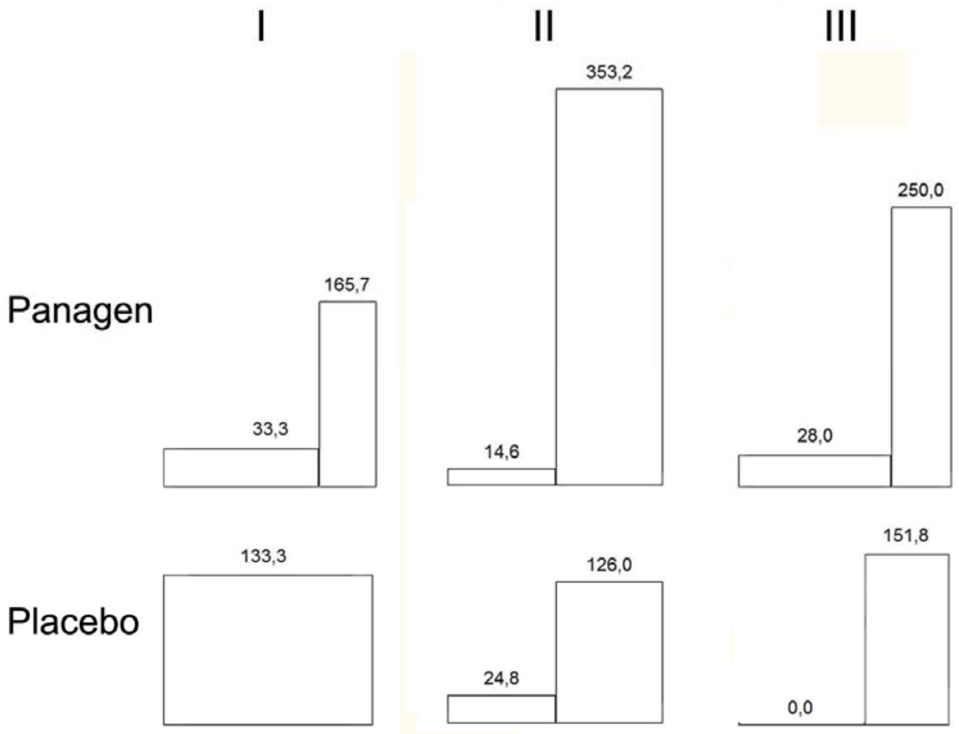

Figure 4. Comparison of parameter values that describe the peripheral-blood CD34+/45+ HSC population in three patient groups recruited to the study. I – FAC regimen in Irkutsk Regional Oncology Dispensary, II – FAC regimen in Novosibirsk Municipal Hospital No 1, and III – AC regimen in Novosibirsk Municipal Hospital No 1. Combined horizontal span of pairs of bars shows the total number of patients in placebo and Panagen groups set to 100%. Height of the bars corresponds to the relative level of HSCs in peripheral blood normalized to the day 0 time point expressed in %. Left and right bars denote median values observed for non-responding and responding patients, respectively. Numbers shown on top of the bars correspond to median values in these groups. Width of the bars matches the number of patients in the group expressed in % (values are given in the text).

As it was mentioned above, monotherapy with CP causes mobilization oh HSCs on days 5-7 after CP administration. Comparison of placebo group across three trial centers shows that the relative levels of peripheral-blood CD34+/45+ HSCs at 120-150% result from the mobilizing activity of CP alone. Hence, further increase in relative HSC levels is indicative of additional involvement of mobilizing properties of Panagen.

We established that all placebo-group patients (100%) from the Irkutsk city Oncology Dispensary responded to the mobilizing activity of CP. The combined positive response to CP and Panagen in the Panagen-group was observed in 27% of patients. This means that Panagen is either cancelling out the mobilizing effect of CP or it is shifting the timing of abortive HSC exit into the bloodstream.

In Novosibirsk-based study, the mobilizing activity of CP was registered in only half of the placebo-group patients (50%), much as in Panagen-group patients (50%). Notably, mobilization of CD34+/45+ HSCs was very pronounced in several patients of Panagen group as compared to placebo.

Given that even one-day difference in timing of blood collection can affect the downstream analyses of HSC mobilization into peripheral blood, the observed data may be partially attributable to slightly different time points when the blood was actually drawn from the patients in Novosibirsk Municipal Hospital No 1 vs Irkutsk Regional Oncology Dispensary.

Alternatively, the discrepancy may stem from the use of FAC-therapy drugs produced by different manufacturers, wherein biological activity of the active substance may necessarily be identical.

As was shown retrospectively, two placebo data points with values of 100% and 5.8% in the Novosibirsk dataset and labeled as obtained on day 7 after the start of chemotherapy were in fact collected on days 5-6. Likewise, in the Panagen group, two samples were drawn not on the day 7 after chemotherapy. In one patient, the HSC count value was 4.2% on day 6 and the other patient was sampled on day 9 and showed the value of 177% relatively to the starting level. As for Irkutsk city Oncology Dispensary, the proper timing of blood collection (day 7) was re-confirmed.

This comparison may support the following features of drug activities.

1) We speculate that the complex of CP, doxorubicin and fluorouracil drugs used throughout the chemotherapies had distinct profiles of biological activity, which may explain the differences in percentage of patients who showed HSC mobilization. The drug cocktail used in Irkutsk center was apparently more biologically active, than that used in Novosibirsk. It is also possible that distinct responses in placebo groups in the two centers were due to the cases of off-schedule blood collection in the Novosibirsk Municipal Hospital No 1. Finally, individual differences between the patients could also contribute to the efficiency of HSC-mobilizing activity of CP.

2) Upon combined synergetic activity of cytostatic drugs and Panagen, mobilization of CD34+/45+ cells occurs somewhat later relatively to the beginning of chemotherapy.

Discussion

In our study, we observed that Panagen has distinct effects on patients as assayed at different control time points. For this reason, to additionally characterize Panagen activity and to unmask its effects on HSCs which would otherwise be hidden by the individual patient variability, we formed 2 patient subgroups, Panagen-responders and –non-responders. This approach was rational by design, because the values from non-responding patients were frequently too low and scattered for the statistical analysis of the entire dataset to return significant differences.

HSCs are known to cycle between the states when they are either permissive for

induction or not. Such HSC states may be related to a certain cell cycle phase (G0), or to the process of leaving the hemopoietic niches and migration down the bloodstream, or it could involve cytokine-induced mobilization or be affected by circadian rhythms (Méndez-Ferrer *et al.*, 2008). It is possible that mobilization process lies in the core of whether cell is susceptible to Panagen effects or not. It is well-established that HSC that left the hemopoietic niches after mobilization by G-CSF or CP are unable to proliferate until they home to the bone marrow and re-establish the ectopic connections with hemopoietic niche components. Analysis of cell cycle distribution in circulating CD34+ cells showed that the pool of G-CSF-mobilized CD34+ cells has fewer S-phase cells and that reduced cycline activity is observed in these cells as compared to the BM-resident CD34+ cells; finally such cells have low metabolic profiles (Lemoli *et al.*, 1997; Uchida *et al.*, 1997; Yamaguchi *et al.*, 1998; Gyger *et al.*, 2000). Using direct methods, it was shown that G-CSF-mobilized CD34+ cells are in G0/G1-phase of the cell cycle, whereas before G-CSF stimulation, many S+G2/M phase CD34+ cells were present in the bone marrow. Importantly, the proliferative capacity of mobilized HSCs was significantly lower than that of BM-resident HSCs. It can be hypothesized that the time spent by the HSCs in circulation and during homing differs among the patients. Clearly then, one would expect to observe significant variation in the time of when HSCs become actively proliferating as they home to the hemopoietic niches. This in turn will be mirrored by the timing of when appropriate lineage cell types will re-appear in the peripheral blood. Given that the control time points are fixed by the protocol design, it is nearly impossible to identify this lag between HSC phases. Thus, the patients whose hemopoietic progenitors have shifted temporal dynamics will likely be opposite in phase to the Panagen-responding patients.

High-dose CP was historically the very first mobilization protocol tested in clinical setting. When hemopoiesis is restored following myelosuppression in the peripheral blood, more than 50-fold increase in CFU-GM counts is observed (CFU-GM is a progenitor of monocytes and neutrophils). Maximum titer of CD34+ HSCs in the peripheral blood was observed on days 5-7 after the cytostatic injection. This effect was the major hurdle in our attempt to estimate and compare the effects of Panagen on hemopoiesis in our patients. Upon administration of CP, the entire BM-resident hemopoietic system faces tremendous stress, and it is very difficult to demonstrate the hemopoietic activity of an additional medication, when this background is already present, and so Panagen effects will be masked by the CP activity. In this regard, the data obtained suggest that Panagen modulates the mobilizing pulse of CP by altering the time of CD34+/45+ HSC migration into the blood stream.

When we analyzed the hemopoietic activity of Panagen towards totipotent CD34+/45+ HSCs in Irkutsk center patients, we observed that Panagen canceled out the mobilizing activity

of CP. Following the assumption of general stimulation of hemopoiesis, this may indicate that Panagen counteracts non-physiological detachment of HSCs from endosteum and migration of HSCs from the stromal niches into the bloodstream. Possibly it is this fact that lies in the basis of activated proliferation observed in the study. It is also possible that HSCs sense these stromal niches as a more physiologic environment, and so cell division is not arrested, which would otherwise occur had such HSC migrated to the bloodstream following CP injection.

We conclude that there are three major opportunities for dsDNA to act upon CD34+/45+ HSCs and to increase their proliferative potential following the cytostatic therapy with CP, doxorubicin and fluorouracil. These include:

1. activation of HSC proliferation by double-stranded ends of DNA molecules;
2. stimulation of cytokine production by immune cells that induce HSC proliferation;
3. maintenance of HSCs in their stromal niches.
