# Supplementary material for: Results of multicenter double-blind placebo-controlled phase II clinical trial of Panagen preparation to evaluate its leukostimulatory activity and formation of the adaptive immune response in patients with stage II-IV breast cancer
Source: BMC Cancer. 2015 Mar 13;15:122. doi: 10.1186/s12885-015-1142-z (PMC4365563; doi:10.1186/s12885-015-1142-z)
Supplement: Additional file 6: — Summary of experiments characterizing development of adaptive immune response. [file 12885_2015_1142_MOESM6_ESM.pdf]

Additional file 6. Summary of experiments characterizing development of adaptive immune response

Tests performed to evaluate the development of adaptive immunity in patients are summarized in **Table 1**.

Given that antigen-presenting cells such as macrophages and DCs differentiate from monocytes, stimulation of monocyte lineage by Panagen should be considered as a positive outcome of the therapy. To explore this in more detail and to delineate the possible pathways of the mounting immune response, we performed experiments to estimate the abundance and ratios of myeloid (CD11+CD123–) and plasmacytoid (CD11–CD123+) DCs in peripheral blood of recruited patients. Analysis of dynamics of cytotoxic CD8+ perforin+ T cells in peripheral blood will serve as a direct measure of developing adaptive immune response in patients participating in the Panagen clinical trial.

To analyze the results, Panagen-group patients were subdivided into "responders" and "non-responders". A patient was considered as a responder if the parameter value was greater than the starting (baseline) level taken as 100%. Data for responding and non-responding subgroups were compared to placebo group. Importantly, the increase of the parameter value above the starting value in placebo group was not taken into account. Under this form of estimate, even if the values observed in responders were lower than the maximal level observed among placebo patients, this nevertheless argued for the stimulation of the cell population analyzed. The results of this approach are summarized in the tables.

Alternatively, we performed a distinct type of analysis, whereby placebo group was taken as a reference sampling, and its min/max values were taken as a norm. In this approach, we calculated median values, quartile ranges (25-75%) and minimum-maximum ranges and compared them to the values obtained in the placebo group. As a result, the values were grouped as normal, above the norm, and sometimes below the norm. The number of patients in each group was also compared. The results of this approach are illustrated in the figures.

We also took into account the fact that some patients may not respond to Panagen at a given control point, whereas others may respond. Importantly, in a different control point, non-responding patients may become responding.

Our analysis of the activation of adaptive immunity involved FAC-regimen patients treated at the Novosibirsk Municipal Hospital No 1. Quantification of cytotoxic CD8+ perforin+ T cells in peripheral blood was also done for AC patients.

Table 1. The list of tests performed to analyze the development of adaptive immune response in patients receiving a combination of cytostatic drugs and Panagen.

Tests	Method/Equipmento/reagents	Test output
1. Relative and absolute quantification of myeloid (CD11c+CD123–) and plasmacytoid (CD11c–CD123+) DCs in peripheral blood of patients	Flow cytometry/ FACSCalibur/ monoclonal antibodies, lysis buffer and fixing solution	Changes in DC numbers as well as the dynamics of DC subpopulations (but not their maturity) throughout the therapy
2. Relative and absolute quantification of T-regs (CD4+CD25+FoxP3+ or CD4+CD25+CD127–), cytotoxic (CD8+ perforin+) T-cells in peripheral blood of patients.	Flow cytometry/ FACSCalibur/ monoclonal antibodies, lysis buffer and fixing solution	Dynamics of suppressive T-regs and cytotoxic T-cells throughout the therapy
3. Analysis of non-specific antitumor cytotoxicity of patients PBMCs. Human breast adenocarcinoma cell line MCF-7 was used as a target	MTT assay with MCF-7 cells	Analysis of protective and stimulatory activity of Panagen exerted on the cells of innate antitumor immunity

Analysis of relative abundance of myeloid (CD11c+CD123–) and plasmacytoid (CD11c–CD123+) DCs in the peripheral blood of study participants

We established that the counts of myeloid and plasmacytoid DCs was different in patient groups as measured in the control time points of the protocol.

Quantification of **plasmacytoid DCs (CD11–CD123+)** in peripheral blood samples (**Table 2**) shows that out of 12 patients in the group, a total of 9 patients (i.e. 75%) responded to Panagen after 1 and 3 courses of chemotherapy. By the third control point, on day 21 after the 3rd round of chemotherapy, 45% of patients displayed increase in plasmacytoid DC counts in peripheral blood ($p<0.11$, Wilcoxon-Mann-Whitney test) (**Table 2**). We speculate that responses to Panagen therapy across different control points are highly individual.

Table 2. Percent of CD11–CD123+ plasmacytoid DCs in peripheral blood samples of patients in the trial. Median value in the Panagen-responders group (colored blue and marked with an asterisk (*)), is significantly higher than that in Placebo group ($p<0.11$, Wilcoxon-Mann-Whitney test).

	**% CD11–CD123+ cells in the blood sample**			**% increase, relatively to the starting value on day 0**	
	Day 0	Day 21 following the 1st CT	Day 21 following the 3rd CT	Day 21 following the 1st CT	Day 21 following the 3rd CT
**Panagen**					
02-01	2.00	5.26	0.84	263.0	42.0
02-02	0.13	0.12	0.80	92.3	615.4
02-03	0.88	0.54	0.52	61.4	59.1
02-04	0.42	0.57		135.7	
02-05	0.34	0.12	0.75	35.3	220.6
02-06	0.96	0.61	0.49	63.5	51.0
02-08	0.34	0.30	0.64	88.2	188.2
02-09	0.68	0.36	1.40	52.9	205.9
02-10	0.16	0.87	0.06	543.8	37.5
02-11	0.29	0.90	0.10	310.3	34.5
02-14	0.39	0.60	0.48	153.8	123.1
02-15	0.78	0.21	0.18	26.9	23.1
Median value				**90.3**	**59.1**

Responders, %				42	45
Non-Responders, %				58	55
Median value (Responders)				263.0	205.9*
Median value (Non-responders)				61.4	39.8
Total responders, %				**75**	
**Placebo**					
02-07	0.30	0.24	0.53	80.0	176.7
02-12	0.22	0.97	0.26	440.9	118.2
02-13	0.23	0.41	0.42	178.3	182.6
Median				**178.3**	**176.7**

When placebo group is considered as a reference, the following trends are observed **(Figure 1).** Panagen-group patients clearly split into two subgroups, i.e. those with values above the statistical reference (n=4), and those below or similar to the statistical reference group (n=7).

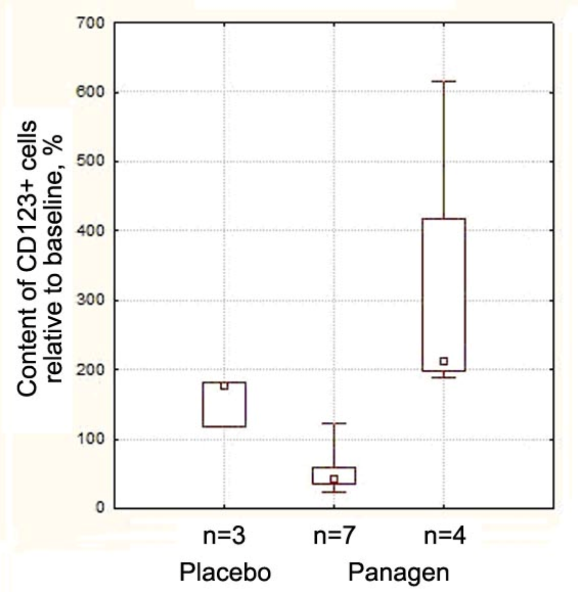

Figure 1. Relative percentage of CD11–CD123+ plasmacytoid DCs in peripheral blood samples of patients on day 21 after the 3rd chemotherapy. Placebo group served as a statistical reference. Median values in groups, 25-75% quartile range (box) and min/max range are shown. n – the number of patients per group.

Analysis of population dynamics of **myeloid DCs (CD11+CD123–)** in peripheral blood of patients participating in the trial is shown in **Table 3** and is summarized in **Fig. 2.**

Table 3. Percent of CD11+CD123– myeloid DCs in peripheral blood samples of patients participating in the trial.

	**% CD11+CD123– cells in the blood sample**			**% increase, relatively to the starting value on day 0**	
	Day 0	Day 21 following the 1st CT	Day 21 following the 3rd CT	Day 21 following the 1st CT	Day 21 following the 3rd CT
**Panagen**					
02-01	2.00	3.00	0.84	150.0	42.0
02-02	0.42	0.39	1.40	92.9	333.3
02-03	1.90	0.78	0.78	41.1	41.1
02-04	0.90	1.43		158.9	
02-05	0.59	0.37	1.05	62.7	178.0
02-06	2.40	0.80	0.81	33.3	33.8
02-08	0.42	0.86	0.85	204.8	202.4
02-09	0.68	0.87	0.99	127.9	145.6
02-10	0.18	1.20	0.15	666.7	83.3
02-11	1.71	1.13	0.24	66.1	14.0
02-14	0.88	0.86	0.75	97.7	85.2
02-15	0.93	0.51	0.18	54.8	19.4
Median value				**95.3**	**83.3**
Responders, %				42	36
Non-Responders, %				58	64
Median value (Responders)				158.9	190.2
Median value (Non-responders)				62.7	41.1
Total responders, %				**58**	
**Placebo**					
02-07	0.80	0.35	1.60	43.8	200.0
02-12	0.97	1.46	0.30	150.5	30.9
02-13	0.32	0.32	1.02	100.0	318.8
Median				**100.0**	**200.0**

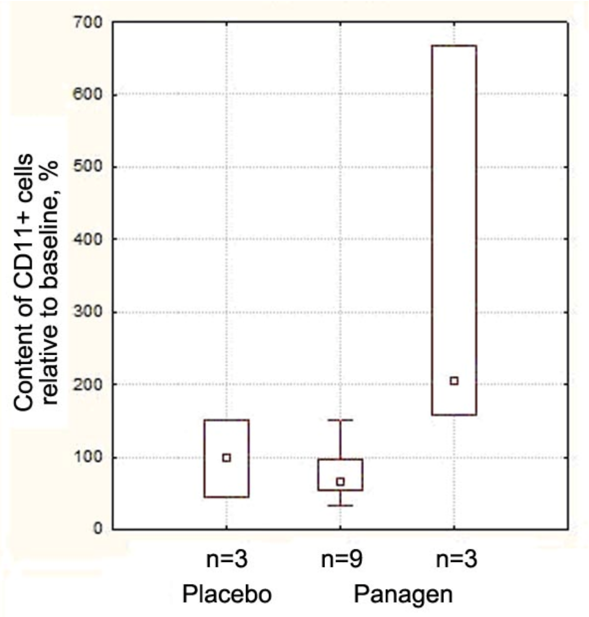

Figure 2. Relative percentage of CD11+CD123– myeloid DCs in peripheral blood samples of patients on day 21 after the 1st chemotherapy. Placebo group served as a statistical reference. Median values in groups, 25-75% quartile range (box) and min/max range are shown. n – the number of patients per group.

Notably, the values observed for both plasmacytoid and myeloid DCs from placebo-group patients are higher than the day 0 values. This is consistent with the published data on stimulatory activity of CP on development of DCs in the bone marrow.

CP is known to increase CD11+ DC counts in peripheral blood on days 9-16 post-injection (Salem *et al*., 2009). DCs thus formed induce proliferation of cytotoxic CD8+ T-cells. With this in mind, assaying the activity of Panagen by quantifying the dynamics of DC subpopulations or cytotoxic T cells is not trivial, as these effects will be necessarily confounded by the action of CP. Nevertheless, should there be significant differences, the observed changes are attributable to the Panagen effect. Notably, measurements of DC counts were performed on day 21 following the chemotherapies, which is likely not the time when these cells peak in the peripheral blood.

Quantitative analysis of perforin-positive cytotoxic T cells in the peripheral blood of patients participating in the trial

We measured cell counts of perforin-positive cytotoxic T cells in peripheral blood of FAC-treated patients (**Table 4**). Using Wilcoxon-Mann-Whitney statistic, we showed that after the first round of chemotherapy Panagen-treated patients display significantly more CD8+ perforin+ T cells in their peripheral blood than do placebo-group patients.

If the values observed in the Panagen group are compared to the maximum value observed in the placebo group, 12 Panagen patients (58%) responded to the treatment, as measured on day 21 after the first course of chemotherapy. On 21 following the 3rd CT, 5 patients out of 9 (56%) displayed higher cytotocxic T-cell counts in their peripheral blood than what is maximally achieved by placebo patients **(Figure 3).** When data from both control points (21 days after 1st and 3rd rounds of CT) are combined, 8 out 12 Panagen-group patients (67%) positively responded to the treatment.

Table 4. Percentage of CD8+ perforin+ cytotoxic T cells in peripheral blood samples of patients treated under the FAC regimen. Median values for the subgroup of Panagen responders are significantly higher than those observed in the placebo group, and are shown in red and marked by an asterisk (*) ($p<0.05$, Wilcoxon-Mann-Whitney test).

	**Absolute values, % cells in the blood sample**			**Relative values, % normalized to the starting value**	
	Day 0	Day 21 after the 1st CT	Day 21 after the 3rd CT	Day 21 after the 1st CT	Day 21 after the 3rd CT
**Panagen group**					
02-01	2	4	11	200.0	525.0
02-02	3	9	13	300.0	420.0
02-03	9	9	2	100.0	23.3
02-04	4	6		150.0	
02-05	16	6	17	39.4	106.3
02-06	14	17	7	121.4	50.0
02-08	17	11	20	61.8	117.6
02-09	10	15	16	147.0	160.0
02-10	8	15	15	178.6	178.6
02-11	17	21	10	123.5	58.8
02-14	15	5		33.3	
02-15	6	8		133.3	
Median value				**128.4**	**117.6**
Responders, %				75	67
Non-Responders, %				25	33
Median value (Responders)				147.0*	169.3

Median value (Non-responders)				39.4	50.0
Total responders, %				**92**	
**Placebo group**					
02-07	18	8	19	46.7	105.6
02-12	9	11	10	122.2	111.1
02-13	14	9		64.3	
Median value				**64.3**	**108.3**

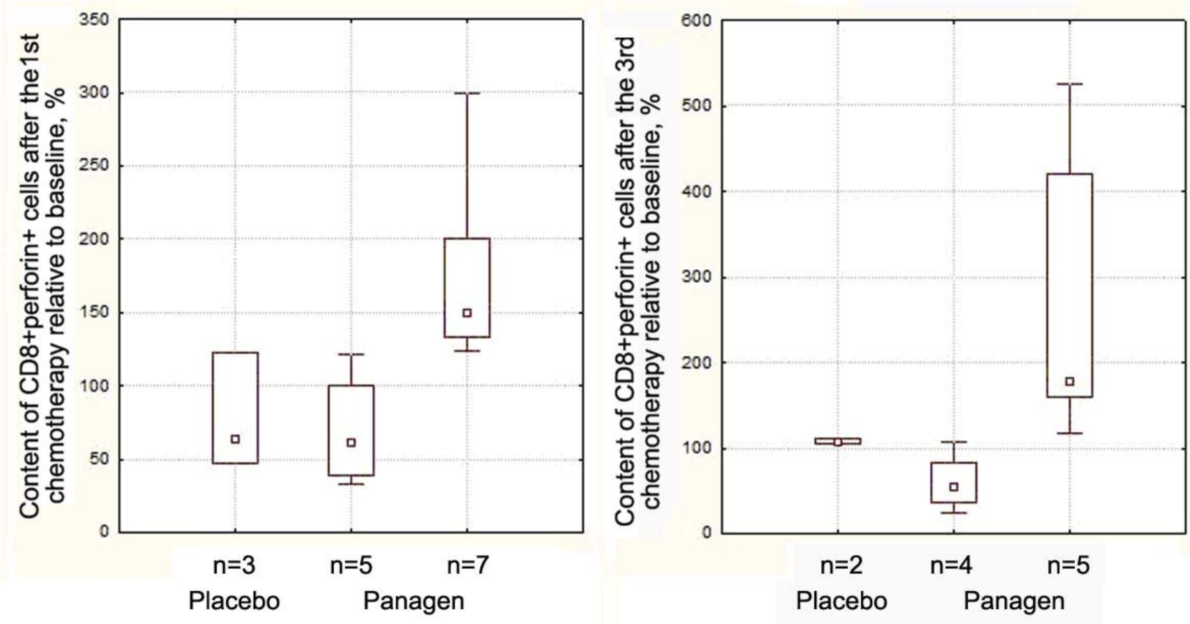

Figure 3. Relative abundance (%) of CD8+perforin+ cytotoxic T cells in the peripheral blood of FAC patients on day 21 after the 1st and the 3rd course of chemotherapy. Data were compared to the placebo group patients, whose minimum and maximum values were taken as a statistical norm. Median values, 25-75% quartile range (box) and min/max range (whiskers) are shown for each group; n – the number of patients per group.

Our data support the idea that breast cancer patients who received Panagen throughout the three courses of chemotherapy had features consistent with the developing adaptive immune response. Lower cytotoxic T cell counts observed in the control points are apparently due to migration of lymphocytes to the tumor focus from the bloodstream.

We also obtained a quantitative estimate of perforin-positive T-cell content in the peripheral blood samples of patients receiving AC regimen (**Table 5, Figure 4**).

Table 5. Percent of CD8+perforin+ cytotoxic T cells in the peripheral blood samples of AC patients.

	**Absolute values,**	**Relative values, %**

	% cells in the blood sample			normalized to the starting value	
	Day 0	Day 21 after the 1st CT	Day 21 after the 3rd CT	Day 21 after the 1st CT	Day 21 after the 3rd CT
**Panagen group**					
02-20	12	10	12	83.3	100.0
02-21	16	14	14	87.5	87.5
02-22	18	16	11	88.9	61.1
02-24	19	13		68.4	
02-25	18	20	21	111.1	116.7
02-26	10	14	13	140.0	130.0
02-27	13	9	20	69.2	153.8
02-28	14	14	12	100.0	85.7
02-29	12	13	28	108.3	233.3
02-30	11	9	6	81.8	54.5
02-31	8	22	4	275.0	48.8
02-33	12	11	10	91.7	83.3
02-36	12	7	17	60.8	139.2
02-39	17	10	16	58.8	94.1
02-40	5	20	14	425.5	297.9
02-42	15	16	11	106.7	73.3
02-43	4	17	2	425.0	50.0
02-44	5	5	6	100.0	120.0
02-45	24	16	23	66.7	95.8
Median value	**12**	**14**	**13**	**91.7**	**95.0**
Responders, %				47	44
Non-Responders, %				53	56
Median value (Responders)				111.1	134.6
Median value (Non-responders)				75.5	78.3
Total responders, %				**63**	
**Placebo group**					
02-23	8	13	13	162.5	162.5
02-32	6	8	11	133.3	183.3

02-34	18	23		127.8	
02-35	25	25		100.0	
02-37	13	7	8	50.0	61.5
02-41	7	13	19	185.7	271.4
Median value	**11**	**13**	**12**	**130.6**	**172.9**

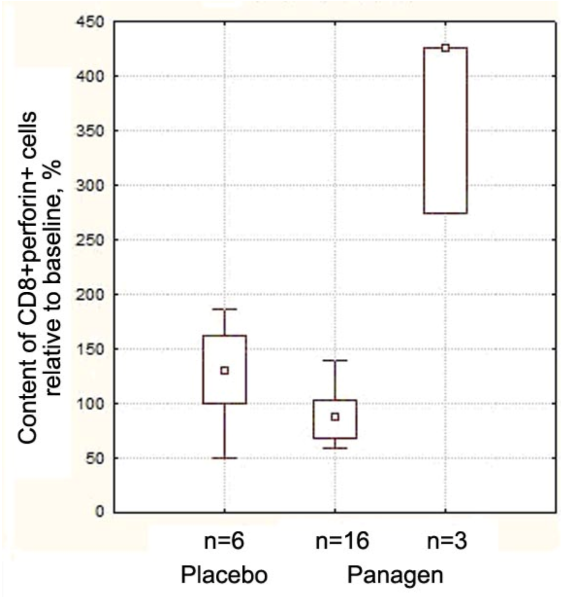

Figure 4. Relative abundance (%) of CD8+perforin+ cytotoxic T-cells in the peripheral blood of AC patients on day 21 after the 1st chemotherapy. Data were compared to the placebo group, whose minimum and maximum values were taken as a statistical norm. Median values, 25-75% quartile range (box) and min/max range (whiskers) are shown for each group; n – the number of patients per group.

We observed that AC regimen resulted in weaker stimulation of cytotoxic T-cell expansion in the control points chosen for analysis, as compared to the FAC regimen. On day 21 after the first round of chemotherapy, 3 patients out of 19 (i.e. 16%) displayed a pronounced increase in cytotoxic CD8+ perforin T cell counts. Importantly, this group of patients had a 300-400% increase relatively to the starting value, whereas the placebo patients showed only 185% increase in this measurement point. Combined number of patients whose CD8+ perforin+ T-cell counts are significantly higher than those observed in the reference group, is three.

Analysis of relative amount of regulatory T cells in the peripheral blood of patients recruited to the study of leukostimulatory activity of Panagen in stage II-IV breast cancer

CD4+CD25+CD127– T-regs hold a special place in induction of tumor resistance to immune surveillance. Such T-regs have a central role in controlling adaptive immunity and peripheral tolerance. Presence of these cells is a hallmark of breast, colorectal, lung and

pancreatic cancers, and is associated with poor prognosis. T-reg cells stem from the same progenitors as T helpers, and are induced by excessive TGF-β, IL-10 and VEGF signaling. Secretion of TGF-β and IL-10 by CD4+CD25+CD127 T-regs results in suppression of IL-2 expression by effector T cells. T-regs may also eliminate effector T cells via CD95 binding. T-regs may also secrete a range of cytokines inducing DC tolerance, and so DCs fail to express co-stimulatory molecules and are unable to stimulate naïve T cells. Furthermore, T-regs may induce secretion of inhibitory cytokines by the activated DCs (Fallarino *et al.*, 2004; Lange *et al.*, 2007; Yamazaki *et al.*, 2007; Гранов, Молчанов, 2008; Askenasy *et al.*, 2008). Thus, dramatic decrease in CD4+CD25+CD127– T-reg counts may argue for shifting the balance between the tumor and the anticancer immunity in favor of the latter.

Below we present quantitative analysis of CD4+CD25+CD127– T-regs in the peripheral blood of patients participating in Panagen clinical trial (**Table 6, Figure 5**). In this analysis, we considered Panagen patients as "responders", if their CD4+CD25+CD127– T-reg counts were lower than on day 0. We had a select group of patients, whose T-reg counts were >5-fold lower than on day 0 (**Table 6)**. The other group displayed a pronounced increase in T-reg counts.

Table 6. Percentage of CD4+CD25+CD127– T-regs in peripheral blood samples of patients. Median values in "Panagen-responders" subgroup that are significantly lower than the placebo values are shown in blue and marked with an asterisk (*) (p<0.11, Wikcoxin-Mann-Whitney test).

	**Absolute values, % cells in the blood sample**			**Relative values, % normalized to the starting value**	
	Day 0	Day 21 after the 1st CT	Day 21 after the 3rd CT	Day 21 after the 1st CT	Day 21 after the 3rd CT
**Panagen**					
02-01	4	8	8	200.0	200.0
02-02	11	0,12	9	1.1	81.8
02-03	12	0,54	14	4.5	116.7
02-04	10	9		90.0	
02-05	5	9	8	180.0	160.0
02-06	7	12	18	171.4	257.1
02-08	4	7	12	175.0	300.0
02-09	6	7	5	116.7	83.3
02-10	10	12	6,2	120.0	62.0

02-11	6	1	8	16.7	133.3
02-14	11	14	13	127.3	118.2
02-15	13	9	9	69.2	69.2
Median value				**118.3**	**118.2**
Responders, %				42	36
Non-Responders, %				58	64
Median value (Responders)				16.7*	75.5
Median value (Non-responders)				171.4	160.0
Total responders, %				**58**	
**Placebo**					
02-07	7	10	14	142.9	200.0
02-12	14	12	5	85.7	35.7
02-13	16	14	10	87.5	62.5
Median value				**87.5**	**62.5**

When using a second type of analysis, three distinct patient groups are clearly formed (**Figure 5**). The first is shaped by the patients having cell count values within the norm. The second and the third groups encompass the patients whose cell counts were significantly lower or higher than the norm, respectively.

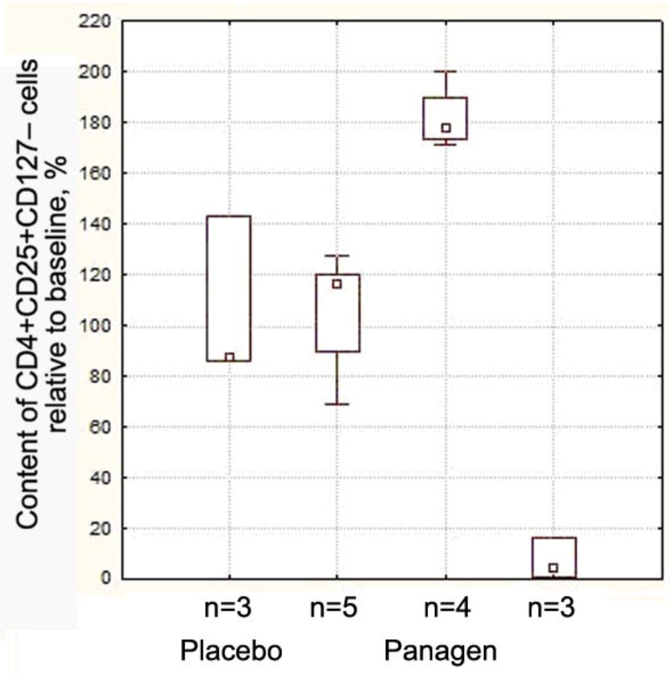

Figure 5. Relative abundance (%) of CD4+CD25+CD127– T-regs in peripheral blood of patients on day 21 after the 1st CT. Data were compared to the placebo group, whose minimum and maximum values were taken as a statistical norm. Median values, 25-75% quartile range (box) and min/max range (whiskers) are shown for each group; n- the number of patients per group.

CP is known to selectively inhibit proliferation of T-regs and to abrogate their suppressive activity (Ghiringhelli *et al.*, 2004; Ikezawa *et al.*, 2005; Lutsiak *et al.*, 2005; Motoyoshi *et al.*, 2006; Taieb *et al.*, 2006; Bopp *et al.*, 2010). This explains lower T-reg counts in a fraction of placebo-group patients. Nonetheless, the effects of Panagen (as measured on day 21 post 1st CT) are suggestive of a several-fold decrease in T-reg cell counts in a fraction of patients. This may indicate that the tumor becomes available for attack by activated cytotoxic T-cells, whose numbers are significantly increased at this measurement point.

It should also be noted that several Panagen-group patients display significantly higher values than what is found in the reference sampling, even though one of placebo patients also showed very high T-reg counts. Thus, Panagen can be viewed as a double-edged sword. In some patients, when it acts in synergy with CP, it boosts elimination of T-regs, whereas in other patients it stimulates T-reg proliferation in the context of leukostimulatory activity.

Indeed, because our sampling size was small and the results are contradictory further efforts are needed to better understand how Panagen modulates CD4+CD25+CD127– T-regs in a clinical setting.

Conclusions

1. Our analysis demonstrates that in the course of therapy the population of circulating plasmacytoid and myeloid DCs is expanded in a fraction of patients. Whether the patient responds to Panagen (i.e. whether it has more DCs) at any given measurement point, is highly individual.
2. In FAC-treated patients, when data are combined for both control points (on day 21 after the first and third CT) and compared to the reference group, 8 out of 12 Panagen-receiving patients (67%) responded to treatment, as assayed by expansion of CD8+perforin+ cytotoxic T-cells. In AC-treated patients, the pattern is less pronounced, and only 3 out of 19 patients responded (16%).
3. Panagen had positive and negative effects on the percentage of CD4+CD25+CD127– T-regs in peripheral blood samples of patients. In some cases (42% patients after the first round of CT and 36% patients after the third CT) it acted synergistically with CP and significantly enhanced elimination of T-regs (down to 16.7% of the starting level after the first CT). In the rest of the patients (58% patients after the first CT and 64%

after the third CT), Panagen stimulated proliferation of T-regs, which is attributable to its general leukostimulatory activity.

Our experiments thus demonstrate that Panagen effectively protects the cells of innate anticancer immunity from cytostatic drugs and stimulates development of adaptive immune response throughout the three consecutive courses of chemotherapy, when Panagen is combined with CP and DR to treat grade II-IV breast cancer patients in the clinical setting.
